# EETs Reduction Contributes to Granulosa Cell Senescence and Endometriosis‐Associated Infertility via the PI3K/AKT/mTOR Signaling Pathway

**DOI:** 10.1002/advs.202505656

**Published:** 2025-08-19

**Authors:** Xiang Lin, Weijia Gu, Xiaomei Tong, Mengru Lai, Yi Zhang, Na Liu, Xiaoying Jin, Chao Li, Dong Huang, Feng Zhou, Yongdong Dai, Songying Zhang

**Affiliations:** ^1^ Assisted Reproduction Unit Department of Obstetrics and Gynecology Sir Run Run Shaw Hospital Zhejiang University School of Medicine No. 3 Qingchun East Road, Shangcheng District Hangzhou 310016 China; ^2^ Key Laboratory of Reproductive Dysfunction Management of Zhejiang Province No. 3 Qingchun East Road, Shangcheng District Hangzhou 310016 China

**Keywords:** 14, 15‐EET, cellular senescence, endometriosis, EPHX2, EZH2/H3K27Me3, ovary granulosa cell, PI3K/AKT/mTOR signaling pathway, ROS

## Abstract

We aimed to examine abnormal oxidative lipid levels and their related mechanisms in EM‐associated infertility. Through liquid chromatography tandem mass spectrometry analysis, decreased levels of epoxyeicosatrienoic acids (EETs), which have antioxidant and anti‐senescence effects are observed, in EM patient follicular fluid samples. EET levels are positively correlated with in vitro fertilization outcomes. Lower 14, 15‐EET concentrations led to a decreased GC antioxidant capacity, reduced ATP production, reactive oxygen species (ROS) accumulation in oocytes, and abnormal cumulus‐oocyte complex (COC) expansion, ultimately resulting in decreased fertility. Elevated soluble epoxide hydrolase (EPHX2) expression in EM‐GCs is the main reason for EET reduction in EM follicular fluid. Inhibiting EPHX2 in vivo or in vitro can reverse these observed abnormalities by upregulating EETs. 14, 15‐EET treatment alleviated GC senescence and improved fertility by inhibiting excessive PI3K/AKT/mTOR signaling pathway activation in EM‐GCs, with BEZ‐235‐mediated inhibition of this pathway significantly alleviating ROS‐induced cell senescence and abnormal COC expansion. Oxidative stress‐induced decreased EZH2/H3K27Me3 histone methylation led to elevated EPHX2 expression patterns in EM‐GCs. Decreased 14, 15‐EET levels resulted in ROS accumulation, reduced EZH2 enzymatic activity, less EPHX2/H3K27Me3 histone methylation, and increased EPHX2 protein expression levels, which further reduced 14, 15‐EET levels in a vicious feedback loop.

## Graphical abstract

1) 14, 15‐EET levels were decreased in ovarian granulosa cells, follicular fluid, peritoneal fluid from endometriosis patients. 2) EETs reduction in endometriosis follicular fluid aggravated granulosa cell senescence. 3) ROS reduced 14, 15‐EET levels by upregulating EPHX2 through a mechanism involving histone methylation modification. 4) 14, 15‐EET suppressed the overactivation of PI3K/AKT/mTOR signaling pathway in ovarian granulosa cells of endometriosis.

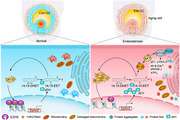

## 1 Introduction

Endometriosis (EM) is a common and prevalent disease among women of reproductive age, with its incidence in infertile women ranging from 20% to 50%.^[^ ^1^, ^2^ ^]^ Although the risk of infertility is up to four times higher in women with EM than in those without,^[^ ^3^ ^]^ the mechanism of EM‐associated infertility has not been fully elucidated. Current research suggests that the main causes of infertility in EM are abnormal follicle development and decreased oocyte quality.^[^ ^4^ ^]^

Oxidative stress is an important factor leading to abnormal follicular development and decreased oocyte quality in patients with EM.^[^ ^5^ ^]^ Oxidative stress refers to the imbalance between oxidation and antioxidant defense caused by reactive oxygen species (ROS) accumulation or decreased antioxidant levels in tissues or cells. Ovary granulosa cells (GCs) are an important component of follicles and provide nutrition and energy for oocyte development and maturation through gap junctions and paracrine mechanisms.^[^ ^6^ ^]^ Our previous studies have demonstrated that excessive oxidative stress can decrease the quantity and quality of oocytes through various mechanisms, including by inhibiting mitochondrial function, interfering with lipid metabolism, accelerating GC senescence, and reducing GC histone methylation levels.^[^ ^2^, ^7^, ^8^, ^9^ ^]^

Cellular senescence is a collective cell phenotype that occurs under various conditions, such as oxidative stress, radiation, and inflammation. It can manifest as irreversible cell cycle arrest, abnormal cell enlargement and flattening, reduced ATP generation, aberrant endoplasmic reticulum function, increased senescence‐associated β‐galactosidase (SA‐β‐gal) expression levels, and acquisition of senescence‐associated secretory phenotype.^[^ ^10^, ^11^ ^]^ Our previous studies have demonstrated increased cellular senescence in EM patient ovarian GCs.^[^ ^2^ ^]^ Therefore, alleviating GC senescence and improving oocyte quality are important approaches for improving the assisted reproductive technology (ART) outcomes of EM patients.

Oxidized lipids are products of the reaction between ROS and polyunsaturated fatty acids.^[^ ^12^ ^]^ There are various types of oxidized lipids that participate in multiple biological processes, such as cellular senescence, biomolecule biosynthesis, and immune regulation.^[^ ^13^ ^]^ Additionally, some oxidized lipids exhibit lipo‐toxicity to oocytes that cannot be reversed by conventional antioxidants.^[^ ^14^ ^]^ Current research mainly focuses on polyunsaturated fatty acid levels in plasma samples from infertile women, but the oxidized lipid profile in the follicular fluid (FF) of infertile women and its relationship with infertility remain unclear.^[^ ^14^ ^]^

We conducted targeted liquid chromatography tandem mass spectrometry (LC‐MS/MS) analysis of the FF, peritoneal fluid (PF), and GCs of infertile women to clarify the function of oxidized lipids. Our results showed that the levels of 15 oxidized lipids were decreased in EM patients compared with those in control women, including four epoxyeicosatrienoic acid (EET) isomers. Among these four EET isomers (5, 6‐EET, 8, 9‐EET, 11, 12‐EET, and 14, 15‐EET), 14, 15‐EET is the most studied and exhibits the strongest biological activity and the widest range of functions.^[^ ^15^ ^]^ Further research has suggested that the EET level decrease is closely associated with adverse ART outcomes in EM patients and may be involved in the development of EM‐associated infertility. In this study, we primarily focused on examining the cause of these decreased EETs in EM, as well as the role of 14, 15‐EET in GC senescence and follicle development.

Our data indicate that the decrease in 14, 15‐EET levels in EM follicles resulted from reduced H3K27Me3 histone methylation of the soluble epoxide hydrolase *Ephx2* gene, which encodes the main metabolic enzyme for EET. Additionally, lower 14, 15‐EET levels lead to reduced GC antioxidant capacity, resulting in ROS accumulation. This further inhibits EZH2 enzyme activity and reduces H3K27Me3 histone methylation modification of *Ephx2*. In summary, the feedback loop of ROS‐EZH2/H3K27Me3‐EPHX2‐EET forms a vicious cycle that can promote GC senescence and participate in EM‐associated infertility. Our data suggest that 14, 15‐EET can alleviate GC senescence and improve fertility by inhibiting abnormal activation of the PI3K‐AKT‐mTOR signaling pathway in EM GCs.

## 2 Results

### 2.1 14, 15‐EET Levels were Decreased in the FF and Ovary GCs of EM Patients

**Figure 1:**
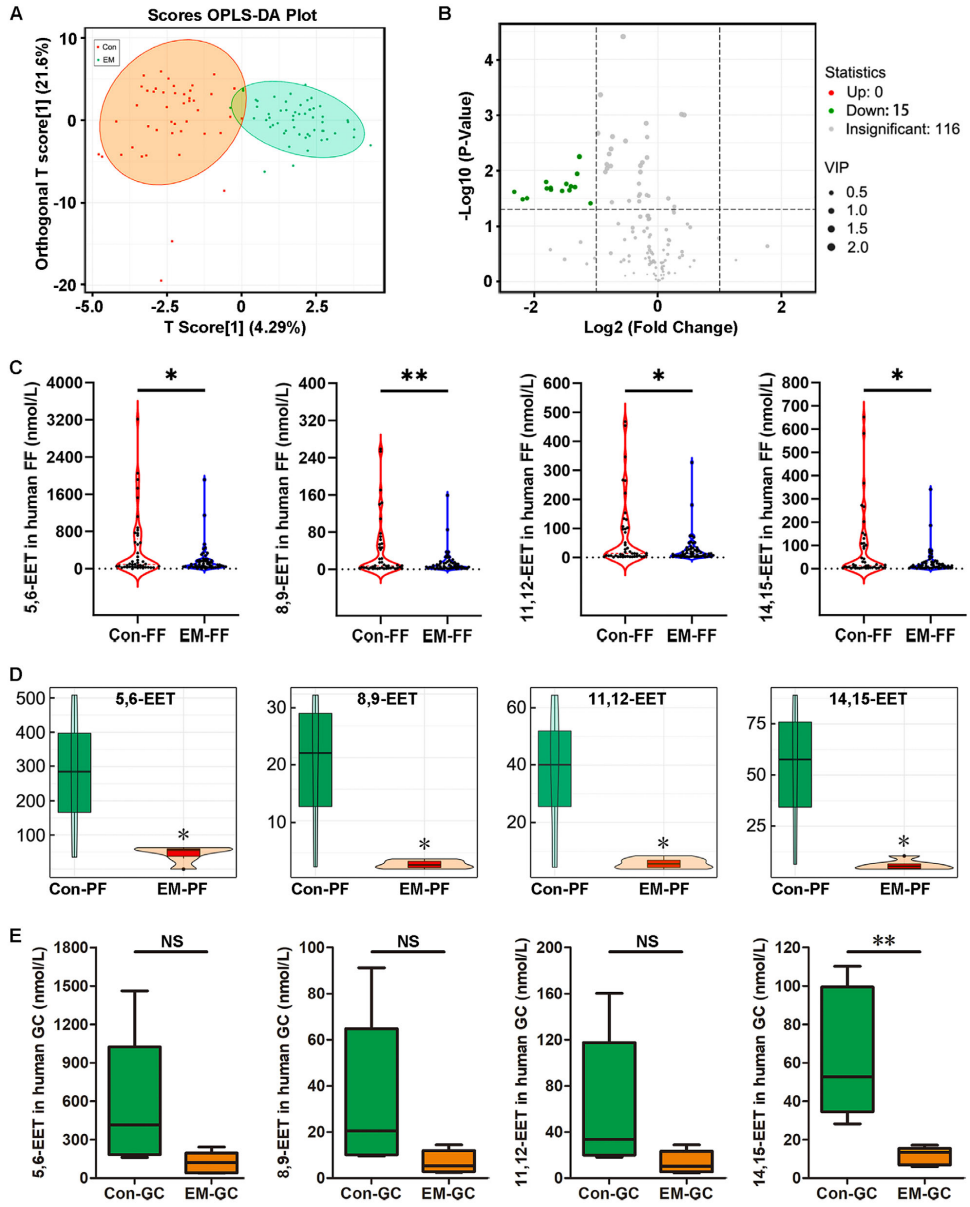
Epoxyeicosatrienoic acid (EET) concentrations were decreased in follicular fluid (FF), peritoneal fluid (PF), and ovary granulosa cells (GCs) of endometriosis (EM)‐associated infertility patients. A) Liquid chromatography tandem mass spectrometry (LC‐MS/MS) analysis was performed on 101 FF samples, as well as orthogonal partial least squares discriminant analysis (OPLS‐DA). The X‐axis represents the predictive principal components, showing the differences between groups. The Y‐axis represents the orthogonal principal components, showing the differences within groups. The percentage indicates the explanatory power of each component in the dataset. Each point in the graph represents a sample, with samples from the same group represented by the same color. The orange points represent 45 FF samples from the control group (Con), while the cyan points represent 56 FF samples from the EM group. B) A volcano plot of the differential metabolites between the 45 Con‐FF and 56 EM‐FF samples. Each point in the volcano plot represents a metabolite, with the X‐axis indicating the Log_2_ (Fold Change) of a metabolite between the two sample types and the Y‐axis indicating the ‐Log_10_ (*P*‐value). Green points represent differentially downregulated metabolites, red points represent differentially upregulated metabolites, and gray points represent metabolites that were detected but not significantly different.C) A violin plot depicting the levels of four EETs (5, 6‐EET, 8, 9‐EET, 11, 12‐EET, and 14, 15‐EET) between the 45 Con‐FF and 56 EM‐FF samples. The middle box represents the interquartile range, with the thin black lines extending from the box representing the 95% confidence interval. The black horizontal line in the middle represents the median and the outer shapes represent EET distribution density. Each point in the graph represents one sample. Unpaired *t*‐test, ^*^ *P* < 0.05, ^**^ *P* < 0.01. D) LC‐MS/MS analysis was performed on 10 PF samples. Box plots of 5, 6‐EET, 8, 9‐EET, 11, 12‐EET, and 14, 15‐EET in the four Con‐PF and six EM‐PF samples. Unpaired *t*‐test, ^*^ *P* < 0.05. E) Targeted LC‐MS/MS analysis was performed on 10 ovary GC samples. Box plots of 5, 6‐EET, 8, 9‐EET, 11, 12‐EET, and 14, 15‐EET in the five Con‐GC and five EM‐GC samples. Unpaired *t*‐test, ^**^ *P* < 0.01.

According to the results shown in Table S1 (Supporting Information), the body mass index and serum triglycerides levels were decreased in the EM‐associated infertility patients, while the IVF outcomes of the EM group were unfavorable. To explore the oxidized lipid profile in the FF of infertile women, we conducted targeted LC‐MS/MS on samples from 45 tubal infertile women and 56 EM‐associated infertile women. We detected the levels of 141 indicated oxidized lipids in FF. T scores of the predictive principal components and orthogonal principal components could visually separate the Con‐FFs and EM‐FFs as two distinct clusters (**Figure** **1**A). We found that 15 oxidized lipids were significantly downregulated in FF samples from EM patients compared with those in control FF samples (fold change > 2, corrected *P*‐value < 0.05) (Figure 1B; Figure S1A, Supporting Information). No oxidized lipid was significantly upregulated in the EM‐FF group. Among these 15 downregulated lipid metabolites (Figure S1A–C, Supporting Information), the EET family caught our attention because of the consistent decrease of its four isomers (5, 6‐EET, 8, 9‐EET, 11, 12‐EET, and 14, 15‐EET) in EM patient FF samples (Figure 1C; Figure S1C, Supporting Information). Moreover, 14, 15‐EET was the most significantly decreased EET in the EM‐FF (Figure 1C, Con‐FF vs EM‐FF, average 80.07 nm vs 27.28 nm, respectively, a nearly three‐fold difference). We further explored the profiles of these four EETs in human PF and GC. LC‐MS/MS was used to analyze four Con‐PF and six EM‐PF samples, which showed that 5, 6‐EET, 8, 9‐EET, 11, 12‐EET, and 14, 15‐EET were all decreased in EM (Figure 1D). Similar downward trends were also observed in EM‐GCs, but significant differences were only found for 14, 15‐EET (Figure 1E).

The “arachidonic acid metabolism pathway” was also downregulated in the EM group (Figure S1B, Supporting Information). Although arachidonic acid (AA), 5, 6‐dihydroxyeicosatrienoic acid (DHET), 8, 9‐DHET, 11, 12‐DHET, and 14, 15‐DHET were not changed between the EM and Con groups, 5, 6‐EET, 8, 9‐EET, 11, 12‐EET, and 14, 15‐EET were significantly reduced in EM‐FF samples (Figure S1C, Supporting Information). The correlation network diagram and radar map show the connections between the different metabolites (Figure S1D,E, Supporting Information), with the other 11 differentially decreased oxidized lipids listed in Figure S2A (Supporting Information). Moreover, no differences in follicular fluid EET levels were found among the four different EM stages (Figure S2B,C, Supporting Information). These results indicate the important role of EETs in EM‐associated infertility, especially 14, 15‐EET.

### 2.2 EET Levels were Positively Correlated with IVF Outcomes, and 14, 15‐EET Administration Alleviated ROS‐Induced Ovary GC Senescence

**Figure 2:**
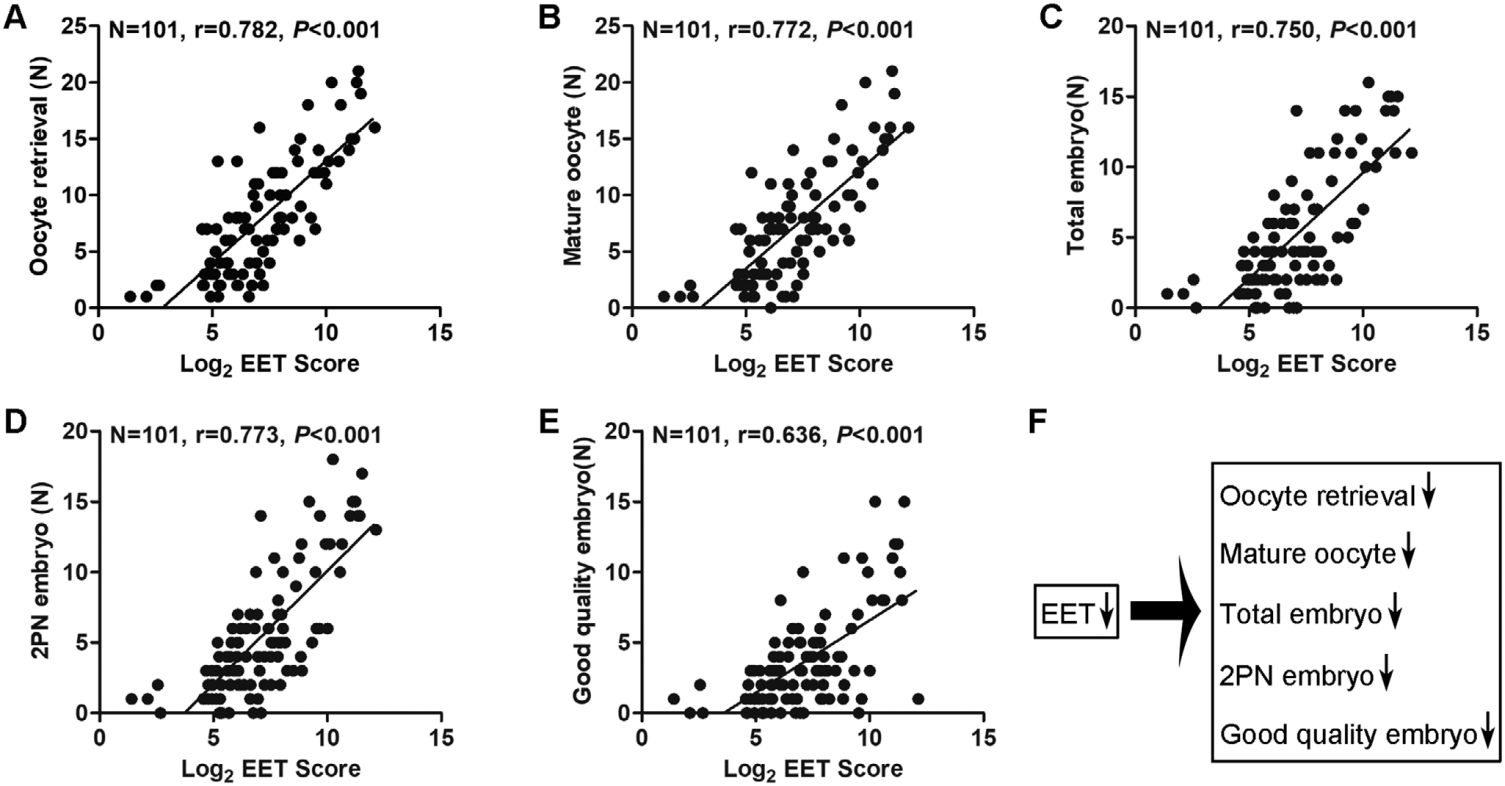
Decreased epoxyeicosatrienoic acid (EET) levels in follicular fluid (FF) samples correlated with reduced numbers of retrieved oocytes, mature oocytes, two pronucleus (2PN) embryos, total embryos, and good quality embryos. From the targeted liquid chromatography tandem mass spectrometry (LC‐MS/MS) quantitative analysis results in the 101 FF samples, we summed the values of the 5, 6‐EET, 8, 9‐EET, 11, 12‐EET, and 14, 15‐EET levels in FF to obtain the EET Score. A correlation analysis was then conducted with clinical data. A) Scatter diagram showing the linear regression and significant *Pearson* correlation of the oocyte retrieval number with the EET Score in 101 FF samples from the LC‐MS/MS analysis results (*n* = 101); r = 0.782, *P* < 0.001. B) Scatter diagram showing the linear regression and significant *Pearson* correlation of the mature oocyte number with the EET Score in FF samples (*n* = 101); r = 0.772, *P* < 0.001. C) Scatter diagram showing the linear regression and significant *Pearson* correlation of the total embryo number with the EET Score (*n* = 101); r = 0.750, *P* < 0.001. D) Scatter diagram showing the linear regression and significant *Pearson* correlation of the 2PN embryo number with the EET Score (n = 101); r = 0.773, *P* < 0.001. E) Scatter diagram showing the linear regression and significant *Pearson* correlation of the good quality embryo number with the EET Score (*n* = 101); r = 0.636, *P* < 0.001. F) Summary of the relationships between EET levels and in vitro fertilization (IVF) outcomes.

To explore the function of EETs in EM‐associated infertility, we incorporated the levels of the four EETs together and overlaid them to obtain an EET score, which was associated with IVF outcomes. From the LC‐MS/MS analysis results, we found significant correlations between the EET score and IVF outcomes (**Figure** **2**A–E). Linear regression analysis showed positive correlations between the EET score and oocyte retrieval number (r = 0.782), mature oocyte number (r = 0.772), total embryo number (r = 0.750), two pronucleus (2PN) embryo number (r = 0.773), and good quality embryo number (r = 0.636). These results implied that decreased EET levels in FF can contribute to adverse IVF outcomes (Figure 2F).

**Figure 3:**
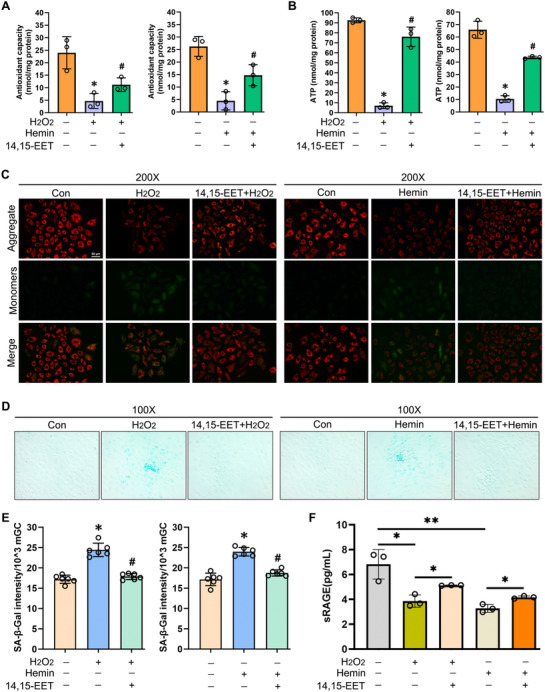
14, 15‐EET administration rescued the reactive oxygen species (ROS)‐induced antioxidant capacity reduction, ATP reduction, and mitochondrial transmembrane potential (MMP) decrease, and alleviated mouse granulosa cell (mGC) senescence. A) Antioxidant capacity assay of mGCs after 14, 15‐EET pre‐treatment for 24 h followed by H_2_O_2_ or hemin treatment for 24 h. Paired *t*‐test, ^*^ *P* < 0.05 (H_2_O_2_ or hemin vs Con), #*P* < 0.05 (14, 15‐EET + H_2_O_2_ vs H_2_O_2_ alone, or 14, 15‐EET + hemin vs hemin alone). () Intracellular ATP assay of mGCs after 14, 15‐EET pre‐treatment for 24 h followed by H_2_O_2_ or hemin treatment for 24 h. Paired *t*‐test, ^*^ *P* < 0.05 (H_2_O_2_ or hemin vs Con), #*P* < 0.05 (14, 15‐EET + H_2_O_2_ vs H_2_O_2_ alone, or 14, 15‐EET + hemin vs hemin alone). C) JC‐1‐based immunofluorescence assay of mGCs, with representative images of mGCs after 14, 15‐EET pre‐treatment for 24 h followed by H_2_O_2_ or hemin treatment for 24 h. Red represents the JC‐1 aggregate signal and green represents the JC‐1 monomer signal; scale bar 50 µm, original magnification: 200×. D) Senescence‐associated β‐galactosidase (SA‐β‐gal) staining assay of mGCs after 14, 15‐EET pre‐treatment for 24 h followed by H_2_O_2_ or hemin treatment for 24 h. Magnification, 100×. E) SA‐β‐gal quantitative assay of mGCs after 14, 15‐EET pre‐treatment for 24 h followed by H_2_O_2_ or hemin treatment for 24 h. Paired *t*‐test, ^*^ *P* < 0.05 (H_2_O_2_ or hemin vs Con), #*P* < 0.05 (14, 15‐EET + H_2_O_2_ vs H_2_O_2_ alone, or 14, 15‐EET + hemin vs hemin alone). F) soluble Receptor for Advanced Glycosylation End products (sRAGE) enzyme‐linked immunosorbent assay (ELISA) in mGC culture supernatants after 14, 15‐EET pre‐treatment for 24 h followed by H_2_O_2_ or hemin treatment for 24 h. Paired *t*‐test, ^*^ *P* < 0.05, ^**^ *P* < 0.01.

Many studies have reported that excessive oxidative stress and GC senescence are important factors contributing to the development of EM‐related infertility.^[^ ^2^, ^8^, ^9^ ^]^ Cumulus GCs from EM patients show increased ROS and abnormal mitochondrial function.^[^ ^2^ ^]^ To clarify the function of 14, 15‐EET in ovary GCs, we used H_2_O_2_ or hemin to induce oxidative stress in mGCs. The H_2_O_2_ or hemin treatment significantly suppressed the mGC antioxidant capacity, while 14, 15‐EET pre‐treatment 24 h before oxidative stress induction partially rescued this decreased antioxidant capacity (**Figure** **3**A; Figure S3A–C, Supporting Information). Furthermore, 100 nm 14, 15‐EET pre‐treatment for 24 h could significantly alleviate the mGC senescence induced by H_2_O_2_ or hemin (Figure S3A–C, Supporting Information). Because 200 nm 14, 15‐EET also effectively alleviated this senescence, we adhered to the principle of lowest effective concentration and chose 100 nm as the experimental concentration for the further experiments. ATP levels were reduced under oxidative stress conditions compared with those under normal conditions, while 14, 15‐EET pre‐treatment before oxidative stress induction significantly increased ATP levels compared with H_2_O_2_ or hemin treatment only (Figure 3B). JC‐1‐based IF assay results showed stronger green JC‐1 monomer signal in the H_2_O_2_ or hemin treatment group compared with that in the control group, while the green fluorescence intensity in the 14, 15‐EET pre‐treatment group was weaker than in the H_2_O_2_ or hemin treatment group (Figure 3C). These results indicated that 14, 15‐EET pre‐treatment could alleviate the mitochondrial dysfunction caused by excessive oxidative stress via enhancing the antioxidant capacity and increasing the ATP levels of mGCs.

Furthermore, SA β‐gal assays revealed increased SA β‐gal activity in the H_2_O_2_ or hemin treatment group compared with that in the control group, while 14, 15‐EET pre‐treatment before oxidative stress induction significantly decreased SA β‐gal activity relative to H_2_O_2_ or hemin treatment only (Figure 3D,E). The IF results revealed that expression levels of the aging marker protein γ‐H2A.X were upregulated in the H_2_O_2_ or hemin treatment group compared with those in the control group, while 14, 15‐EET pre‐treatment before oxidative stress induction decreased γ‐H2A.X staining relative to H_2_O_2_ or hemin treatment only (Figure S4A, Supporting Information). Flow cytometry‐based apoptosis analyses showed that cell apoptosis levels were also markedly increased in the H_2_O_2_ or hemin treatment group compared with those in the control group (Figure S4B,C, Supporting Information), while the mGC apoptosis levels were decreased following 14, 15‐EET pre‐treatment before oxidative stress induction. Moreover, the sRAGE levels typically decrease during cellular senescence.^[^ ^2^ ^]^ Our ELISA results showed that H_2_O_2_ or hemin treatment suppressed sRAGE expression, while 14, 15‐EET pre‐treatment before oxidative stress induction partially rescued the decreased sRAGE (Figure 3F). These results suggested that 14, 15‐EET could alleviate ROS‐induced senescence in ovary GCs.

### 2.3 Decreased Ezh2 Activity in EM GCs Upregulated EPHX2 Expression Levels by Decreasing H3K27Me3 Modification of the Ephx2 Gene Promoter

**Figure 4:**
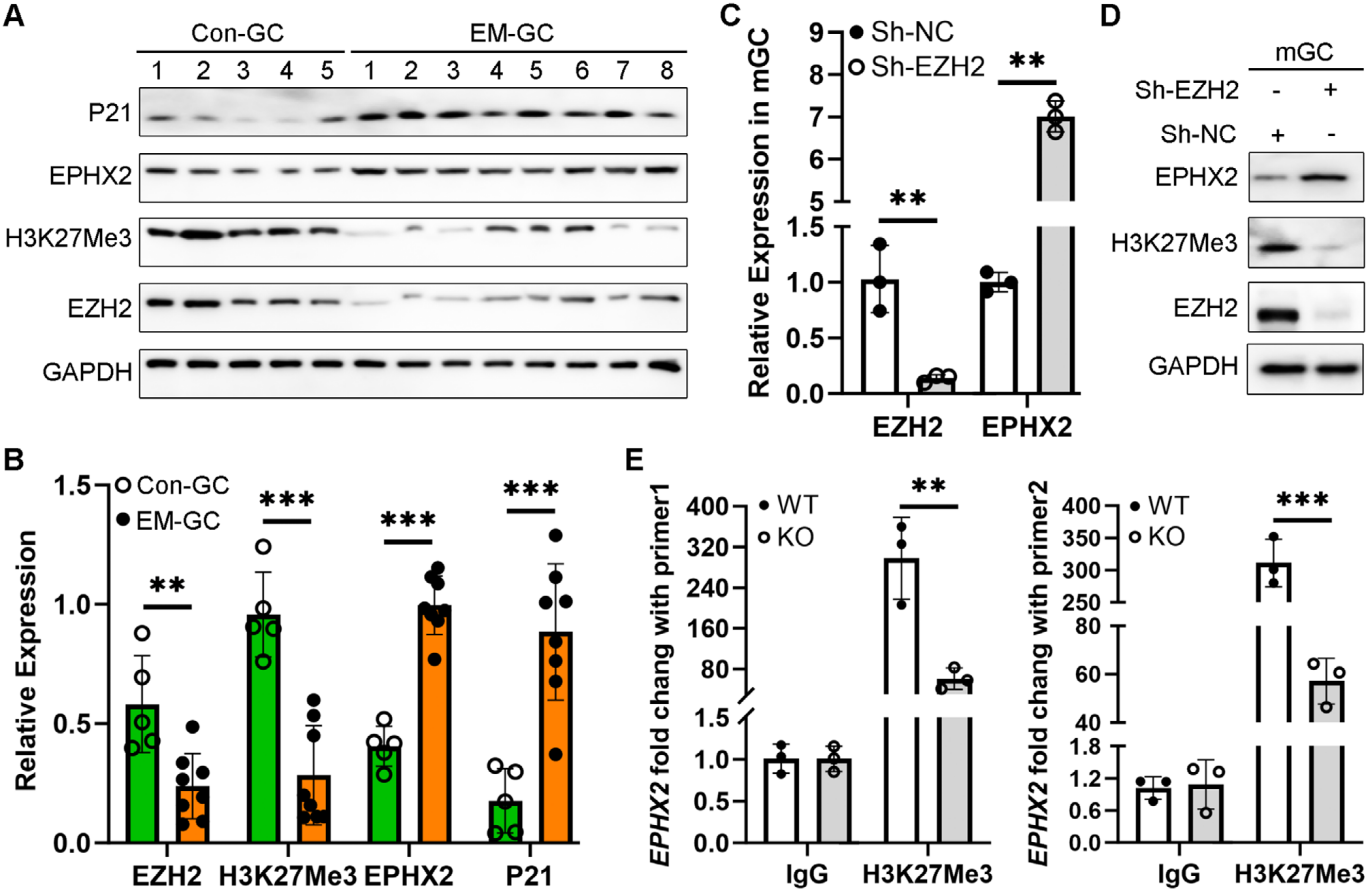
Decreased EZH2/H3K27Me3 histone methylation led to increased EPHX2 expression levels in endometriosis ovary granulosa cells (EM‐GCs). A) Western blot analysis of the indicated proteins from five Con‐GC and eight EM‐GC samples. B) Protein quantification analysis of the indicated proteins from five Con‐GC and eight EM‐GC samples. Unpaired *t*‐test, ^**^ *P* < 0.01, ^***^ *P* < 0.001. C) qRT‐PCR results of *Ezh2* and *Ephx2* mRNA expression levels in primary mouse GCs (mGCs) transduced with lentiviral vectors expressing a short hairpin RNA (shRNA) targeting EZH2 (Sh‐EZH2) or negative control (Sh‐NC) for 24 h. Paired t‐test, ^**^ *P* < 0.01. D) Western blot analysis of EZH2, H3K27Me3, and EPHX2 protein levels in mGCs after treatment with Sh‐EZH2 or Sh‐NC for 48 h. E) Mice with GC‐specific knockout of *Ezh2* (KO mice) were generated by crossing *Cyp19a1‐Cre* mice with *Ezh2* ^flox/flox^ mice, as described previously.^[^ ^9^ ^]^ ChIP‐PCR data showing the fold change of the immunoprecipitated *Ephx2* mRNA in freshly collected mGCs extracted from wild‐type (WT) and KO mice at HCG‐4 h (*n* = 20 for WT, *n* = 21 for KO). ChIP‐qPCR results showing *Ephx2* mRNA enrichment after IP with an anti‐H3K27Me3 antibody or control IgG using two different primers (primer 1 and primer 2). ChIP‐PCR data are shown as the mean ± standard deviation (SD) of three independent experiments. Paired t‐test, ^**^ *P* < 0.01, ^***^ *P* < 0.001.

As shown in Figure S5A (Supporting Information), AA is generally converted to EETs by cytochrome P450 monooxygenases. These EETs are then metabolized by soluble epoxide hydrolase (EPHX2) into the less active DHETs.^[^ ^16^ ^]^ From our previous abundant transcriptomic sequencing results of human GCs,^[^ ^2^ ^]^ we speculated that CYP3A5 may be the cytochrome P450 monooxygenase involved in converting AA into EETs in ovarian GCs. Further qRT‐PCR results found that CYP3A5 mRNA expression levels were similar between EM‐GCs and Con‐GCs (Figure S5B, Supporting Information). However, the mRNA expression levels of the rate‐limiting enzyme EPHX2, which metabolizes EETs into DHETs, were significantly increased in EM‐GCs (**Figure** **4**A,B; Figure S5B, Supporting Information). These data suggested that no abnormalities in the production of EETs, hence the reduced EET levels in EM‐FF may resulted from increased EPHX2 expression patterns in EM‐GCs.

In our previous studies, we found that excessive oxidative stress can suppress H3K27Me3‐based histone methylation modifications by inducing cytoplasmic accumulation of EZH2 in EM‐GCs.^[^ ^9^, ^17^ ^]^ As shown in Figure 4A,B, the levels of histone methyltransferase EZH2 and H3K27Me3 were significantly decreased in EM‐GCs, while the aging marker protein P21 had significantly increased expression levels in EM‐GCs. These data were consistent with our previous reports.^[^ ^2^, ^8^, ^9^ ^]^ To elucidate the reasons for the elevated EPHX2 expression levels in EM‐GCs, we considered the crucial role of histone methylation in EM‐GCs. The qRT‐PCR analysis of mGCs indicated reduced Ezh2 mRNA expression levels and increased Ephx2 mRNA expression levels in the Sh‐EZH2 group (Figure 4C). Importantly, Ezh2 knockdown via Sh‐EZH2 in vitro also increased the EPHX2 protein expression levels (Figure 4D; Figure S5C, Supporting Information). The mGCs from the GC‐specific knockout of Ezh2 mice (KO mice) showed decreased EZH2 and increased EPHX2 protein expression levels (Figure S5D,E, Supporting Information). ChIP‐sequencing was performed in mGCs from wild‐type (WT) or KO mice previously,^[^ ^9^ ^]^ with further ChIP‐PCR results confirming reduced binding of an anti‐H3K27Me3 antibody to the EPHX2 promoter (Figure 4E), as well as increased Ephx2 mRNA expression levels in the KO group (Figure 4C). Two ChIP‐PCR primer sets for two different loci validated the binding relationship between H3K27Me3 and EPHX2 (Figure 4E). These results indicated that decreased Ezh2 activity in EM‐GCs led to reduced H3K27Me3‐based histone methylation of the Ephx2 promoter region. This resulted in increased EPHX2 protein expression levels, which further promoted 14, 15‐EET metabolism and ultimately decreased the 14, 15‐EET levels in EM follicles.

### 2.4 EPHX2 Knockdown Increased EETs, which Partly Rescued ROS‐ Induced Ovary GC Senescence

**Figure 5:**
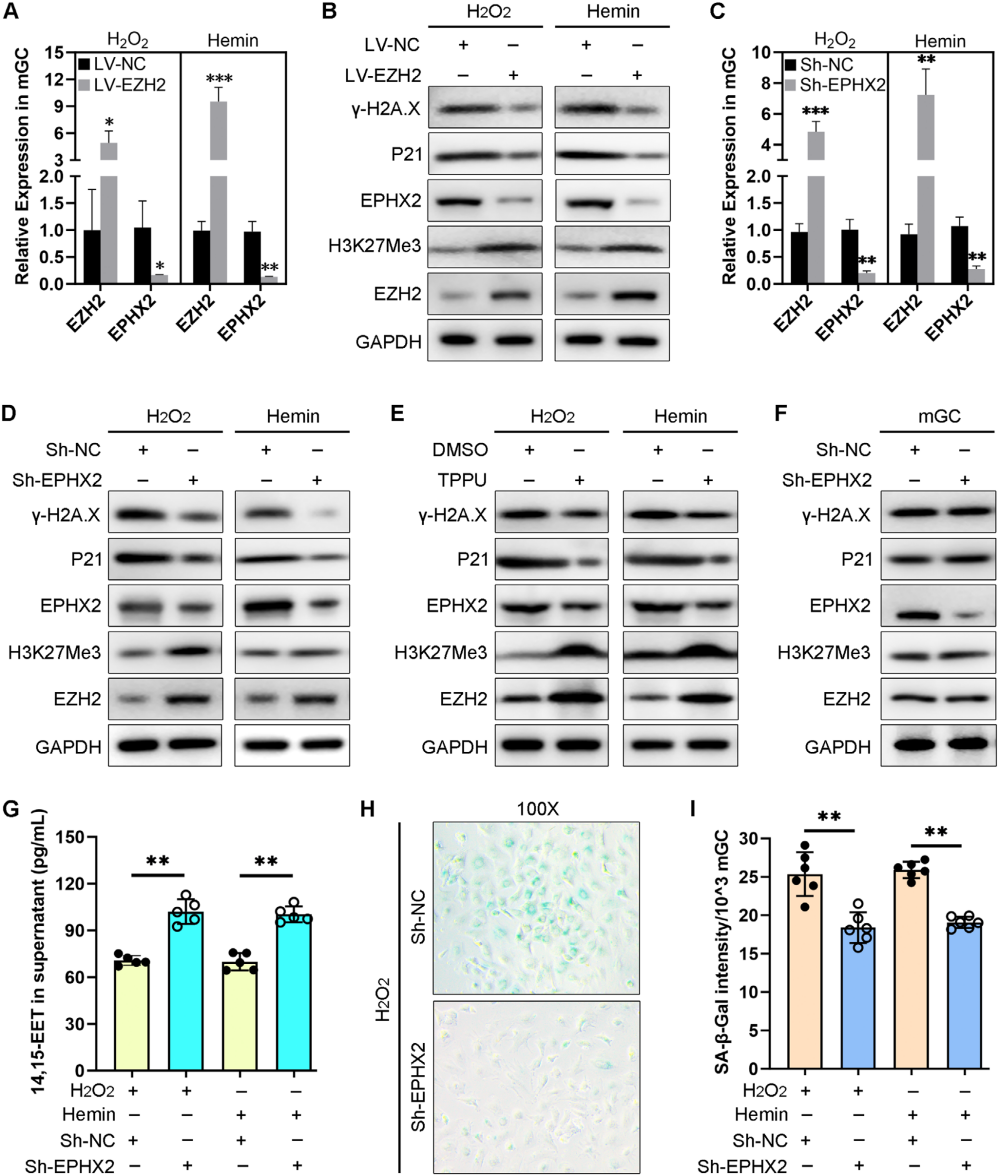
Inhibiting EPHX2 via lentiviral knockdown or TPPU treatment partially attenuated reactive oxygen species (ROS)‐induced cell senescence in vitro. Our previous work demonstrated that ROS can suppress histone methylation by inducing the nuclear to cytoplasmic distribution of EZH2 and inhibiting H3K27Me3 modifications in granulosa cells (GCs).^[^ ^2^, ^9^ ^]^ Treatment with 100 µm H_2_O_2_ or 10 µm hemin was used to induce an excessive oxidative stress microenvironment, which was maintained until the end of each experiment. A) qRT‐PCR results of *Ezh2* and *Ephx2* mRNA expression levels in mouse GCs (mGCs) after H_2_O_2_ or hemin pre‐treatment for 12 h followed by LV‐EZH2 or LV‐NC treatment for 24 h. Paired t‐test, ^*^ *P* < 0.05, ^**^ *P* < 0.01, ^***^ *P* < 0.001. B) Western blot analysis of the indicated proteins from mGCs after H_2_O_2_ or hemin pre‐treatment for 12 h followed by LV‐EZH2 or LV‐NC treatment for 48 h. C) qRT‐PCR results of *Ezh2* and *Ephx2* mRNA expression levels in mGCs after H_2_O_2_ or hemin pre‐treatment for 12 h followed by Sh‐EPHX2 or Sh‐NC treatment for 24 h. Paired t‐test, ^**^ *P* < 0.01, ^***^ *P* < 0.001. D) Western blot analysis of the indicated proteins from mGCs after H_2_O_2_ or hemin pre‐treatment for 12 h followed by Sh‐EPHX2 or Sh‐NC treatment for 48 h. E) Western blot analysis of the indicated proteins from mGCs after TPPU or DMSO pre‐treatment for 24 h followed by H_2_O_2_ or hemin treatment for 48 h. F) Western blot analysis of the indicated proteins in mGCs after transduction with Sh‐EPHX2 or Sh‐NC for 48 h. No excessive oxidative stress was induced in this experiment. G) 14, 15‐EET enzyme‐linked immunosorbent assay (ELISA) results in mGC culture supernatants after H_2_O_2_ or hemin pre‐treatment for 12 h followed by Sh‐EPHX2 or Sh‐NC treatment for 48 h. Paired *t*‐test, ^**^ *P* < 0.01. H) Representative SA‐β‐gal staining assay image for mGCs after H_2_O_2_ pre‐treatment for 12 h followed by Sh‐EPHX2 or Sh‐NC treatment for 24 h. Magnification, 100×. (I) SA‐β‐gal quantitative assay of mGCs after H_2_O_2_ or hemin pre‐treatment for 12 h followed by Sh‐EPHX2 or Sh‐NC treatment for 24 h. Paired *t*‐test, ^**^ *P* < 0.01.

To clarify the function of EPHX2 in EM‐GCs and EM‐associated infertility, we further overexpressed EZH2 after inducing oxidative stress. This resulted in decreased expression levels of EPHX2 and aging marker proteins (P21 and γ‐H2A.X) (**Figure** **5**A,B). Interestingly, under oxidative stress conditions, EPHX2 knockdown upregulated EZH2 expression levels, but suppressed P21 and γ‐H2A.X expression levels (Figure 5C,D). TPPU was then used to inhibit EPHX2 enzymatic activity. Our western blot analysis results also showed increased EZH2/H3K27Me3 levels but decreased aging protein expression levels under oxidative stress conditions (Figure 5E). However, under non‐oxidative stress conditions, EPHX2 knockdown did not affect the levels of EZH2/H3K27Me3 or the aging marker proteins (Figure 5F; Figure S5F, Supporting Information), which was consistent with previous research.^[^ ^18^ ^]^ Moreover, overexpression of EPHX2 under non‐oxidative conditions failed to generate clear senescence (Figure S5G, Supporting Information), with 14, 15‐EET supplementation under non‐oxidative conditions also showing negligible anti‐senescent effects (Figure S5H, Supporting Information). These results indicated that there is a reciprocal regulatory interaction between EZH2/H3K27Me3 and EPHX2 only under oxidative stress conditions.

Although no studies have reported the functions of 14, 15‐EET in GCs, EM, follicular development, and infertility, 14, 15‐EET is believed to inhibit cellular senescence through its direct antioxidant effects.^[^ ^15^, ^19^, ^20^ ^]^ Our ELISA results showed significantly increased 14, 15‐EET concentrations in mGC culture supernatants after Sh‐EPHX2 treatment for 48 h (Figure 5G). SA‐β‐gal assay results confirmed the anti‐senescence function of Sh‐EPHX2 in mGCs under oxidative stress conditions (Figure 5H,I). As a rate‐limiting enzyme in EET metabolism, we speculated that the anti‐senescence effect of EPHX2 knockdown under oxidative stress conditions may result from the increased 14, 15‐EET levels.

### 2.5 EPHX2 Overexpression Aggravated Ovary GC Senescence, while its Inhibitor TPPU Alleviated ROS‐Induced Cell Senescence In Vitro

**Figure 6:**
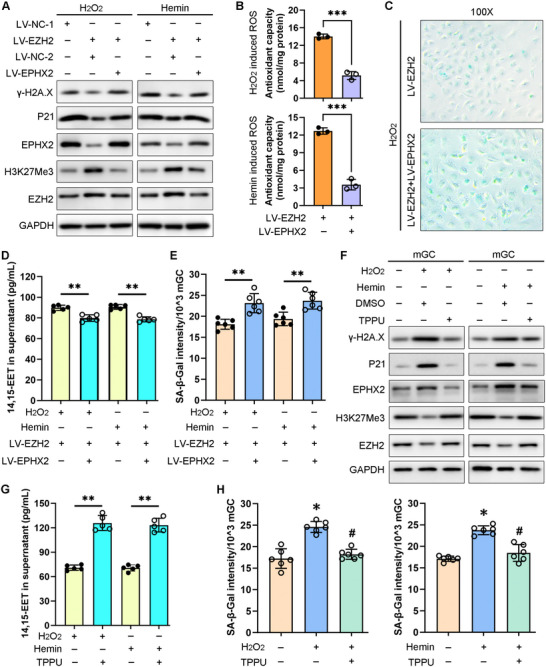
EPHX2 overexpression aggravated mouse granulosa cell (mGC) senescence via suppressing 14, 15‐EET levels, while the EPHX2 enzyme inhibitor TPPU rescued reactive oxygen species (ROS)‐induced cell senescence in vitro. A) Western blot analysis of the indicated proteins in mGCs after transduction with LV‐NC‐1 or LV‐EZH2 for 24 h followed by LV‐NC‐2 or LV‐EPHX2 treatment for 48 h. LV‐EZH2 is the lentiviral vector overexpressing EZH2; LV‐NC‐1 is the corresponding negative control. LV‐EPHX2 is the lentiviral vector overexpressing EPHX2; LV‐NC‐2 is the corresponding negative control. B) Antioxidant capacity assay of mGCs after LV‐EZH2 pre‐treatment for 24 h followed by LV‐EPHX2 treatment for 24 h. Paired *t*‐test, ^***^ *P* < 0.001. C) SA‐β‐gal staining assay of mGCs after LV‐EZH2 pre‐treatment for 24 h followed by LV‐EPHX2 treatment for 24 h. Magnification, 100×. D) Excessive oxidative stress was induced via 100 µm H_2_O_2_ or 10 µm hemin treatment in mGCs, which was maintained until the end of each experiment. 14, 15‐EET enzyme‐linked immunosorbent assay (ELISA) in mGC culture supernatants after LV‐EZH2 pre‐treatment for 24 h followed by LV‐EPHX2 treatment for 48 h. Paired *t*‐test, ^**^ *P* < 0.01. E) SA‐β‐gal quantitative assay of mGCs after LV‐EZH2 pre‐treatment for 24 h followed by LV‐EPHX2 treatment for 24 h under oxidative stress. Paired *t*‐test, ^**^ *P* < 0.01. F) Western blot analysis of the indicated proteins from mGCs after TPPU or DMSO pre‐treatment for 24 h followed by H_2_O_2_ or hemin treatment for 48 h. G) 14, 15‐EET ELISA in mGC culture supernatants after TPPU or DMSO pre‐treatment for 24 h followed by H_2_O_2_ or hemin treatment for 48 h. Paired *t*‐test, ^**^ *P* < 0.01. H) SA‐β‐gal quantitative assay of mGCs after TPPU pre‐treatment for 24 h followed by H_2_O_2_ or hemin treatment for 24 h. Paired *t*‐test, ^*^ *P* < 0.05 (H_2_O_2_ or hemin vs Con), #*P* < 0.05 (TPPU + H_2_O_2_ vs H_2_O_2_ alone, or TPPU + hemin vs hemin alone).

Western blot analysis results showed that EZH2 overexpression under oxidative stress conditions suppressed EPHX2, P21, and γ‐H2A.X expression, while overexpressing EPHX2 following LV‐EZH2 treatment reversed the reduction of the P21 and γ‐H2A.X protein expression levels (**Figure** **6**A). Under oxidative stress conditions, EPHX2 overexpression following LV‐EZH2 treatment suppressed the mGC antioxidant capacity compared with LV‐EZH2 treatment alone (Figure 6B). Under oxidative stress conditions, the SA‐β‐gal staining intensity was clearly increased in the EPHX2 overexpression group compared with that in the LV‐EZH2 treatment group (Figure 6C). 14, 15‐EET levels were decreased in the EPHX2 overexpression group compared with those in LV‐EZH2 treatment alone (Figure 6D), while SA‐β‐gal quantitative assays also revealed increased SA‐β‐gal activity in the EPHX2 overexpression group compared with that in the LV‐EZH2 treatment only group (Figure 6E).

TPPU is useful for investigating EPHX2 biology because the TPPU concentration in blood increases dose dependently within the treatment period to an almost steady state after eight days.^[^ ^21^, ^22^, ^23^ ^]^ Our results demonstrated that TPPU pre‐treatment before oxidative stress induction reversed the ROS‐induced cellular senescence and upregulated EZH2/H3K27Me3 levels (Figure 6F). Under oxidative stress conditions, ELISA data confirmed an increased 14, 15‐EET concentration in mGC culture supernatants following TPPU treatment (Figure 6G), which implied that TPPU exerts its anti‐senescence function by upregulating 14, 15‐EET levels. SA‐β‐gal quantitative assays further showed that TPPU pre‐treatment alleviated ROS‐induced cell senescence in mGCs (Figure 6H). These results collectively indicated that inhibiting EPHX2 activity could alleviate ROS‐induced mGC senescence via upregulating 14, 15‐EET in vitro.

### 2.6 EPHX2 Inhibitor TPPU Slowed the EM Mouse Fertility Decline by Alleviating GC Senescence In Vivo

**Figure 7:**
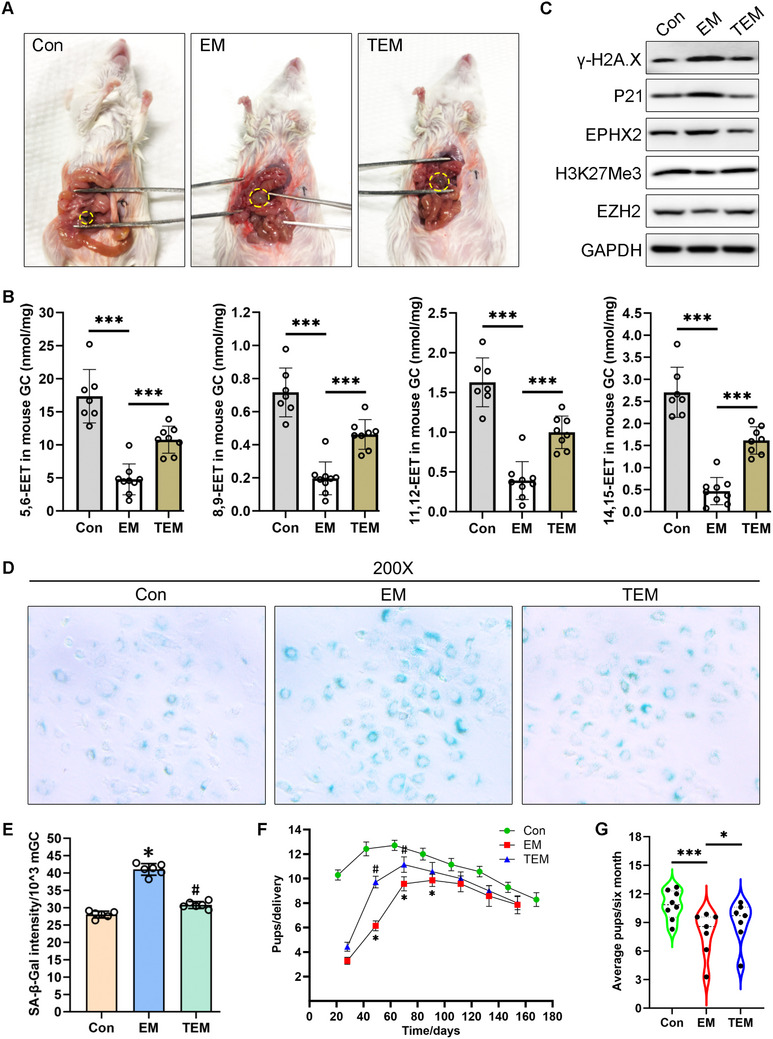
TPPU gavage rescued ovarian granulosa cell (GC) senescence in an endometriosis (EM) mouse model by upregulating EET levels. A) Representative visible lesions within the peritoneal cavity of EM mice and TEM mice (EM mouse that treated with TPPU gavage). No significant change was found in the ectopic cyst volume between the TEM and EM groups. For TEM mice, 3 mg kg^−1^ TPPU intragastric administration began on the 14th day after endometrium transplantation surgery and was continued until sample collection. B) Targeted liquid chromatography tandem mass spectrometry (LC‐MS/MS) analysis was performed on freshly extracted mouse GCs (mGCs) (*n* = 7 for Con group, *n* = 9 for EM group, *n* = 8 for TEM group). Unpaired t‐test, ^***^ *P* < 0.001. C) Western blot analysis of the indicated proteins in freshly extracted mGCs (*n* = 3 for Con group, *n* = 3 for EM group, *n* = 3 for TEM group). D) SA‐β‐gal staining assay of freshly extracted mGCs (*n* = 3 for Con group, *n* = 3 for EM group, *n* = 3 for TEM group). Magnification, 200×. E) SA‐β‐gal quantitative assay of freshly extracted mGCs (*n* = 6 for Con group, *n* = 6 for EM group, *n* = 6 for TEM group). Unpaired *t*‐test, ^*^ *P* < 0.05 (EM vs Con), #*P* < 0.05 (TEM vs EM). F) The development of mouse model fertility within six months of mating (*n* = 7 for Con group, *n* = 7 for EM group, *n* = 7 for TEM group). The horizontal axis shows the time (days) from mating and the vertical axis indicates the average pup number of each delivery (pups/delivery). Unpaired *t*‐test, ^*^ *P* < 0.05 (EM vs Con), #*P* < 0.05 (TEM vs EM). G) Violin plot of the average number of pups per delivery within six months (*n* = 7 for Con group, *n* = 7 for EM group, *n* = 7 for TEM group). One dot represents one delivery, with 8 deliveries occurring in the Con group and seven deliveries each occurring in the EM and TEM groups. Unpaired *t*‐test, ^*^ *P* < 0.05, ^***^ *P* < 0.001.

We next explored if TPPU treatment could affect cell senescence in vivo. The EM mouse model was established using six‐week‐old ICR mice as previously described.^[^ ^2^ ^]^ Figure S6A (Supporting Information) shows the detailed protocol and **Figure** **7**A shows the typical EM cysts after implantation for 4 weeks. The lesion sizes were similar between the EM mice and TEM mice (Figure S6B, Supporting Information). To explore if intragastric administration of 3 mg kg^−1^ TPPU resulted in clear drug toxicity, we also analyzed the body weight and ovary wet weight after TPPU treatment for 14 days. No significant difference in mouse body weight or ovary wet weight was found between the EM and TEM female mice (Figure S6C,D, Supporting Information). We also observed the estrous cycle of three EM and three TEM mice for 14 days via the direct smear method. Our results showed similar estrous cycle patterns between the EM and TEM mice (Figure S6E, Supporting Information).

LC‐MS/MS results showed that 5, 6‐EET, 8, 9‐EET, 11, 12‐EET, and 14, 15‐EET levels were all decreased in EM‐mGCs compared with those in the control group, while their levels were increased in mGCs collected from the TEM group compared with those in the EM‐mGCs (Figure 7B). Relative to the control group, mGCs from the EM group showed decreased EZH2 and H3K27Me3 levels but increased EPHX2, P21, and γ‐H2A.X expression levels (Figure 7C). The SA‐β‐gal staining intensity was clearly increased in EM‐mGCs compared with that in Con‐mGCs (Figure 7D). TPPU administration for two weeks significantly upregulated the EZH2 and H3K27Me3 levels, while it reversed the increased EPHX2, P21, and γ‐H2A.X expression levels in the EM group (Figure 7C, TEM vs EM). The TEM group mGCs showed decreased SA‐β‐gal activity compared with the EM group mGCs (Figure 7D,E). These data suggested a vital role for TPPU in alleviating mGC senescence in vivo, with this function potentially being achieved by upregulating EET levels.

Four weeks after the endometrium transplantation surgery (day 28), the mice (10 weeks old) were mated with fertile males to induce pregnancy. The development of mouse model fertility within six months is described in Figure 7F,G. The horizontal axis shows the time (days) from mating, while the vertical axis displays the average numbers of pups for each delivery (pups/delivery). Vaginal plugs were checked to confirm the mating day, with this day defined as day 0 (also the beginning of the horizontal axis). Each node on the fertility curve represents the average number of pups for all seven female mice in each group at the corresponding delivery (Figure 7F). The average number of pups produced by all seven female mice in each group at every delivery was calculated (Figure 7G). The Con group mice had eight deliveries within six months, while the EM group or TEM group mice only had seven deliveries (Figure 7F,G, each node on the fertility curve or each point on the violin plot refers to a delivery). The Con group mice took an average of 21 days to produce their first pups, while the EM or TEM group mice took 29 to 33 days to produce their first pups (see the location of the first node in Figure 7F). However, the interval time between the two adjacent nodes in the three groups was 21 days, indicating that no significant production time delay occurred after the first delivery. Collectively, these results indicated that the first litters were compromised in the EM mice, while TPPU administration rescued this fertility decline in the EM mice within the first two months. Although the fertility curves for the Con, EM, and TEM mice showed similar trends, the average number of pups per delivery decreased in the EM group compared with that in the control group, while TPPU administration slowed down this EM group fertility decline in vivo (Figure 7G).

### 2.7 EPHX2 Inhibitor TPPU Administration Improved COC Expansion in EM Mice and Suppressed ROS Accumulation in EM Oocytes

**Figure 8:**
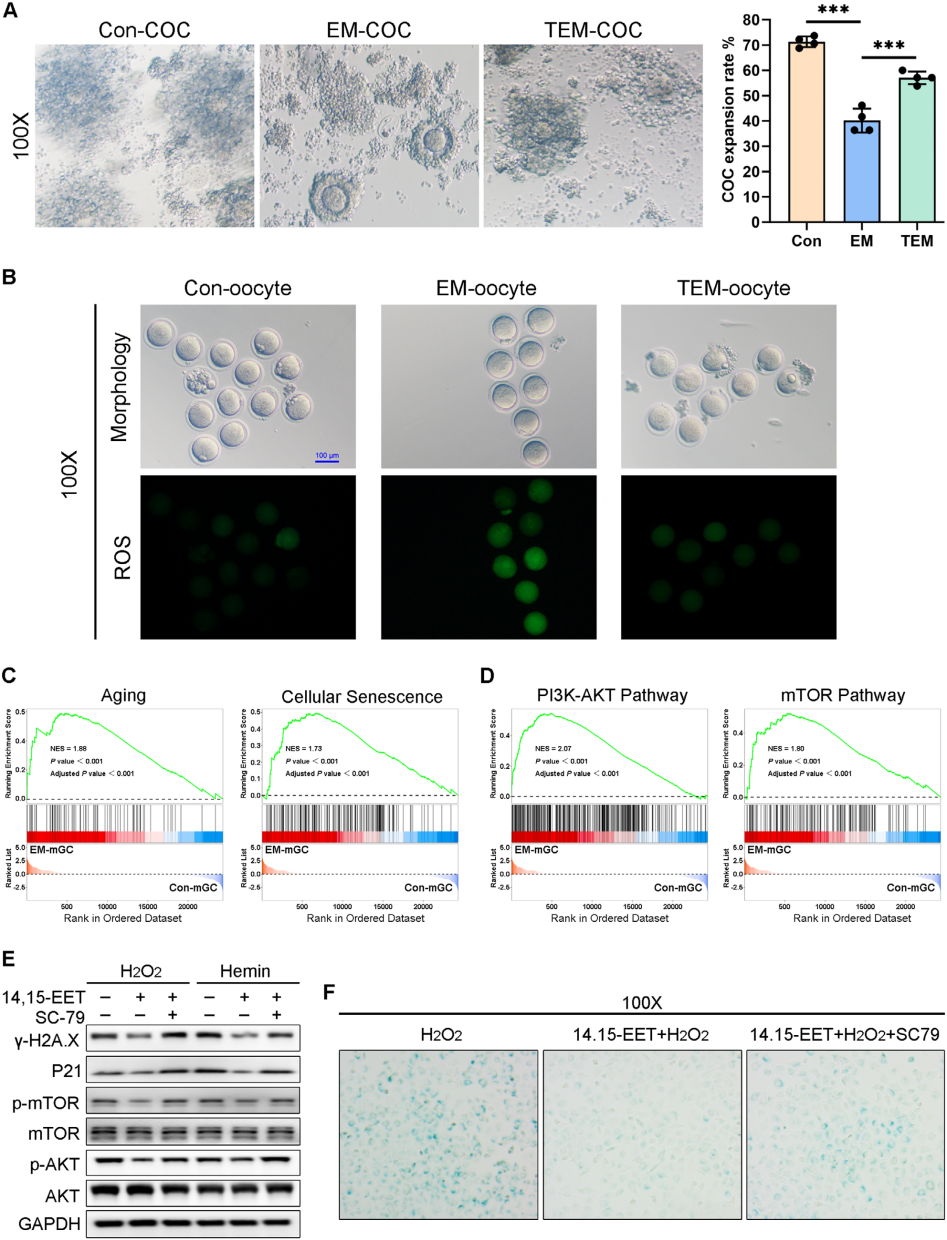
TPPU gavage improved the cumulus‐oocyte complex (COC) expansion rate of endometriosis (EM) model mice and alleviated the reactive oxygen species (ROS) levels of their oocytes. A) In vitro COC expansion assay results. The defective COC expansion in EM mice was partially rescued by TPPU treatment. Images shown in the left panel were acquired 12 h after COC culture in vitro. Magnification, 100×. The right panel shows the statistical analysis of the average COC expansion rates from four Con mice, four EM mice, and four TEM mice. Unpaired *t*‐test, ^***^ *P* < 0.001. B) ROS fluorescence staining (green) in MII oocytes from the Con, EM, and TEM group. Each group included oocytes from three different mice for analysis. Scale bar 100 µm, Magnification, 100×. C) RNA‐Sequencing results of mouse granulosa cells (mGCs) (*n* = 5 for Con group, *n* = 5 for EM group), with Gene Set Enrichment Analysis (GSEA) revealing an enrichment of functional genes from the “Aging” and “Cellular senescence” gene sets in the EM group compared with the Con group (with NES = 1.88, 1.73, respectively). NES, normalized enrichment score; false discovery rate (FDR) of all sets were less than 25%, and all *P*‐values were less than 0.01. D) GSEA revealed overactivation of functional genes from the “PI3K‐Akt signaling pathway” and “mTOR signaling pathway” gene sets in the EM group compared with the Con group (with NES = 2.07, 1.80, respectively). E) Western blot analysis of the indicated proteins after ROS induction, 14, 15‐EET pre‐treatment, and 14, 15‐EET pre‐treatment followed by AKT reactivation by 20 µm SC79. (F) SA‐β‐gal staining assay of mGCs after 14, 15‐EET pre‐treatment for 24 h followed by 20 µm SC79‐mediated AKT reactivation for another 24 h. Magnification, 100×.

Our previous study found that selective Ezh2 KO in mGCs resulted in multiple ovulation defects.^[^ ^9^ ^]^ Because TPPU administration increased EZH2 and H3K27Me3 levels under oxidative stress conditions, we further explored the impacts of EPHX2 inhibition on COC expansion and oocyte development. COC expansion was impaired in the EM group compared with that in the control group (**Figure** **8**A, the right side is a bar chart showing the COC expansion rates of four mice), while TPPU administration significantly improved the COC expansion rate in the TEM group compared with that in the EM group (Figure 8A). ROS levels were detected via IF assays, with our results showing stronger green signal in EM group oocytes compared with that in control group oocytes. The green fluorescence intensity in the TEM group was weaker than in the EM group (Figure 8B). These data indicated that TPPU administration in vivo could alleviate oxidative damage to oocytes and improve the COC expansion rate.

### 2.8 14, 15‐EET Alleviated GC Senescence by Suppressing Over‐Activation of the PI3K/AKT/mTOR Signaling Pathway in EM

To explore the GC pathological changes in EM, mGCs collected from five EM mice and five Con mice were used for RNA‐seq analysis. Gene Set Enrichment Analysis (GSEA) revealed that the functional genes from the “Aging” and “Cellular senescence” gene sets in the EM group were enriched compared with the genes in the Con group (Figure 8C). These results were consistent with those of our previous studies.^[^ ^2^, ^8^ ^]^ GSEA also revealed over‐activation of the PI3K‐Akt and mTOR signaling pathways in EM‐mGCs compared with the findings in Con‐mGCs (Figure 8D). To further establish causality between 14, 15‐EET and PI3K/AKT/mTOR pathway suppression, we reactivated AKT using SC79 after 14, 15‐EET treatment under oxidative stress conditions. Our results in Figure 8E,F show that 14, 15‐EET could alleviate the ROS‐induced GC senescence, but reactivation of AKT increased P21 and γ‐H2A.X protein expression levels and led to the recurrence of aging manifestations.

**Figure 9:**
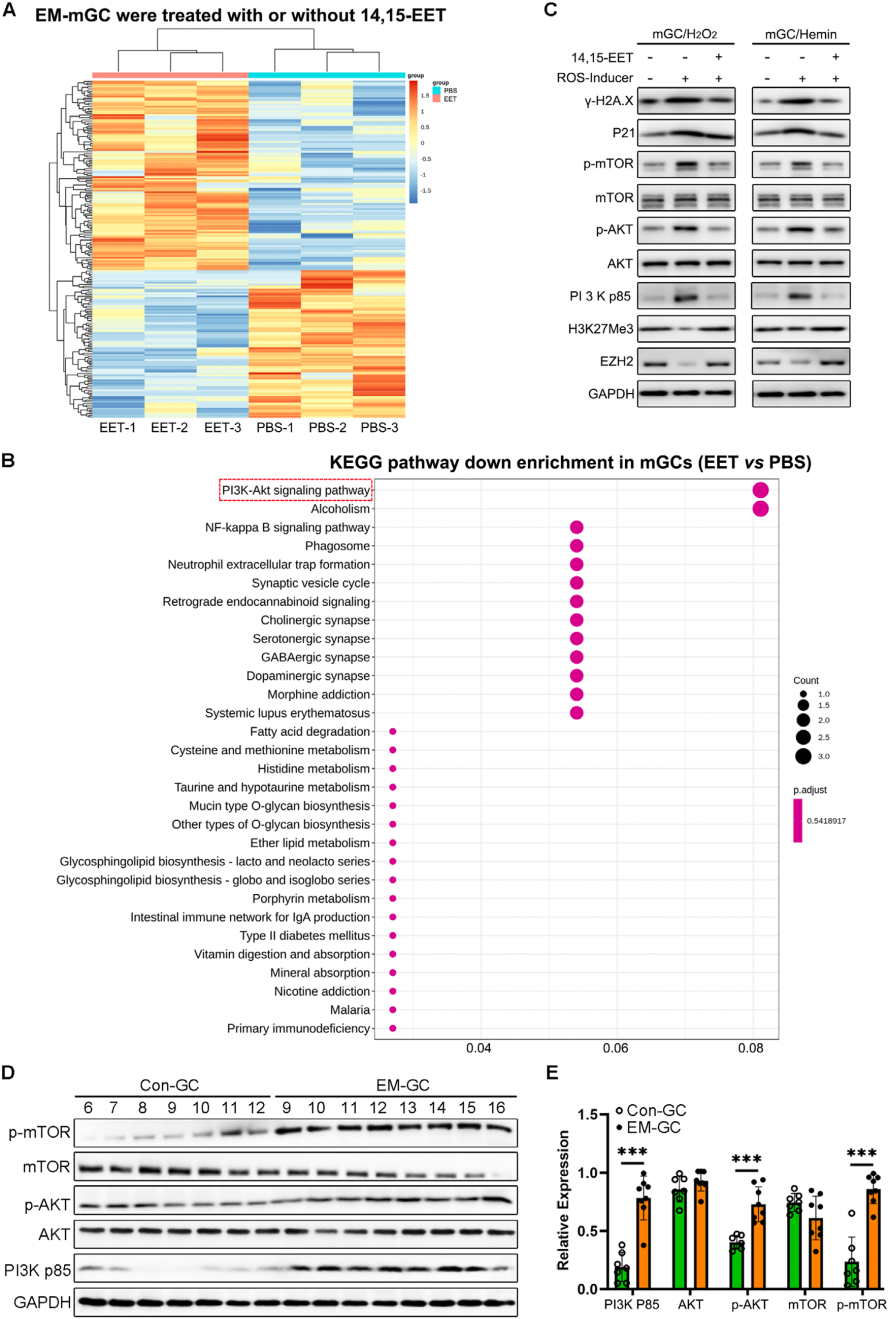
14, 15‐EET rescued reactive oxygen species (ROS)‐induced mouse granulosa cell (mGC) senescence in vitro via suppressing PI3K‐AKT‐mTOR signaling pathway overactivation. A) mGCs were collected from six endometriosis (EM) model mice and cultured in DMEM/F‐12 with 5% FBS for 24 h. Then, 100 nm 14, 15‐EET was added to the EET group for another 24 h, with an equivalent volume of PBS added to the control group (PBS). To explore the function of 14, 15‐EET in EM ovarian GCs, six groups of mGCs were collected for RNA‐sequencing analysis (*n* = 3 for PBS group, *n* = 3 for EET group). A heat map showing the differentially expressed genes, including 113 upregulated and 88 downregulated genes, in the EET group. B) The KEGG analysis results indicated significantly decreased enrichment of the “PI3K‐AKT signaling pathway” in mGCs after 14, 15‐EET treatment. C) Western blot analysis of the indicated proteins from mGCs after 14, 15‐EET pre‐treatment for 24 h followed by H_2_O_2_ or hemin treatment for 48 h. mGCs were extracted from immature ICR mice and oxidative stress was induced via 100 µm H_2_O_2_ or 10 µm hemin treatment. D) Western blot analysis of the indicated proteins from seven Con‐GC and eight EM‐GC samples. E) Protein quantification analysis of the indicated proteins from seven Con‐GC and eight EM‐GC samples. Unpaired *t*‐test, ^***^ *P* < 0.001.

To further illuminate the mechanism of 14, 15‐EET in EM‐associated infertility, we collected mGCs from EM mice, and 14, 15‐EET was used for co‐culture. RNA‐seq analysis was then performed on the mGCs collected from the 14, 15‐EET treatment group or normal culture group. Approximately 27873 genes were successfully annotated, with 201 genes found to be differentially expressed. A heat map was generated that shows the differentially expressed genes, which included 113 upregulated and 88 downregulated genes in the EET group (**Figure** **9**A). The Kyoto Encyclopedia of Genes and Genomes (KEGG) analysis results indicated significantly lower enrichment of the “PI3K‐AKT signaling pathway” in mGCs after 14, 15‐EET treatment (Figure 9B). H_2_O_2_ or hemin treatment significantly activated expression of PI3K p85, p‐AKT, and p‐mTOR in mGCs, while 14, 15‐EET pre‐treatment before oxidative stress induction partially reversed the over‐activation of the PI3K/AKT/mTOR signaling pathway (Figure 9C). Western blot analysis of seven Con‐GCs and eight EM‐GCs also showed that the PI3K/AKT/mTOR signaling pathway was over‐activated in EM samples (Figure 9D,E). These results collectively indicated the vital role of the PI3K/AKT/mTOR signaling pathway in EM‐associated infertility, implying that 14, 15‐EET may function by suppressing the over‐activation of this pathway in EM‐GCs.

### 2.9 Dual Pan‐Class I PI3K and mTOR Inhibitor BEZ‐235 Alleviated GC Senescence and Improved the COC Expansion Rate of EM Mice

The dual pan‐class I PI3K and mTOR inhibitor BEZ‐235 was used in vivo and in vitro to examine the function of the PI3K/AKT/mTOR signaling pathway in ROS‐induced cell senescence. The lesion sizes were similar between the EM mice and BEM mice (Figure S6F, Supporting Information). To explore whether intragastric administration of 20 mg kg^−1^ BEZ‐235 resulted in clear drug toxicity, we also analyzed the body weight and ovary wet weight after BEZ‐235 administration for 14 days. No significant difference in mouse body weight or ovary wet weight was found between the EM and BEM female mice (Figure S6G,H, Supporting Information). We also observed the estrous cycle of three EM and three BEM mice for 14 days via the direct smear method. Our results showed similar estrous cycle patterns between the EM and BEM mice (Figure S6I, Supporting Information).

**Figure 10:**
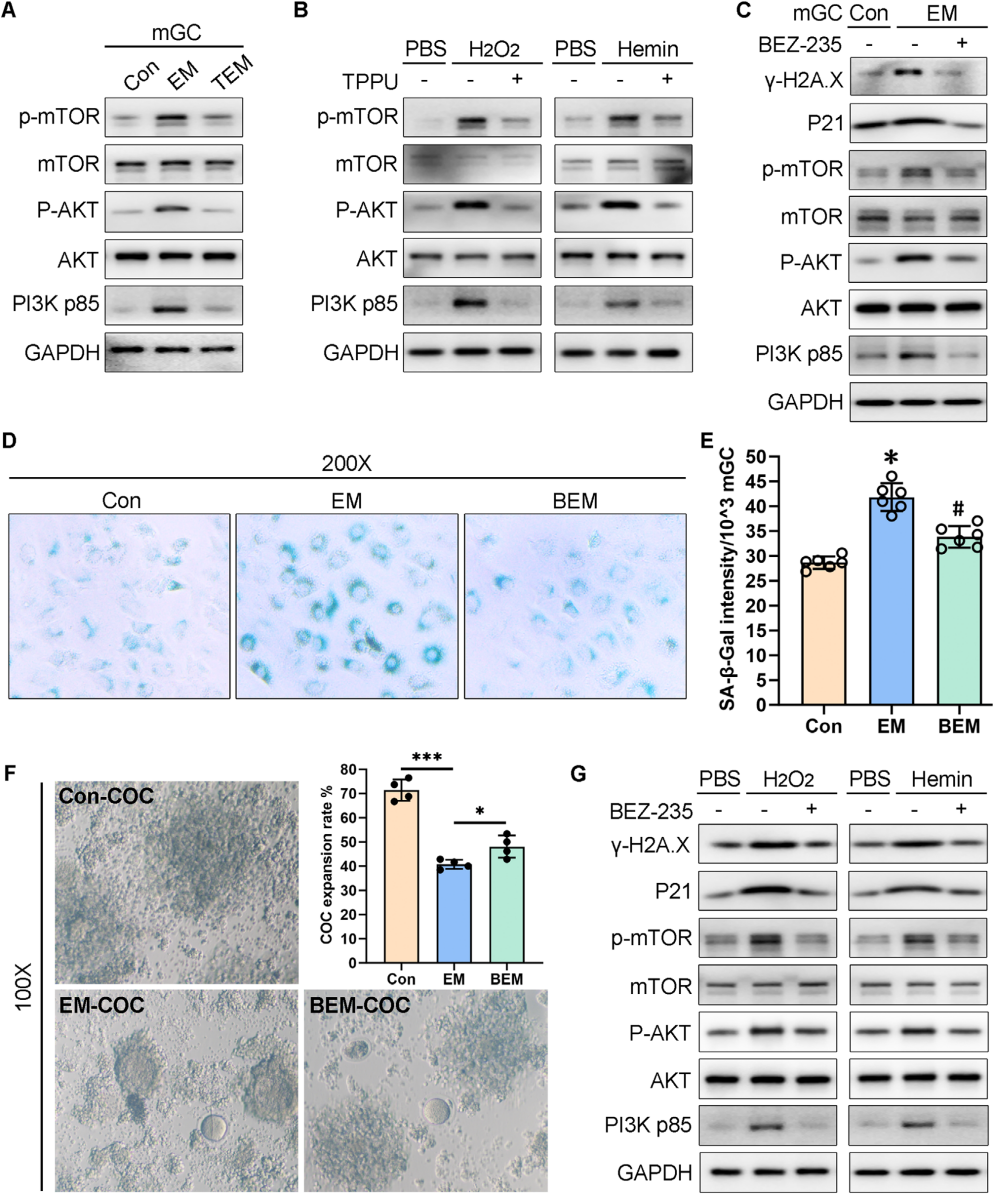
The PI3K‐AKT‐mTOR inhibitor BEZ‐235 rescued the defective cumulus‐oocyte complex (COC) expansion in endometriosis (EM) and alleviated mouse granulosa cell (mGC) senescence in vitro and in vivo. A) Western blot analysis of the indicated proteins in freshly extracted mGCs (*n* = 3 for Con group, *n* = 3 for EM group, *n* = 3 for TEM group, the same samples as described in Figure 7C). B) Western blot analysis of the indicated proteins in mGCs after 20 µm TPPU pre‐treatment for 24 h followed by H_2_O_2_ or hemin treatment for 48 h. mGCs were extracted from immature ICR mice. C) Western blot analysis of the indicated proteins in freshly extracted mGCs (n = 3 for Con group, *n* = 3 for EM group, *n* = 3 for BEM group; the BEM group received intragastric administration of 20 mg kg^−1^ BEZ‐235 daily for two weeks). D) SA‐β‐gal staining assay of freshly extracted mGCs (*n* = 3 for Con group, *n* = 3 for EM group, *n* = 3 for BEM group; the BEM group received intragastric administration of 20 mg kg^−1^ BEZ‐235 daily for two weeks). Magnification, 200×. (E) SA‐β‐gal quantitative assay of freshly extracted mGCs (*n* = 6 for Con group, *n* = 6 for EM group, *n* = 6 for BEM group). Unpaired *t*‐test, ^*^ *P* < 0.05 (EM vs Con), #*P* < 0.05 (BEM vs EM). F) In vitro COC expansion assay results. The defective EM‐COC expansion was partially rescued by BEZ‐235 treatment. The images shown in the left panel were acquired 12 h after COC culture in vitro. Magnification, 100×. The right panel shows the statistical analysis of the average COC expansion rates from four Con mice, four EM mice, and four BEM mice. Unpaired *t*‐test, ^***^ *P* < 0.001. G) Western blot analysis of the indicated proteins in mGCs after 200 nm BEZ‐235 pre‐treatment for 24 h followed by H_2_O_2_ or hemin treatment for 48 h. mGCs were extracted from immature ICR mice.

Western blot analysis showed that the PI3K p85, p‐AKT, and p‐mTOR protein expression levels were increased in mGCs from the EM group compared with those in mGCs from the control group (**Figure** **10**A). The mGCs from the TEM group showed suppressed PI3K p85, p‐AKT, and p‐mTOR protein expression patterns relative to those from the EM group (Figure 10A). TPPU pre‐treatment before oxidative stress induction reversed this ROS‐induced over‐activation of the PI3K/AKT/mTOR signaling pathway in mGCs (Figure 10B). Compared with the EM mice, BEZ‐235 administration in vivo for two weeks not only suppressed the over‐activation of PI3K p85, p‐AKT, and p‐mTOR, but also reversed the aging marker protein expression patterns (Figure 10C). The SA‐β‐gal staining intensity in mGCs was clearly decreased in the BEZ‐235 administration group (BEM group) compared with the EM group (Figure 10D). SA‐β‐gal quantitative assays also revealed decreased SA‐β‐gal activity in the BEM group compared with the EM group (Figure 10E). COC expansion was impaired in the EM group relative to the control group (Figure 10F), while BEZ‐235 gavage administration significantly improved the COC expansion rate in the BEM group compared with the EM group (Figure 10F). Additionally, H_2_O_2_ or hemin treatment significantly activated PI3K p85, p‐AKT, p‐mTOR, P21, and γ‐H2A.X protein expression in mGCs, while BEZ‐235 pre‐treatment before oxidative stress induction partially reversed this over‐activation of the PI3K/AKT/mTOR signaling pathway and ROS‐induced cell senescence (Figure 10G). These data suggested a vital role for BEZ‐235 in alleviating mGC senescence in vivo and in vitro. This function may be achieved by suppressing PI3K/AKT/mTOR signaling pathway over‐activation under oxidative stress conditions.

## 3 Discussion and Conclusion

Ovarian GC damage caused by excessive oxidative stress is an important contributor to EM‐associated infertility. Excessive oxidative stress can cause various types of damage to GCs, such as cellular senescence, ovulation disorders, and lipid metabolism disturbances.^[^ ^2^, ^8^, ^9^ ^]^ We observed abnormal oxidized lipid levels in the FF and ovarian GCs of women with EM‐associated infertility, with 14, 15‐EET displaying the most significant decrease. Moreover, the EET decrease was closely associated with poor IVF outcomes. Lower EET levels can lead to a reduced ovarian GC antioxidant capacity, decreased ATP production, lower MMP, and increased cell senescence. However, the addition of 14, 15‐EET or inhibiting EPHX2 could partially reverse these abnormalities. Our results indicate that the decreased 14, 15‐EET levels in EM follicles are caused by increased expression of soluble epoxide hydrolase EPHX2 in ovarian GCs. The increased EPHX2 expression levels in EM‐GCs may be from lower EZH2/H3K27Me3‐dependent histone methylation caused by excessive oxidative stress. Interestingly, we identified a positive feedback loop involving ROS‐EZH2/H3K27Me3‐EPHX2‐EET. ROS accumulation reduces H3K27Me3 histone methylation of *Ephx2*, leading to increased EPHX2 expression levels and enzymatic activity. The resulting decreased EETs levels further exacerbate the ROS accumulation, which forms a vicious cycle that aggravates ovarian GC senescence in women with EM and ultimately leads to poor IVF outcomes and infertility. Under non‐oxidative stress conditions, EPHX2 knockout does not elevate EZH2/H3K27Me3 (Figure 5F; Figure S5F, Supporting Information), with EPHX2 overexpression also failing to generate obvious senescence (Figure S5G, Supporting Information). Thus 14, 15‐EET‐mediated EZH2 upregulation requires ROS attenuation rather than direct epigenetic modulation. Crucially, this vicious cycle is disease context‐dependent.

Oxidative stress damage plays a crucial role in EM‐associated infertility, but the specific molecules and mechanisms involved have not been fully understood. Our study for the first time elucidated the oxidative lipid patterns in EM‐associated infertility and identified the anti‐senescence function of 14, 15‐EET. Furthermore, we proposed a potential explanation for the decreased 14, 15‐EET levels and a mechanism by which this change can lead to EM‐associated infertility. Our data using an EM mouse model suggested that oral administration of TPPU could inhibit EPHX2 enzymatic activity, upregulate EET levels, alleviate ovarian GC senescence, reduce ROS levels in oocytes, improve COC expansion, and improve EM mouse fertility. However, as described in Figure 7B, it remains premature to confirm that 14, 15‐EET is the primary EET responsible for GC senescence and EM‐associated infertility. In Alzheimer's disease,^[^ ^24^ ^]^ specific hepatic *Ephx2* knockdown selectively elevated plasma 14, 15‐EET levels without significantly altering the concentrations of the other three EETs (5, 6‐EET, 8, 9‐EET, and 11, 12‐EET). However, our results revealed that TPPU treatment in EM mice could induce upregulation of all four EETs in ovarian GCs (Figure 7B, TEM vs EM). This discrepancy in EET regulation patterns between the hepatic and ovarian systems may be from tissue‐specific epoxide hydrolase functions. While intracerebral injection of 14, 15‐EET has been studied in ischemic stroke mice,^[^ ^25^ ^]^ administering 14, 15‐EET directly into ovarian follicles is highly impractical. This is primarily because of two factors: first, the ovary contains multiple follicles at varying developmental stages and sizes; second, the small ovarian volume makes in vivo intra‐ovarian microinjection technically unfeasible. Future advancements are anticipated to overcome this technical limitation, enabling targeted delivery of 14, 15‐EET to ameliorate GC senescence and improve ART outcomes. However, 14, 15‐EET was the most significantly decreased EET among these four EET isomers in EM‐FF, with a nearly three‐fold difference (Figure 1C). 14, 15‐EET is also the most versatile EET in biological functions. Therefore, we believe that the anti‐aging effects and application prospects of 14, 15‐EET warrant specific attention.

To our knowledge, this is the first discussion of EPHX2 in the context of female infertility. Indirubin‐3′‐oxime mitigates heat‐induced male infertility in Drosophila melanogaster through EPHX2 inhibition,^[^ ^26^ ^]^ and the EPHX2 rs1042064 polymorphism demonstrates a significant association with reduced oligozoospermia and asthenospermia risks in Han Chinese populations.^[^ ^27^ ^]^ Our mechanistic studies and animal experimental results provide a theoretical basis for the potential future clinical use of this EPHX2 enzyme inhibitor for improving fertility in EM patients, but the prospects for clinical application still require significant progress before realization. Despite the existence of numerous soluble epoxide hydrolase inhibitors, including synthetic compounds such as TPPU, AUDA, EC5026, and G1,^[^ ^28^ ^]^ along with natural products like oleanane‐type triterpenoids,^[^ ^29^ ^]^ no inhibitor targeting human soluble epoxide hydrolase has received regulatory approval after clinical trials.^[^ ^30^ ^]^ These EPHX2 inhibitors elevate EET levels, alleviating inflammation, oxidative stress, endoplasmic reticulum stress, and fibrosis, thereby inhibiting the progression of neuropsychiatric disorders, inflammatory diseases, renal diseases, and chronic pain.^[^ ^31^, ^32^, ^33^ ^]^ However, systematic investigations into their reproductive toxicity remain inadequate. A critical challenge persists in developing inhibitors that selectively target EPHX2 in ovarian granulosa cells without affecting this enzyme in other organs.

Although EPHX2 upregulation is widely recognized as the primary driver of EET depletion, the role of upstream synthases, such as CYP3A5, remain to be fully elucidated. In various organs and cell types, arachidonic acid (AA) is metabolized into EETs by distinct cytochrome P450 (CYP) monooxygenases. To date, no published studies have specifically identified which CYP enzyme catalyzes the conversion of AA to EETs in human ovarian granulosa cells. Furthermore, recent evidence suggests that cytochrome P450 enzymes maintain normal expression and function in patients with endometriosis (EM).^[^ ^34^ ^]^ Based on our prior transcriptomic data,^[^ ^2^ ^]^ only a limited number of CYP monooxygenases were detected, among which CYP3A5 may serve as the principal enzyme responsible for EET biosynthesis in human GCs. Despite the absence of aberrant changes in upstream components such as AA (Figure S1, Supporting Information) or the CYP enzymes involved in EET synthesis (Figure S5B, Supporting Information), contributions from alternative epoxygenases, such as CYP2J2, cannot be fully ruled out and may influence EET production. Nevertheless, our data robustly demonstrate that elevated EPHX2 expression is the predominant mechanism for EET depletion in EM, as EPHX2 inhibition via TPPU or genetic knockdown universally restored 14, 15‐EET levels (Figures 5, 6, and 7B) and reversed cellular senescence. Future studies should explore whether compensatory CYP isoforms or post‐translational modifications contribute to EET dynamics. Our research in EM‐associated infertility and ovarian GCs also indicated that oxidative stress‐suppressed histone methylation may be involved in the observed upregulated EPHX2 expression patterns and decreased EET levels. In mice with ovarian GC‐specific *Ezh2* KO, we validated the regulatory relationship between histone methylation modifications and EPHX2. This also indicated that knocking out histone methyltransferase EZH2 not only can cause ovulation disorders, but also can interfere with follicle development.^[^ ^9^ ^]^ Our investigations of EPHX2 and 14, 15‐EET have deepened our understanding of oxidative stress‐induced infertility and provide new insights for developing antioxidant and anti‐senescence treatment methods in infertile women with EM.

Mechanistic studies have revealed that EPHX2 inhibition and 14, 15‐EET addition can reverse ovarian GC senescence and improve fertility by inhibiting PI3K/AKT/mTOR signaling pathway over‐activation in EM patients. Previous work has indicated that PI3K‐AKT‐mTOR signaling pathway activation is an important cause of cellular senescence.^[^ ^35^, ^36^, ^37^, ^38^, ^39^ ^]^ In many age‐related diseases, metformin, plant‐derived secondary metabolites (such as tea polyphenols and curcumin), and autophagy activators can alleviate cellular senescence and improve disease prognosis by inhibiting the PI3K‐AKT‐mTOR signaling pathway.^[^ ^40^, ^41^, ^42^ ^]^ In addition, high PI3K‐AKT‐mTOR signaling can result in excessive activation of primordial follicles, leading to premature ovarian failure.^[^ ^43^ ^]^ In contrast, inhibiting the PI3K‐AKT‐mTOR signaling pathway keeps the primordial follicles maintained in a dormant state in the ovarian follicle pool, while also inhibiting GC apoptosis and reducing the atresia of developing follicles.^[^ ^44^ ^]^ Our current study is the first to demonstrate that excessive activation of the PI3K‐AKT‐mTOR signaling pathway in GCs is involved in the pathogenesis of EM‐associated infertility, and suppressing excessive PI3K‐AKT‐mTOR signaling can alleviate GC senescence and improve COC expansion.

Activated PI3K‐AKT‐mTOR signaling can promote cell survival, inhibit cell apoptosis, and lead to the development and progression of various cancers.^[^ ^45^ ^]^ Other studies have reported that the PI3K‐AKT‐mTOR signaling pathway is overactivated in ectopic cysts from EM patients and plays an important role in cyst formation.^[^ ^46^, ^47^ ^]^ The broad‐spectrum PI3K inhibitor LY294002, broad‐spectrum AKT inhibitor MK2206, mTOR1 inhibitor rapamycin, and PI3K‐mTORC1/mTORC2 dual inhibitor BEZ‐235 can promote ectopic endometrial stromal cell apoptosis, reduce ectopic lesion neovascularization, alleviate pain, and inhibit EM development.^[^ ^48^, ^49^ ^]^ However, in our study, oral administration of BEZ‐235 in EM mice did not lead to a significant reduction in ectopic cyst volume, which may be stem from tissue‐specific bioavailability barriers, administration time, drug concentration and distinct pathogenic mechanisms between ovarian granulosa cell and endometriotic cells. Significant differences in the responses of ectopic lesions and ovarian granulosa cells to BEZ‐235 are biologically plausible, given their distinct cellular compositions, tissue environments, and molecular mechanisms. Although BEZ‐235 alleviates granulosa cell senescence by inhibiting the PI3K/AKT/mTOR pathway (Figure 10C), this pathway's role in ectopic lesions may differ from its function within the ovarian microenvironment. PI3K inhibitors (e.g., LY294002, BEZ‐235) directly target endometrial stromal cells to induce apoptosis and reduce lesions—a goal potentially compromised by inadequate drug concentration in ectopic sites following oral administration. In addition, most lesion‐reduction studies employ prophylactic regimens (pre‐ or immediate post‐transplantation), while BEZ‐235 treatment initiated at postoperative day 14 in our study (Figure S6A, Supporting Information), when ectopic cysts develop fully by transplantation. Furthermore, the non‐toxic BEZ‐235 dose (20 mg kg^−1^) likely fails to disrupt angiogenesis or ectopic endometrial survival sufficiently. Moreover, ectopic lesion persistence involves synergistic pathways (e.g., HIF‐1α, VEGF, miR‐210‐3p),^[^ ^50^, ^51^, ^52^ ^]^ suggesting that isolated PI3K/AKT/mTOR inhibition may inadequately suppress its growth.

In recent years, scholars have focused on the potential of antioxidant and anti‐aging treatments to improve IVF outcomes in EM patients. Common antioxidants, such as vitamin E, coenzyme Q10, and melatonin, have been used in clinical practice, but their effectiveness is generally limited.^[^ ^53^ ^]^ Our results here provide a theoretical basis for both in vivo and in vitro antioxidant and anti‐aging treatments, suggesting that pre‐ovulation oral administration of EPHX2 enzyme inhibitors or the addition of 14, 15‐EET during the IVF process could be promising adjunct therapeutic approaches in the future. In addition, oxidative stress damage does not only occur in women with EM‐associated infertility, but also in women with polycystic ovary syndrome, premature ovarian failure, and advanced maternal age.^[^ ^54^, ^55^ ^]^ Additional research is needed to understand if EPHX2 and EETs also play roles in these diseases.

## 4 Experimental Section

### Study Approval

The ethics committee of Sir Run Run Shaw Hospital, Zhejiang University approved and monitored these studies. The approval number for human tissues collection is 20240215–225, and the approval number for animal studies is SRRSH202402285. Informed consent was obtained from all participants prior to their involvement in the study. The detailed approval information is described in the Supporting Information.

### Controlled Ovarian Stimulation, Human FF and GC Collection, and Cell Culture

We enrolled 137 patients with both laparoscopic and histological diagnosis of EM (ovarian EM, deep infiltrating EM, or both) and 101 controls with tubal infertility at the Sir Run Shaw Hospital of Zhejiang University School of Medicine. The detailed inclusion and exclusion criteria are described in the Supporting Information.

Human FF was collected at the time of oocyte retrieval only from leading follicles with a diameter > 18 mm. Human GCs were collected by gently cutting the cumulus layer of each leading oocyte, washing it twice in phosphate‐buffered saline (PBS), and centrifuging it at 800×g for 5 min at 4 °C. Detailed procedures are presented in the Supporting Information. The demographics, clinical characteristics, and ART outcomes of all recruited patients are listed in Table S1 (Supporting Information).

The detailed procedures used for mice ovulation induction, cumulus‐oocyte complex (COC) collection, and mouse GC (mGC) harvest and culture are described in the Supporting Information.

### Targeted Liquid Chromatography Tandem Mass Spectrometry (LC‐MS/MS)

Human FF, human PF, human GCs, and mGCs were collected for LC‐MS/MS (Wuhan Matwell Biotechnology Co., Ltd., Wuhan, China). The detailed 141 oxidized lipids from the Lipid Oxidation Database V4.0 are listed in Table S2 (Supporting Information). The detailed LC‐MS/MS procedures are presented in the Supporting Information.

### Antioxidant Capacity Assay

The cell total antioxidant capacity was detected using the Total Antioxidant Capacity Assay Kit with a Rapid ABTS method from Beyotime Biotechnology (S0121, Beyotime Biotechnology, Shanghai, China). In brief, the cells were collected in 200 µL cold PBS and sonicated to fully break and release the antioxidants. The samples were then centrifuged at 12000×g for 5 min at 4 °C and the supernatants were collected for subsequent analysis.

### Detection of Cellular ATP Levels

The ATP levels in mGC lysates were measured using a luminometer (Synergy H4, BioTek Instruments, Inc., Vermont, USA) according to the manufacturer's instructions (S0027, Beyotime Biotechnology). The total protein of each mGC sample was quantitated to normalize the ATP levels. The detailed ATP and protein quantification procedures are presented in the Supporting Information.

### Immunofluorescence (IF) Analysis of Mitochondrial Membrane Potential (MMP) andγ‐H2A.X

The mGC MMP was detected using the JC‐1 Assay Kit (C2006, Beyotime Biotechnology) combined with fluorescence microscopy according to the manufacturer's instructions. IF assays were performed in mGCs using a standard staining procedure, as described in the Supporting Information. The primary antibodies used for IF assays are listed in Table S4 (Supporting Information).

### SA‐β‐gal Staining Assay

SA‐β‐gal staining assays were performed according to the manufacturer's instructions (C0602, Beyotime Biotechnology). The mGCs were fixed and stained in X‐gal solution overnight at 37 °C. All cells were imaged and photographed using a microscope (IX70, Olympus, Japan).

### Cellular Senescence Quantitative Assay

An equal quantity of cells was used to measure SA‐β‐gal activity. Senescence quantitative assays were performed in mGCs using the 96‐well Cellular Senescence Assay Kit (CBA‐231, Cell Biolabs, Inc., San Diego, CA, USA) according to the manufacturer's instructions. The mGCs were lysed and the protein concentrations were measured using the Pierce BCA Protein Assay Kit (23 225, Thermo Fisher Scientific, Waltham, MA, USA) via a standard protocol.

### Enzyme‐Linked Immunosorbent Assay (ELISA)

ELISAs were used to evaluate the concentrations of 14, 15‐EET and the soluble advanced glycation end products receptor (sRAGE) isoform in mGC culture supernatants. The Mouse 14, 15‐EET Kit was purchased from Mlbio Biotech (YJ112055, Shanghai, China). The Mouse sRAGE ELISA Kit (EK2103‐96) was purchased from Multi Sciences Biotech (Hangzhou, China).

### RNA Isolation and Quantitative Reverse Transcriptase PCR (qRT‐PCR)

RNA isolation and qRT‐PCR were performed using the RNA‐Quick Purification Kit 189 (RN001, Shanghai Yishan Biotechnology Co., Ltd., Shanghai, China) following the manufacturer's instructions. The primer sequences are listed in Table S3 (Supporting Information).

### Western Blot Analysis

Western blot analysis was performed following standard protocols. Image J software (National Institutes of Health, Bethesda, MD, USA) was used to analyze the signal intensity of the protein bands. The primary antibodies used for western blot analysis are listed in Table S4 (Supporting Information). A goat anti‐rabbit horseradish peroxidase‐conjugated antibody (111‐035‐003, Jackson ImmunoResearch, West Grove, PA, USA) and goat anti‐mouse antibody (115‐035‐003, Jackson ImmunoResearch) were used as secondary antibodies.

To induce oxidative stress in mGCs, 100 µm H_2_O_2_ or 10 µm hemin was used.^[^ ^8^, ^17^ ^]^ Additionally, 100 nm 14, 15‐EET (HY‐113489, MedChemExpress, NJ, USA) was used in further work according to the preliminary experiments, as described in Figure S3 (Supporting Information). The in vitro working concentrations for TPPU (HY‐101294, MedChemExpress) and BEZ‐235 (HY‐50673, MedChemExpress) were 20 µm and 200 nm, respectively. Furthermore, 20 µm SC79 (SF2730, Beyotime Biotechnology) co‐culture for 24 h was used to reactivate p‐AKT in mGCs.^[^ ^56^, ^57^ ^]^ The concentrations used for gavage administration of TPPU and BEZ‐235 were 3  and 20 mg kg^−1^, respectively, based on the mouse body weight.

### Lentivirus Infection

The mGC isolation, purification, and culture procedures were performed according to the previously described protocol.^[^ ^9^ ^]^ The lentiviral vectors expressing short hairpin RNAs (shRNAs) targeting EZH2 (Sh‐EZH2) or EPHX2 (Sh‐EPHX2), and the lentiviral vector overexpressing EZH2 (LV‐EZH2) or EPHX2 (LV‐EPHX2), as well as their corresponding negative control lentiviruses (Sh‐NC or LV‐NC), were purchased from Shandong Weizhen Biotechnology Co., Ltd. (Jinan, China). Cells were seeded in 6‐well plates and infected with lentivirus at a multiplicity of infection of 10 for 48 h. The medium was changed after 24 h of culture. PCR or western blot analysis was used to confirm the infection efficiency.

### Chromatin Immunoprecipitation (ChIP) and Real‐Time qPCR

ChIP assays were performed using the Simple ChIP Enzymatic Chromatin IP Kit (#9003, Cell Signaling Technology (CST), Danvers, MA, USA) following the manufacturer's instructions. ChIP‐grade anti‐H3K27Me3 antibody (#9733, CST) and negative control Normal Rabbit IgG (#2729, CST) were used for IP (1 µg antibody per IP sample). The ChIP‐PCR primer sequences are listed in Table S3 (Supporting Information). The detailed procedures are described in the Supporting Information.

### Establishment of the EM Mouse Model and Drug Administration In Vivo

EM model mice were established by mouse‐mouse intraperitoneal implantation, as previously described.^[^ ^17^, ^58^, ^59^, ^60^ ^]^ The detailed information for establishing this EM mouse model and TPPU and BEZ‐235 administration procedures are included in the Supporting Information. A concise description of the animal experimental procedures is presented in Figure S6 (Supporting Information).

### Fertility Assessment

Ten‐week‐old EM mice (*n* = 7 for each group) in estrus were housed with eight‐week‐old fertile male mice (2:1). The following day after mating, vaginal plugs were checked to confirm successful mating and all female mice were used for recording births. The pup sizes were determined at 21 days after birth. The fertility test was conducted over 6 months, as previously described.^[^ ^17^ ^]^

### In Vitro COC Expansion Assay

COCs were collected from the ovaries of PMSG‐treated (44 to 46 h) mice. COCs were plated in 40 µL defined COC medium^[^ ^61^ ^]^ under the cover of mineral oil for 12 h. The detailed procedures are described in the Supporting Information.

### ROS Assay

The DCFH‐DA fluorescent probe was used for ROS detection (S0033S, Beyotime Biotechnology). In brief, mouse oocytes were suspended in 10 µm DCFH‐DA for 20 min at 37 °C in M2 medium. The oocytes were washed three times with PBS to remove any DCFH‐DA that did not enter. After probe loading, the ROS levels were detected using fluorescence microscopy.

### RNA Sequencing (RNA‐seq) and Bioinformatics Analyses

RNA‐seq analysis was conducted in two distinct experimental phases. First, to elucidate GC abnormalities in EM mice, EM mouse models were constructed and performed RNA‐seq analysis on freshly isolated mGCs obtained from the sham surgery group (Con‐mGC, *n* = 5) or EM group (EM‐mGC, *n* = 5) after ovulation induction. A total of 10 mice were used in this experiment, with five sham surgery mice and five EM model mice.

Second, freshly isolated mGCs from six EM model mice were randomly divided into two parts for in vitro culture: one part was cultured in DMEM/F12 with 5% fetal bovine serum (FBS) for 24 h, while the other part was additionally treated with 100 nm 14, 15‐EET for 24 h. A total of six EM mice were used in this experiment, with three paired biological replicates of mGCs collected for RNA‐seq analysis. The detailed procedures are described in the Supporting Information.

### Statistical Analysis

All experiments were repeated at least three biological times. SPSS version 19.0 (SPSS, Chicago, IL, USA) and GraphPad Prism 10 (Graph Pad Software, San Diego, CA, USA) were used for statistical analysis. Statistical comparisons between two groups were performed using the unpaired Student's t‐test or Mann‐Whitney U test after examining data normality. Comparisons of continuous variables among groups were conducted by one‐way ANOVA followed by least significant difference tests. Pearson correlation analysis was used to estimate the correlations between different clinical in vitro fertilization (IVF) outcomes and the EET levels in FF.

### Materials and Methods

Details of the experimental protocols, including those for the cell treatments, PCR, western blot analysis, immunohistochemistry, ChIP, IF, ELISA, LC‐MS/MS, conditional KO mouse, and EM mouse model development, are provided in the Supporting Information. The protein quantification results are summarized in Figure S7 (Supporting Information).

## Conflict of Interest

The authors declare no conflict of interest.

## Author Contributions

X.L. and W.G. contributed equally and share first authorship. X.L. and W.G. performed the experiments. X.L., M.L., Y.Z., F.Z., N.L., X.J., C.L., and D.H. collected the clinical samples. X.L. and Y.D. analyzed the data, made the figures, and drafted the manuscript. Y.D., X.T. and S.Z. critically reviewed the manuscript. X.L. and S.Z. designed and conceived the project. Y.D. and S.Z. supervised the study and were responsible for critical revision of the manuscript for important intellectual content.

## Supporting information



Supporting Information

## Data Availability

The sequencing data have been deposited into the GEO database (GSE304488) and will become publicly accessible following the publication. The original contributions presented in the study are included in the article or supplemental materials. Further inquiries can be directed to the corresponding author.

## References

[1] T. Tanbo , P. Fedorcsak , Acta. Obstet. Gyn. Scan. 2017, 96, 659. 10.1111/aogs.13082 27998009

[2] X. Lin , Y. D. Dai , X. M. Tong , W. Z. Xu , Q. M. Huang , X. Y. Jin , C. Li , F. Zhou , H. J. Zhou , X. N. Lin , et al., Redox Biol. 2020, 30, 101431. 31972508 10.1016/j.redox.2020.101431 PMC6974790

[3] U. Leone Roberti Maggiore , V. Chiappa , M. Ceccaroni , G. Roviglione , L. Savelli , S. Ferrero , F. Raspagliesi , L. Spano Bascio , Best Pract. Res. Clin. Obstet. Gynaecol. 2024, 92, 102454. 38183767 10.1016/j.bpobgyn.2023.102454

[4] D. de Ziegler , B. Borghese , C. Chapron , Lancet 2010, 376, 730. 20801404 10.1016/S0140-6736(10)60490-4

[5] S. Hayashi , T. Nakamura , Y. Motooka , F. Ito , L. Jiang , S. Akatsuka , A. Iwase , H. Kajiyama , F. Kikkawa , S. Toyokuni , Redox Biol. 2020, 37, 101726. 32961443 10.1016/j.redox.2020.101726 PMC7509075

[6] V. Baena , M. Terasaki , Sci. Rep. 2019, 9, 1262. 30718581 10.1038/s41598-018-37766-2 PMC6362238

[7] M. G Da Broi , P. A. Navarro , Cell Tissue Res. 2016, 364, 1. 26685866 10.1007/s00441-015-2339-9

[8] Y. Dai , X. Lin , N. Liu , L. Shi , F. Zhuo , Q. Huang , W. Gu , F. Zhao , Y. Zhang , Y. Zhang , Y. Pan , S. Zhang , J. Pathol. 2023, 260, 248. 36992523 10.1002/path.6079

[9] X. Lin , X. Tong , Y. Zhang , W. Gu , Q. Huang , Y. Zhang , F. Zhuo , F. Zhao , X. Jin , C. Li , D. Huang , S. Zhang , Y. Dai , Endocrinology 2022, 164, bqac210. 36524678 10.1210/endocr/bqac210 PMC9825353

[10] M. Moqri , C. Herzog , J. R. Poganik , J. Justice , D. W. Belsky , A. Higgins‐Chen , A. Moskalev , G. Fuellen , A. A. Cohen , I. Bautmans , M. Widschwendter , J. Ding , A. Fleming , J. Mannick , J.‐D. J. Han , A. Zhavoronkov , N. Barzilai , M. Kaeberlein , S. Cummings , B. K. Kennedy , L. Ferrucci , S. Horvath , E. Verdin , A. B. Maier , M. P. Snyder , V. Sebastiano , V. N. Gladyshev , Cell 2023, 186, 3758. 37657418 10.1016/j.cell.2023.08.003 PMC11088934

[11] C. Lopez‐Otin , M. A. Blasco , L. Partridge , M. Serrano , G. Kroemer , Cell 2023, 186, 243. 36599349 10.1016/j.cell.2022.11.001

[12] B. Wang , Y. Wang , J. Zhang , C. Hu , J. Jiang , Y. Li , Z. Peng , Arch. Toxicol. 2023, 97, 1439. 37127681 10.1007/s00204-023-03476-6

[13] K. Tomita , Y. Takashi , Y. Ouchi , Y. Kuwahara , K. Igarashi , T. Nagasawa , H. Nabika , A. Kurimasa , M. Fukumoto , Y. Nishitani , T. Sato , Cancer Sci. 2019, 110, 2856. 31314163 10.1111/cas.14132 PMC6726706

[14] A. N. von Krusenstiern , R. N. Robson , N. Qian , B. Qiu , F. Hu , E. Reznik , N. Smith , F. Zandkarimi , V. M. Estes , M. Dupont , T. Hirschhorn , M. S. Shchepinov , W. Min , K. A. Woerpel , B. R. Stockwell , Nat. Chem. Biol. 2023, 19, 719. 36747055 10.1038/s41589-022-01249-3 PMC10238648

[15] H. Zhao , J. Tang , H. Chen , W. Gu , H. Geng , L. Wang , Y. Wang , Int. J. Mol. Sci. 2021, 14, 22. 10.3390/ijms22189660 PMC8471287 34575823

[16] D. Jonnalagadda , D. Wan , J. Chun , B. D. Hammock , Y. Kihara , Int. J. Mol. Sci. 2021, 22, 4650. 33925035 10.3390/ijms22094650 PMC8125305

[17] X. Lin , Y. Dai , X. Tong , W. Xu , Q. Huang , X. Jin , C. Li , F. Zhou , H. Zhou , X. Lin , D. Huang , S. Zhang , Redox Biol. 2020, 30, 101431. 31972508 10.1016/j.redox.2020.101431 PMC6974790

[18] J. Checa , J. M. Aran , J. Inflamm. Res. 2020, 13, 1057. 33293849 10.2147/JIR.S275595 PMC7719303

[19] C. Y. Zhang , W. J. Zhong , Y. B. Liu , J. X. Duan , N. Jiang , H. H. Yang , S. C. Ma , L. Jin , J. R. Hong , Y. Zhou , et al., Redox Biol. 2023, 63. 10.1016/j.redox.2023.102765 PMC10249012 37269686

[20] J. J. Shan , K. Hashimoto , Int. J. Mol. Sci. 2022, 23, 4951.35563342

[21] Q. Ren , M. Ma , T. Ishima , C. Morisseau , J. Yang , K. M. Wagner , J.‐C. Zhang , C. Yang , W. Yao , C. Dong , M. Han , B. D. Hammock , K. Hashimoto , P Natl Acad Sci USA 2016, 113, E1944. 10.1073/pnas.1601532113 PMC4822604 26976569

[22] A. I. Ostermann , J. Herbers , I. Willenberg , R. J. Chen , S. H. Hwang , R. Greite , C. Morisseau , F. Gueler , B. D. Hammock , N. H. Schebb , Prostag. Oth. Lipid. M. 2015, 121, 131. 10.1016/j.prostaglandins.2015.06.005 PMC4688279 26117215

[23] A. Ulu , S. Appt , C. Morisseau , S. Hwang , P. Jones , T. Rose , H. Dong , J. Lango , J. Yang , H. Tsai , C. Miyabe , C. Fortenbach , M. Adams , B. Hammock , Br. J. Pharmacol. 2012, 165, 1401. 21880036 10.1111/j.1476-5381.2011.01641.x PMC3372725

[24] Y. Wu , J.‐H. Dong , Y.‐F. Dai , M.‐Z. Zhu , M.‐Y. Wang , Y. Zhang , Y.‐D. Pan , X.‐R. Yuan , Z.‐X. Guo , C.‐X. Wang , Y.‐Q. Li , X.‐H. Zhu , Neuron 2023, 111, 2810. 10.1016/j.neuron.2023.06.002 37402372

[25] H. Geng , J. Tang , Z. Li , Y. Zhang , C. Ye , Y. Zhang , X. Li , Y. Li , Y. Wang , Y. Wang , et al., Stroke 2025, 56, 1883. 40235438 10.1161/STROKEAHA.124.049143

[26] N. V. Phong , H. S. Kim , Y. Zhao , E. Yeom , S. Y. Yang , J. Enzyme Inhib. Med. Chem. 2025, 40, 2447719. 39840826 10.1080/14756366.2024.2447719 PMC11755746

[27] Y. Qin , X. Han , Y. Peng , R. Shen , X. Guo , L. Cao , L. Song , J. Sha , Y. Xia , X. Wang , Gene 2012, 510, 171. 22986331 10.1016/j.gene.2012.09.016

[28] Y. Chen , L. Chen , H. Xu , R. Cao , C. Morisseau , M. Zhang , Y. Shi , B. D. Hammock , J. Wang , J. Zhuang , Z. Liu , G. Chen , J. Med. Chem. 2023, 66, 2979. 36689364 10.1021/acs.jmedchem.2c01996 PMC9974930

[29] W. Y. Zhao , X. Y. Zhang , M. R. Zhou , X. G. Tian , X. Lv , H. L. Zhang , S. Deng , B. J. Zhang , C. P. Sun , X. C. Ma , Int. J. Biol. Macromol. 2021, 183, 811. 33957203 10.1016/j.ijbiomac.2021.04.187

[30] M. Bzowka , K. Mitusinska , K. Hopko , A. Gora , Drug Discovery Today 2021, 26, 1914. 34082135 10.1016/j.drudis.2021.05.017

[31] J. Shan , K. Hashimoto , Int. J. Mol. Sci. 2022, 23, 4951.35563342

[32] J. Turnbull , V. Chapman , Curr. Opin. Pharmacol. 2024, 78, 102477. 39197248 10.1016/j.coph.2024.102477

[33] J. Gautheron , I. Jeru , Int. J. Mol. Sci. 2020, 22, 13. 33374956 10.3390/ijms22010013 PMC7792612

[34] E. Giacomini , L. Pagliardini , S. Minetto , M. Pinna , F. Kleeman , F. Bonesi , S. Makieva , V. Pavone , M. Reschini , E. Papaleo , M. Candiani , E. Somigliana , P. Viganò , J. Steroid Biochem. Mol. Biol. 2024, 237, 13106439. 10.1016/j.jsbmb.2023.106439 38048918

[35] R. Kumar , A. Sharma , A. Kumari , A. Gulati , Y. Padwad , R. Sharma , Biogerontology 2019, 20, 171. 30456590 10.1007/s10522-018-9785-1

[36] C. Y. Guan , D. Zhang , X. C. Sun , X. Ma , H. F. Xia , Stem Cells Int. 2024, 2024, 3100942. 39108701 10.1155/2024/3100942 PMC11303045

[37] J. H. Park , J. J. Kim , Y. S. Bae , Biochem. Biophys. Res. Commun. 2013, 433, 420. 23523798 10.1016/j.bbrc.2013.02.108

[38] G. Liu , X. Li , F. Yang , J. Qi , L. Shang , H. Zhang , S. Li , F. Xu , L. Li , H. Yu , et al., Aging Dis. 2023, 14, 1425. 37163424 10.14336/AD.2023.0121 PMC10389819

[39] G. Ai , M. Meng , J. Guo , C. Li , J. Zhu , L. Liu , B. Liu , W. Yang , X. Shao , Z. Cheng , L. Wang , Stem. Cell. Res. Ther. 2023, 14, 75. 37038203 10.1186/s13287-023-03297-5 PMC10088140

[40] Q. Chen , H. Zhang , Y. Yang , S. Zhang , J. Wang , D. Zhang , H. Yu , Int. J. Mol. Sci. 2022, 23, 6960.35805987

[41] S. Fakhri , A. Iranpanah , M. M. Gravandi , S. Z. Moradi , M. Ranjbari , M. B. Majnooni , J. Echeverría , Y. Qi , M. Wang , P. Liao , M. H. Farzaei , J. Xiao , Phytomedicine 2021, 91, 153664. 34391082 10.1016/j.phymed.2021.153664

[42] X. Fan , Y. He , G. Wu , H. Chen , X. Cheng , Y. Zhan , C. An , T. Chen , X. Wang , Biochim Biophys Acta Mol Cell Res. 2023, 1870, 119411. 36521686 10.1016/j.bbamcr.2022.119411

[43] K. Papageorgiou , E. Mastora , A. Zikopoulos , M. E. Grigoriou , I. Georgiou , T. M. Michaelidis , Front. Endocrinol 2021, 12, 702446. 10.3389/fendo.2021.702446 PMC8334720 34367070

[44] A. J. Hsueh , K. Kawamura , Y. Cheng , B. C. Fauser , Endocr Rev. 2015, 36, 1. 25202833 10.1210/er.2014-1020 PMC4309737

[45] A. S. Alzahrani , Semin. Cancer Biol. 2019, 59, 125. 31323288 10.1016/j.semcancer.2019.07.009

[46] S. W. Hung , R. Zhang , Z. Tan , J. P. W. Chung , T. Zhang , C. C. Wang , Med. Res. Rev. 2021, 41, 2489. 33948974 10.1002/med.21802 PMC8252000

[47] M. Zhang , T. Xu , D. Tong , S. Li , X. Yu , B. Liu , L. Jiang , K. Liu , Biomed. Pharmacother. 2023, 164, 114909. 37210898 10.1016/j.biopha.2023.114909

[48] F. Barra , L. Ferro Desideri , S. Ferrero , Br. J. Pharmacol. 2018, 175, 3626. 29984446 10.1111/bph.14391 PMC6086976

[49] Y. Liu , X. Qin , X. Lu , J. Jiang , Can. J. Physiol. Pharmacol. 2019, 97, 963. 31461309 10.1139/cjpp-2019-0156

[50] X. Lin , Y. Dai , W. Xu , L. Shi , X. Jin , C. Li , F. Zhou , Y. Pan , Y. Zhang , X. Lin , S. Zhang , Endocrinology 2018, 159, 1630. 29438550 10.1210/en.2017-03227

[51] Y. D. Dai , X. Lin , W. Z. Xu , X. N. Lin , Q. M. Huang , L. B. Shi , Y. B. Pan , Y. L. Zhang , Y. S. Zhu , C. Li , et al., Cell Death Dis. 2019, 10. 10.1038/s41419-019-1395-6 PMC6374490 30760709

[52] J. Zheng , Y. Dai , X. Lin , Q. Huang , L. Shi , X. Jin , N. Liu , F. Zhou , S. Zhang , Mol. Med. Rep. 2021, 24, 637. 34278456 10.3892/mmr.2021.12276 PMC8281285

[53] M. G. Showell , R. Mackenzie‐Proctor , V. Jordan , R. J. Hart , Cochrane Database Syst Rev. 2020, 8, CD007807. 32851663 10.1002/14651858.CD007807.pub4 PMC8094745

[54] L. Wang , J. Tang , L. Wang , F. Tan , H. Song , J. Zhou , F. Li , J. Cell. Physiol. 2021, 236, 7966. 34121193 10.1002/jcp.30468

[55] A. Agarwal , A. Aponte‐Mellado , B. J. Premkumar , A. Shaman , S. Gupta , Reprod. Biol. Endocrinol. 2012, 10, 49. 22748101 10.1186/1477-7827-10-49 PMC3527168

[56] E. Y. So , T. Ouchi , BMC Cancer 2014, 14, 548. 25070371 10.1186/1471-2407-14-548 PMC4129107

[57] H. Jo , S. Mondal , D. Tan , E. Nagata , S. Takizawa , A. K. Sharma , Q. Hou , K. Shanmugasundaram , A. Prasad , J. K. Tung , A. O. Tejeda , H. Man , A. C. Rigby , H. R. Luo , Proc. Natl. Acad. Sci. USA 2012, 109, 10581. 22689977 10.1073/pnas.1202810109 PMC3387065

[58] C. E. Moon , M. C. Bertero , T. E. Curry , S. N. London , K. N. Muse , K. L. Sharpe , M. W. Vernon , Am. J. Obstet. Gynecol. 1993, 169, 676. 8372879 10.1016/0002-9378(93)90642-v

[59] Y. Dai , X. Lin , W. Xu , X. Lin , Q. Huang , L. Shi , Y. Pan , Y. Zhang , Y. Zhu , C. Li , L. Liu , S. Zhang , Cell Death Dis. 2019, 10, 144. 30760709 10.1038/s41419-019-1395-6 PMC6374490

[60] K. E. Pelch , K. L. Sharpe‐Timms , S. C. Nagel , J. Visualized Exp. 2012, 59, 3396. 10.3791/3396 PMC3339870 22257948

[61] H. Y. Fan , A. O'Connor , M. Shitanaka , M. Shimada , Z. Liu , J. S. Richards , Mol. Endocrinol. 2010, 24, 1529. 20610534 10.1210/me.2010-0141 PMC2940463

