# Supplementary material for: EETs Reduction Contributes to Granulosa Cell Senescence and Endometriosis‐Associated Infertility via the PI3K/AKT/mTOR Signaling Pathway
Source: Adv Sci (Weinh). 2025 Aug 19;12(42):e05656. doi: 10.1002/advs.202505656 (PMC12622488; doi:10.1002/advs.202505656)
Supplement: Supplementary file 1 — Supporting Information [file ADVS-12-e05656-s001.docx]

***Supplementary Information***

1. ***Study approval***

This study was initiated on 1 December 2022 and terminated on 30 January 2025. The ethics committee of Sir Run Run Shaw Hospital, Zhejiang University approved and monitored this study. Written informed consent was obtained from all patients before sample collection. The mice were bred in accordance with the National Institutes of Health Guide for the Care and Use of Laboratory Animals. The approval number for human tissues collection is 20240215-225, and the approval number for animal studies is SRRSH202402285.

1. ***Inclusion and exclusion criteria of infertility patients***

Infertility was defined as the failure to establish a clinical pregnancy after 12 months of regular, unprotected sexual intercourse, or from an impairment of a person’s capacity to reproduce either as an individual or with his/her partner [[1](#_ENREF_1)]. All patients had just one previous completed surgical excision of endometriotic lesions or salpingectomy, and this was their first request for assisted reproductive therapy. No patients received hormones within the 12-month period before the study commenced.

Endometriosis is defined as the presence of endometrium-like epithelium and/or stroma outside the endometrium and myometrium, usually with an associated inflammatory process [[2](#_ENREF_2)]. We included patients with ovarian endometriosis or deep infiltrating endometriosis or both in our research. Patients with endometriosis were graded as I–IV following the revised American Fertility Society classification system, and all patients received only one operation by video laparoscopy 1 year before the assisted reproduction treatment. All patients with endometriosis included in our study were confirmed by peritoneoscope surgery, followed by histological proof.

According to our previous published data, no differences were found in EZH2 and H3K27Me3 expression between stage I-II and III-IV of endometriosis [[3](#_ENREF_3)]. For EM patients, they sought assisted reproductive therapy in our center mainly because they were still infertile 1 year after the surgery, and they feel a recurrence of symptoms such as periodic lower abdominal pain, pelvic pain, dyspareunia, or an elevated serum CA125 level after the surgery. All control patients sought assisted reproductive therapy in our center mainly because they were infertile 1 year after unilateral or bilateral salpingectomy surgery.

Inclusion criteria were as follows: (1) a history of infertility >1 year; (2) age 20–38 years; (3) basal serum follicle-stimulating hormone levels < 8 IU/L and basal serum luteinizing hormone (LH) levels < 10 IU/L before controlled ovarian hyper-stimulation; and (4) basic antral follicle count (AFC) > 6. Exclusion criteria included the following: (1) polycystic ovary syndrome or pure high androgen levels, premature ovarian failure, uterus diseases (e.g., uterine fibroids, Ashman, adenomyosis, or endometrial polyp), diabetes, hypertension, thyroid diseases, and other serious liver, kidney, heart, or blood diseases; (2) any other untreated or insufficiently corrected endocrinopathies or immunopathy; and (3) smoking, alcoholism, or drug addiction.

1. ***Assisted reproductive treatments in patients***

Ovulation induction was initiated by gonadotropin on the second day of menstruation or pretreated with standard prolonged action gonadotropin-releasing hormone agonist (leuprolide acetate) or after ovulation according to the individual circumstances of each patient [[4-6](#_ENREF_4)]. This study includes various ovulation induction approaches, such as agonist schemes, antagonist schemes, PPOS, and micro-stimulation schemes. Fresh or frozen embryo transfer was chosen after evaluating embryo quality and endometrial receptivity. Owing to individual differences, the dose of gonadotropin may differ in each patient. However, no difference was found in the average dose of gonadotropin between control and endometriosis groups [[7](#_ENREF_7), [8](#_ENREF_8)]. If standard prolonged action gonadotropin-releasing hormone agonist protocol was used, downregulation was achieved when serum LH < 3 IU/L, serum estradiol < 50 ng/L, and endometrial thickness < 5 mm. Patients then received gonadotropin with an empirical protocol based on age, body mass index, AFC, follicular diameter, and hormone status.

Controlled ovarian stimulation was accomplished until at least two follicles reached a diameter of 18 mm and then follicular maturation was triggered via an injection of hCG (5000–10,000 IU according to body mass index, estrogen levels and follicular diameter). Transvaginal ultrasound-guided oocyte retrieval was performed 34–36 h later, followed by IVF or ICSI. ICSI was performed only when the partner had confirmed severe male infertility with a sperm count < 5 × 10^6^ per mL and sperm motility < 10% or normal morphology < 1%.

1. ***Definition of clinical parameters***

Oocytes were considered normal fertilization when two pronuclei were observed at 16–18 h following IVF or ICSI. Embryos of good morphological quality were identified on day 3 as those having at least six regular blastomeres and less than 20% anucleate fragments. Embryo quality and grading was assessed by the percentage of fragment [[9](#_ENREF_9)]. Grade A, B, C, and D embryos were defined as having fragment percentages less than 10%, between 10%–20%, between 20%–50%, and greater than 50%, respectively. Grade A and B embryos were considered good quality embryos. The number of transferred blastaea was less than two and the number of transferred D3 embryos was less than three. A positive pregnancy was considered when serum hCG levels were ≥ 20 IU/L after embryo transfer for 12 days. Clinical pregnancy or intrauterine pregnancy was confirmed when the fetal bud appeared in intrauterine with a positive heartbeat 35 days after embryo transfer. Biochemical pregnancy was indicated as positive serum hCG at 14 days, but negative ultrasound at 35 days after embryo transfer. The detailed clinical characteristics of participants were listed in *Supplementary Table 1*.

1. ***Human sample collection and preservation***

Only clear FF without blood was collected for experiments. Each FF sample was centrifuged for 20 min at 3000 × g (at 4ºC) immediately, and supernatants were then stored at -80ºC until further analysis. Ovary cumulus granulosa cells (GCs) stripped from leading oocytes were washed twice in ice-cold PBS and centrifuged at 800 × g (at 4ºC) for 5 min. Lysis buffer was immediately added to GCs to extract RNA or protein, and cell samples were stored at **−**80ºC until further analysis.

It is important to obtain enough human GCs to extract RNA and quantify target protein expression, but excessive stripping of GCs from the oocyte is not allowed and unacceptable. To preserve the original characteristics of primary human GCs and minimize differences caused by sample selection, we did not mix primary GCs samples from different patients and normalized cell numbers before qRT-PCR, western blot, RNA-sequencing, and other experiments. For qRT-PCR, 1 × 10^6^ GCs per sample is needed; and for western blot, 5 × 10^6^ GCs per sample is needed.

1. ***Mouse sample collection and treatment***

Female ICR mice were super-ovulated using an intraperitoneal injection of 4-5IU pregnant mare serum gonadotropin (PMSG) according to mouse weight, then the ovaries were collected after 44 to 46 hours. Generally, the ovaries were placed in L-15 medium and the cumulus-oocyte complexes (COCs) were collected from well-developed graafian follicles via 26.5-gauge needles. Mouse GCs (mGCs) were harvested from PMSG-primed immature female mice (21 days old) or endometriosis mouse model as previously described [[10](#_ENREF_10)]. These mGCs were cultured in DMEM/F-12 (Jinuo Biomedical Technology Company, Hangzhou, China) supplemented with 5% fetal bovine serum (FBS) and incubated at 37°C in 5% CO_2_. Mature oocytes were obtained directly from the fallopian tube after intraperitoneal injection of human chorionic gonadotropin (HCG) for 14 to 16 hours.

1. ***Liquid chromatography tandem mass spectrometry (LC-MS/MS)***
   1. *Chemicals and reagents*

All eicosanoids and deuterated internal standards were purchased from Cayman Chemical. HPLC-grade acetonitrile (ACN) and methanol (MeOH) were purchased from Merck (Darmstadt, Germany). MilliQ water (Millipore, Bradford, USA) was used in all experiments. Acetic acid was purchased from Sigma-Aldrich. CNW Poly-Sery MAX SPE cartridges were from ANPEL Co. (Shanghai, PRC). The stock solutions of standards were prepared at the concentration of 5 μg/mL in MeOH. All stock solutions were stored at -80°C. The stock solutions were diluted with MeOH to working solutions before analysis.

- 1. *Sample preparation and extraction*

The sample stored at -80 °C refrigerator was thawed on ice. A 200 μL methanol/acetonitrile (1:1,v/v) solution containing internal standard were added into the 100 μL sample and vortexed for 5 min. The protein was precipitated at low temperature (-20 °C) for 30 min. The sample was centrifuged at 12000 rpm for 10 min (4 °C). The all supernatant was collected and transferred. Repeat the extraction once and combine the supernatants. The eicosanoids in supernatants were extracted using Poly-Sery MAX SPE columns (ANPEL). Prior to analysis, the eluent was dried under vacuum and redissolved in 100 μL of methanol/water (1:1,v/v) for UPLC/MS/MS analysis.

- 1. *HPLC Conditions*

The sample extracts were analyzed using an LC-ESI-MS/MS system (UPLC, ExionLC AD， <https://sciex.com.cn> /; MS, QTRAP® 6500+ System, <https://sciex.com> /). The analytical conditions were as follows, HPLC: column, Waters ACQUITY UPLC HSS T3 C18 (100 mm×2.1 mm i.d.,1.8 µm); solvent system, water with 0.04% acetic acid (A), acetonitrile with 0.04% acetic acid (B); The gradient was 0-2.0 min from 0.1% to 30% B; 2.0-4.0 min to 50% B; 4.0-5.5 min to 99% B, which was maintained for 1.5 min; and 6.0-7.0 min reduced to 0.1% B and maintained for 3.0 min. flow rate, 0.4 mL/min; temperature, 40 °C; injection volume: 10 μL.

- 1. *ESI-MS/MS Conditions*

Linear ion trap (LIT) and triple quadrupole (QQQ) scans were acquired on a triple quadrupole-linear ion trap mass spectrometer (QTRAP), QTRAP® 6500+ LC-MS/MS System, equipped with an ESI Turbo Ion-Spray interface, operating in negative ion mode and controlled by Analyst 1.6.3 software (Sciex). The ESI source operation parameters were as follows: ion source, ESI-; source temperature 550 ℃; ion spray voltage (IS) -4500 V; curtain gas (CUR) was set at 35 psi, respectively.

- 1. *Article method reference and citation template*

Eicosanoids were analyzed using scheduled multiple reaction monitoring (MRM). Data acquisitions were performed using Analyst 1.6.3 software (Sciex). Multiquant 3.0.3 software (Sciex) was used to quantify all metabolites. Mass spectrometer parameters including the declustering potentials (DP) and collision energies (CE) for individual MRM transitions were done with further DP and CE optimization. A specific set of MRM transitions were monitored for each period according to the metabolites eluted within this period. Detection of eicosanoids Eicosanoids contents were detected by MetWare (<http://www.metware.cn/>) based on the AB Sciex QTRAP 6500 LC-MS/MS platform.

1. ***Detection of cellular ATP levels and protein quantification***

Because the number of cells also affects the ATP levels, and the cell count is rough, we standardized the ATP content through protein quantification, and the final results were represented by the ATP content per milligram of protein (nmol/mg protein). Briefly, Cells are lysed according to the manufacturer’s instructions (S0027, Beyotime Biotechnology), and 20uL cell lysis buffer were extracted for protein quantification. We use the Pierce™ BCA Protein Assay Kit (23225, Thermo Fisher Scientific, Waltham, MA, USA) to measure protein concentration via a standard protocol.

1. ***Immunofluorescence assay (IF)***

To examine the changes of γ-H2A.X in mGCs, immunofluorescence assay was performed using a standard staining procedure. In brief, primary cultured mGCs were cultured on coverslips and fixed with 4% paraformaldehyde. After permeabilization with PBS-T (0.1% Triton X-100 in PBS solution), mGCs were blocked with 5% bovine serum albumin (BSA) for 30 min and then incubated with primary antibodies against phalloidin (1:200, C2205S, Beyotime Biotechnology, China), or γ-H2A.X (1:200, 9718, CST, USA) at 4°C overnight. Fluorescent-conjugated secondary antibody solution (1:100; MULTI SCIENCES, Hangzhou, China) was used to visualize the signal. Cell nuclei were visualized by staining with 4′, 6-diamidino-2-phenylindole (DAPI, H-1200, Vector Laboratories, USA) solution.

1. ***Chromatin immunoprecipitation and real-time qPCR***

We performed chromatin immunoprecipitation (ChIP) assays using the Simple ChIP® Enzymatic Chromatin IP Kit (#9003; CST, Danvers, USA) following the manufacturer’s instructions. Normal rabbit IgG (#2729; CST) and ChIP-grade anti-H3K27Me3 (#9733; CST) were used for IP (1 μg antibody per IP sample). ChIP-enriched DNA were subjected to deep sequencing (ChIP-Seq) before [[3](#_ENREF_3)], and ChIP-PCR was performed to verify the ChIP-Seq results as described before [[11](#_ENREF_11)]. The two ChIP-PCR-related primer sequences are listed in *Supplementary Table 3*. 2% input was used in these experiments as positive control, and after optimize our ChIP-PCR system, Comparative Delta-delta Ct method was used in our research [[12](#_ENREF_12)], so Fold Change in Occupancy was used to show the differential occupancy fold change between WT and KO group.

1. ***Establishment of the endometriosis mouse model***

We established an endometriosis mouse model to clarify the function of 14, 15-EET *in vivo*. Six-week-old female ICR mice (20–24 g) were purchased from Shanghai Animal Centre at the Chinese Academy of Science, and housed in our animal center before initiating experiments. Mice with regular 4- to 5-day estrous cycles were used for further experiments. Allogeneic transplantation surgery for establishment of the endometriosis mouse model was commonly used and optimized in our laboratory [[13-16](#_ENREF_13)]. Briefly, donor ICR mice were pre-treated with 200 µg/kg 17β-estradiol (Sigma-Aldrich, St. Louis, MO, USA) daily by intraperitoneal injection for 7 days as presented in **Supplementary Fig. 6**. The purpose of daily injection of estrogen to the donor mouse is to promote the growth of mouse endometrium [[17](#_ENREF_17)]. The donor uterus was excised from the uterine horn and cervix uteri, two strips were cut longitudinally with micro-scissors, and the endometrial mucosa was exposed [[18](#_ENREF_18)]. Endometrial tissue with an approximate size of 0.5 cm × 0.3 cm was sutured on both ends using 6/0 polypropylene, and then stitched to the bowel serosa of the intestinal mesentery [[14-16](#_ENREF_14)]. In parallel, control ICR mice were sham-operated without implantation of endometrial tissue. To minimize the effect of the environment and surgical techniques, the operation time was limited to 15 min by Xiang Lin and Weijia Gu. Four weeks after transplantation, four mice were randomly selected from Con and EM groups to evaluate the endometriosis incidence of surgery. Implants grew into macroscopic ellipsoidal cysts that contained both endometrial glands and stroma in the EM group [[14](#_ENREF_14), [15](#_ENREF_15)].

Because of the need of later experiments, all receptor mice retained their ovaries and 200 µg/kg 17β-estradiol was administered every other day for 14 days to support the implantation of ectopic cysts [[17](#_ENREF_17), [19](#_ENREF_19)]. To reduce the influence of estrogen on ovulation, a 2-week washout period was administered before induction of ovulation because the effect of estrogen is believed to be maintained for less than 14 days [[17](#_ENREF_17)]. After the washout period, all receptor ICR mice underwent standard ovulation induction at day 30 by PMSG or HCG [[20](#_ENREF_20), [21](#_ENREF_21)].

1. ***Drug treatment and dosage selection***

To further establish causality between 14, 15-EET and PI3K/AKT/mTOR suppression, we reactivate p-AKT by AKT activator SC79 after 14, 15-EET treatment under oxidative stress conditions. The working concentration of SC79 is 20 µM for 24 hours [[22](#_ENREF_22), [23](#_ENREF_23)].

Administration of TPPU with oral led to systemic distribution as well as high drug levels and thus makes chronic EPHX2 enzyme inhibition studies possible [[24](#_ENREF_24)]. Therefore, TPPU is suitable for investigating soluble epoxide hydrolase biology and the role of epoxide-containing lipids *in vivo* [[25-29](#_ENREF_25)]. TPPU displays high plasma concentrations when dosed orally at 0.3 mg/kg and drug-like properties. The maximum concentration increases with dose from 0.3 to 3 mg/kg for TPPU [[30](#_ENREF_30)], and TPPU’s blood concentration increases dose dependently within the treatment period to reach an almost steady state after 8 days [[24](#_ENREF_24)]. Additionally, TPPU (3 mg/kg) is the mostly used method *in vivo* that balance both biological safety and drug efficacy [[25](#_ENREF_25), [26](#_ENREF_26), [31](#_ENREF_31)]. Therefore, we choose 3 mg/kg TPPU for the further research.

BEZ-235 is an orally active, dual pan-class I PI3K and mTOR inhibitor that acts on p110α/γ/δ/β and mTORC1/2. Pharmacokinetic studies have demonstrated maximal tissue concentration one hour after BEZ-235 oral gavage, transient PI3K inhibition and sustained blockade of mTORC1/mTORC2 signaling [[32](#_ENREF_32)]. When the concentration of BZE-235 exceeds the mTOR threshold, it may induce non-specific inhibition of other kinases (such as ART, PDK1) or non-kinase proteins, leading to cross-talk interference in signaling pathways. Furthermore, the off-target effects of BZE-235 could manifest as toxicities in non-targeted tissues, including metabolic disorders (like hyperglycemia and hyperinsulinemia) and hepatorenal impairment (resulting in body weight loss). Oral gavage with 5 or 25 mg/kg of BEZ-235 once a day for 14 days does not alter mouse body weight [[33](#_ENREF_33)]. Although there was no relevant research in endometriosis, our previous pre-experiment found that 20 mg/kg BEZ-235 did not cause side effects such as body weight loss, decrease in ovarian wet weight, or disorder of estrous cycle in mice (**Supplementary Fig. 6G-I**). The treatment dose of 20 mg/kg BEZ-235 is chosen mainly based on the mainstream literatures, which indicates that 20 mg/kg mouse body weight BEZ-235 effectively treats murine tumor models without serious adverse effects [[34-36](#_ENREF_34)]. Moreover, higher doses (30 and 45 mg/kg) causes a weight loss >10% after 10 days of treatment [[37](#_ENREF_37)]. As the PI3K/Akt pathway is involved in insulin-mediated glucose uptake, 45 mg/kg BEZ235 by daily gavage for 2 weeks also slightly cause hyperglycemia and hyperinsulinemia [[38](#_ENREF_38)]. Taking into account the efficacy, safety, metabolic disorders and other side effects comprehensively, the dose of 20 mg/kg is used for further studies.

As described in **Supplementary Figure 6A**, the day of endometrium transplantation surgery was defined as day 0. TPPU was dissolved in 1% PEG 400 and BEZ-235 was dissolved in 40% PEG 400. The receptor mice in the TEM group were given 3 mg/kg TPPU by oral gavage twice a day. The receptor mice in the BEM group were given 20 mg/kg BEZ-235 by oral gavage daily. Oral gavage TPPU or BEZ-235 was started at day 14 and ended at mGC harvest or mating (at day 28-30).

1. ***Estrous cycle patters detection***

The estrous cycle pattern of three mouse from EM group, TEM group, BEM group were detected via direct smear method at 6 p.m daily. In brief, cotton swab was moistened with PBS and insert into the vagina of mouse by 0.5-1.0cm, and rotate along the vaginal wall for 4 circles. Then spread the cotton swab evenly on the slide to form a thin layer. Let the slides air dry naturally and then fix them with 95% ethanol for 5 minutes. Immediately observe cell morphology to define the physiological cycle of mouse under a microscope. We randomly selected three mice from each group for this experiment, and the typical estrous cycle pattern of one mouse was presented in **Supplementary Figure 6E** and **Supplementary Figure 6I**.

1. ***COC isolation and in vitro COC expansion assay***

Briefly, mice were primed with PMSG for 44-46h. COCs were released by needle puncture and collected separately from well-developed graafian follicles via 26.5-gauge needles. For in vitro COC expansion experiments, non-expanded COCs were plated in 40 µL defined COC medium, MEM (11095080, Thermo Fisher) supplemented with 25 mM HEPES (MA0036, Meilunbio), 0.25 mM sodium pyruvate (11360070, Thermo Fisher), 3 mM L-glutamine (25030081, Thermo Fisher), 1 mg/ml BSA (SRE0098, Sigma-Aldrich, St. Louis, MO, USA), 1× penicillin and streptomycin (P1400, Solarbio), and 1% FBS under the cover of mineral oil (Sigma). COCs were cultured under humidified conditions at 37 °C and 5% CO2. After 12 h, COCs expansion was visualized using a light microscope. The COCs expansion status was observed by microscopy and evaluated by measuring the diameter of the COCs using ImageJ software (Image JFiji, [https://imagej.net/Fiji) as](https://imagej.net/Fiji)%20as) previously described [[3](#_ENREF_3), [10](#_ENREF_10), [39](#_ENREF_39), [40](#_ENREF_40)].

1. ***RNA Sequencing and Bioinformatics Analysis***

High-throughput sequencing and bioinformatics analyses were conducted at Cosmos Wisdom (Hangzhou, China). The RNA-sequencing process mainly includes sample detection, library construction, quality control, and sequencing on a high-throughput sequencing platform. The sequencing platform is Illumina Novaseq 6000, the read length was carried out at 150bp for both ends, and deseq2 was applied to process the data and achieve multiple corrections. The number of biological replicates has been described in the materials and methods section.


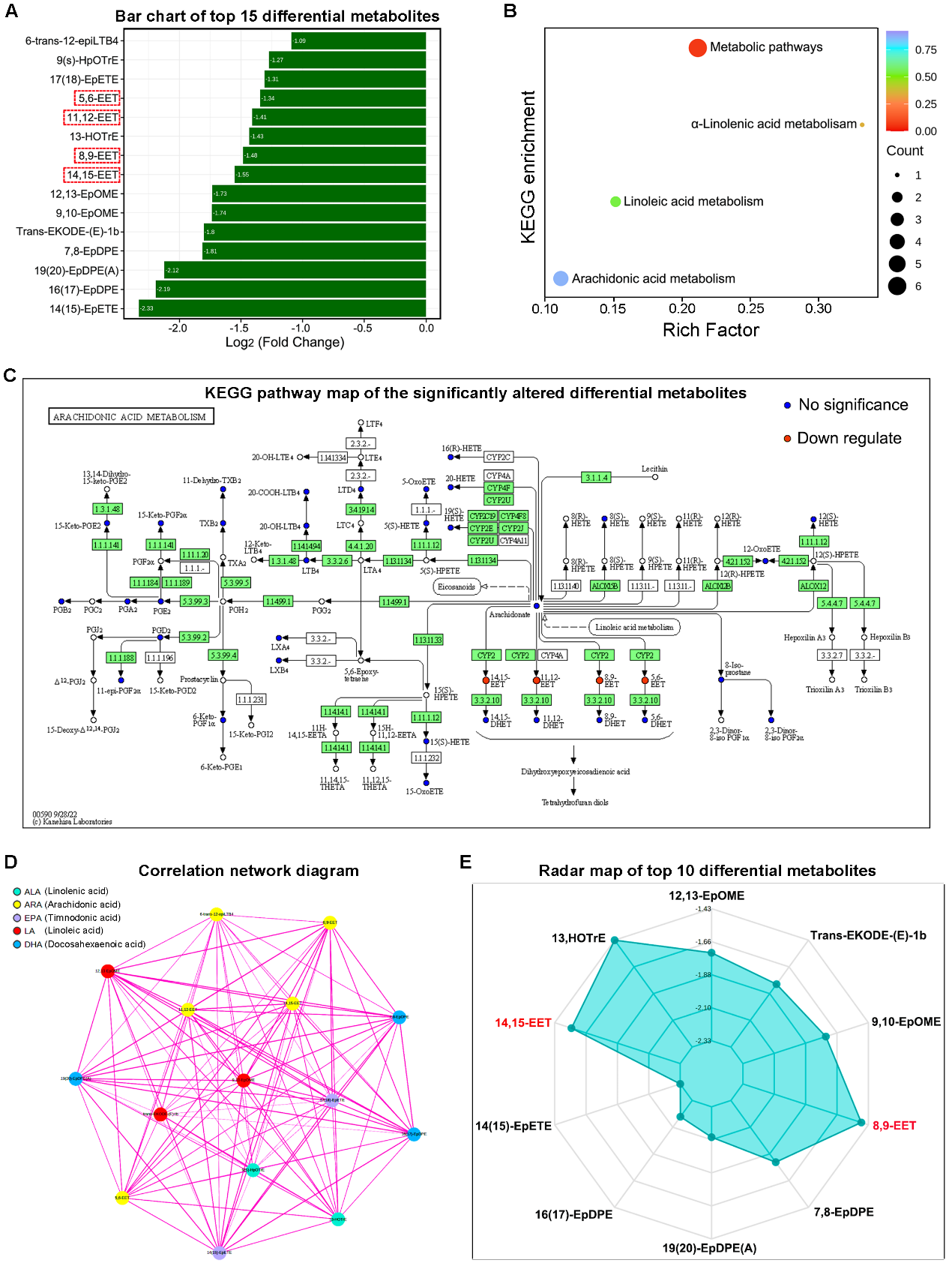


**Supplementary Fig. 1.**

From the liquid chromatography tandem mass spectrometry (LC-MS/MS) analysis results for 101 follicular fluid (FF) samples, we performed KEGG functional annotation and enrichment analysis on the differential metabolites in our dataset. (**A**) Bar chart showing the top 15 differential metabolites. The X-axis represents the log2 fold change (log_2_ Fold change) of the differential metabolites and the Y-axis represents the differential metabolites. Yellow color represents significantly upregulated metabolites (none were observed in our results), while green color represents significantly downregulated metabolites (four EETs were included). (**B**) Differential metabolite KEGG enrichment bubble plot. The X-axis represents the Rich Factor for each pathway. The size of the dots represents the number of differentially enriched metabolites. (**C**) The most significant metabolite KEGG pathway map was “Arachidonic acid metabolism.” Red indicates significantly downregulated metabolites in the EM-FF group (four EETs were found), blue represents metabolites detected but not significantly changed, and yellow indicates significantly upregulated metabolites in the EM-FF group. (**D**) Correlation network diagram of the 15 top differential metabolites. (**E**) Radar chart of the top 10 differential metabolites.


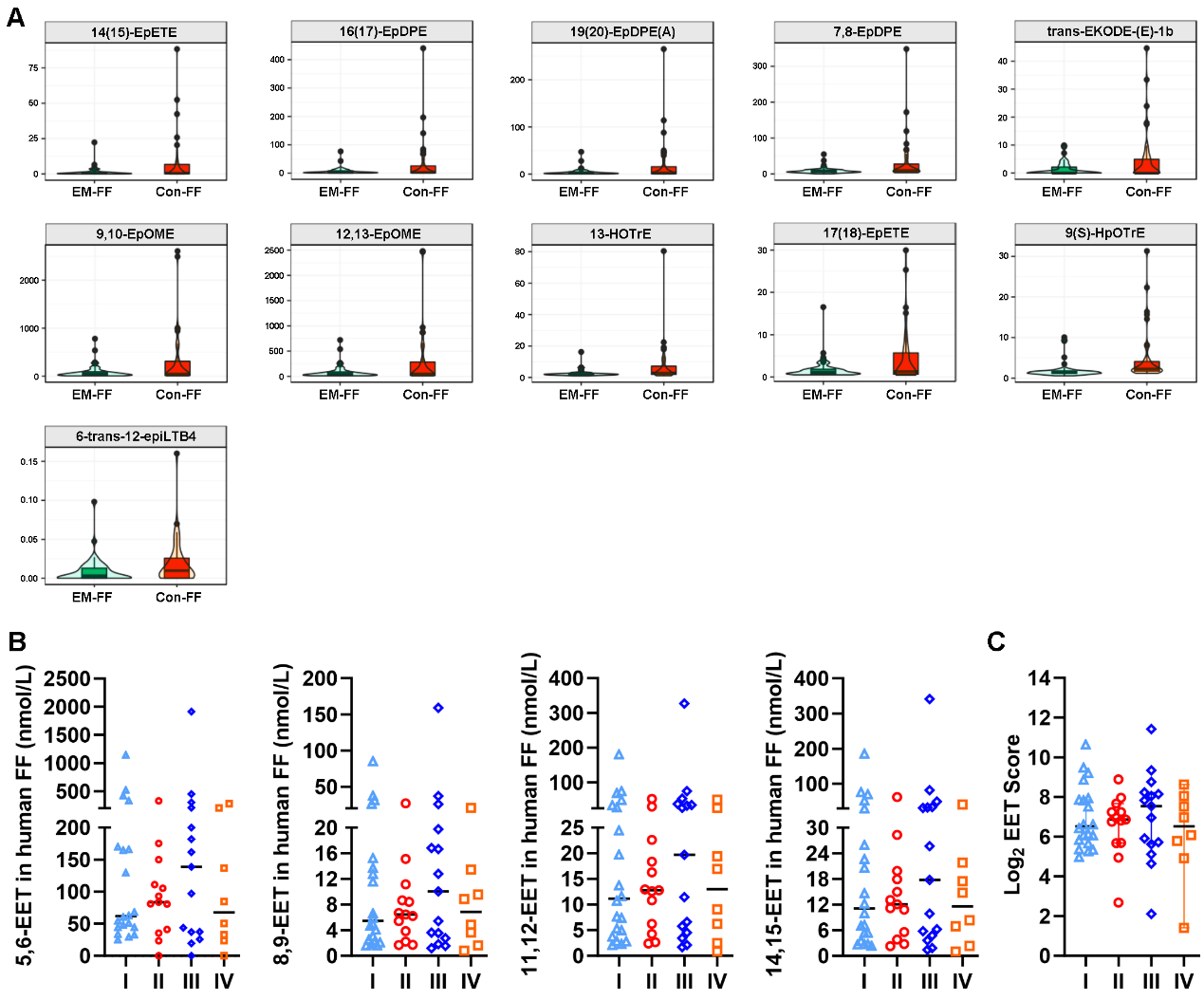


**Supplementary Fig. 2.**

**(A)** Violin plots of another 11 differential metabolites in EM-follicular fluid (FF) samples (fold change > 2, corrected *P*-value < 0.05; n = 45 for Con group, n = 56 for EM group). (**B**) Data from 56 FF samples from EM patients suggested no difference in EET levels between EM stages (stages I–IV), n = 20 for stage I, n = 13 for stage II, n = 15 for stage III, n = 8 for stage IV. Unpaired *t*-test. (**C**) No difference in EET score was observed between EM stages. Unpaired *t*-test.


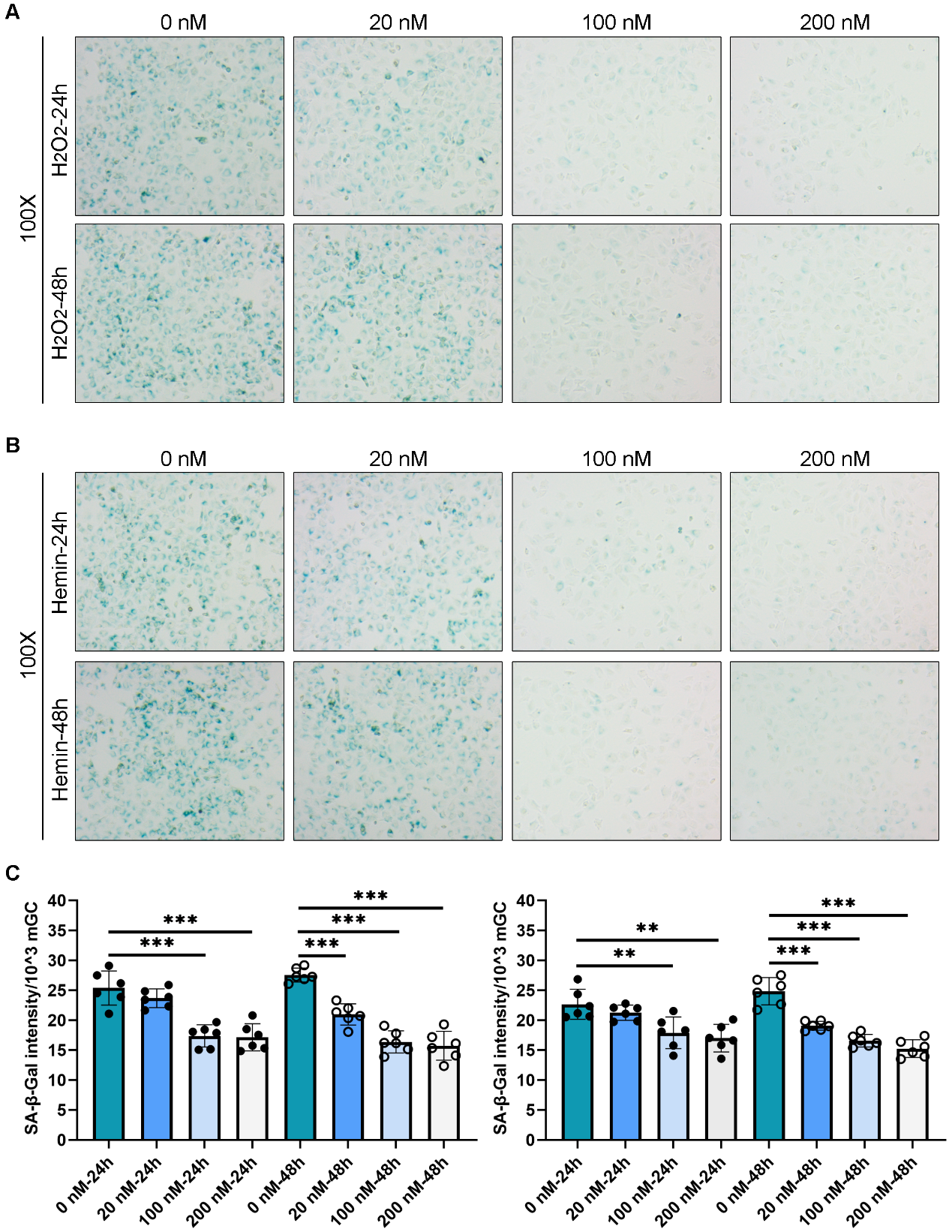


**Supplementary Fig. 3.**

(**A**) Representative SA-β-gal staining assay image for mouse granulosa cells (mGCs) after 0, 20, 100, or 200 nM 14, 15-EET pre-treatment for 24 or 48 hours followed by 100 µM H_2_O_2_ treatment for another 24 hours. Magnification, 100×. (**B**) Representative SA-β-gal staining assay image for mGCs after 0, 20, 100, or 200 nM 14, 15-EET pre-treatment for 24 or 48 hours followed by 10 µM hemin treatment for another 24 hours. Magnification, 100×. (**C**) SA-β-gal quantitative assay of mGCs after different concentrations of 14, 15-EET pre-treatment followed by H_2_O_2_ or hemin treatment for another 24 hours. Paired *t*-test, ***P* < 0.01, ****P* < 0.001.


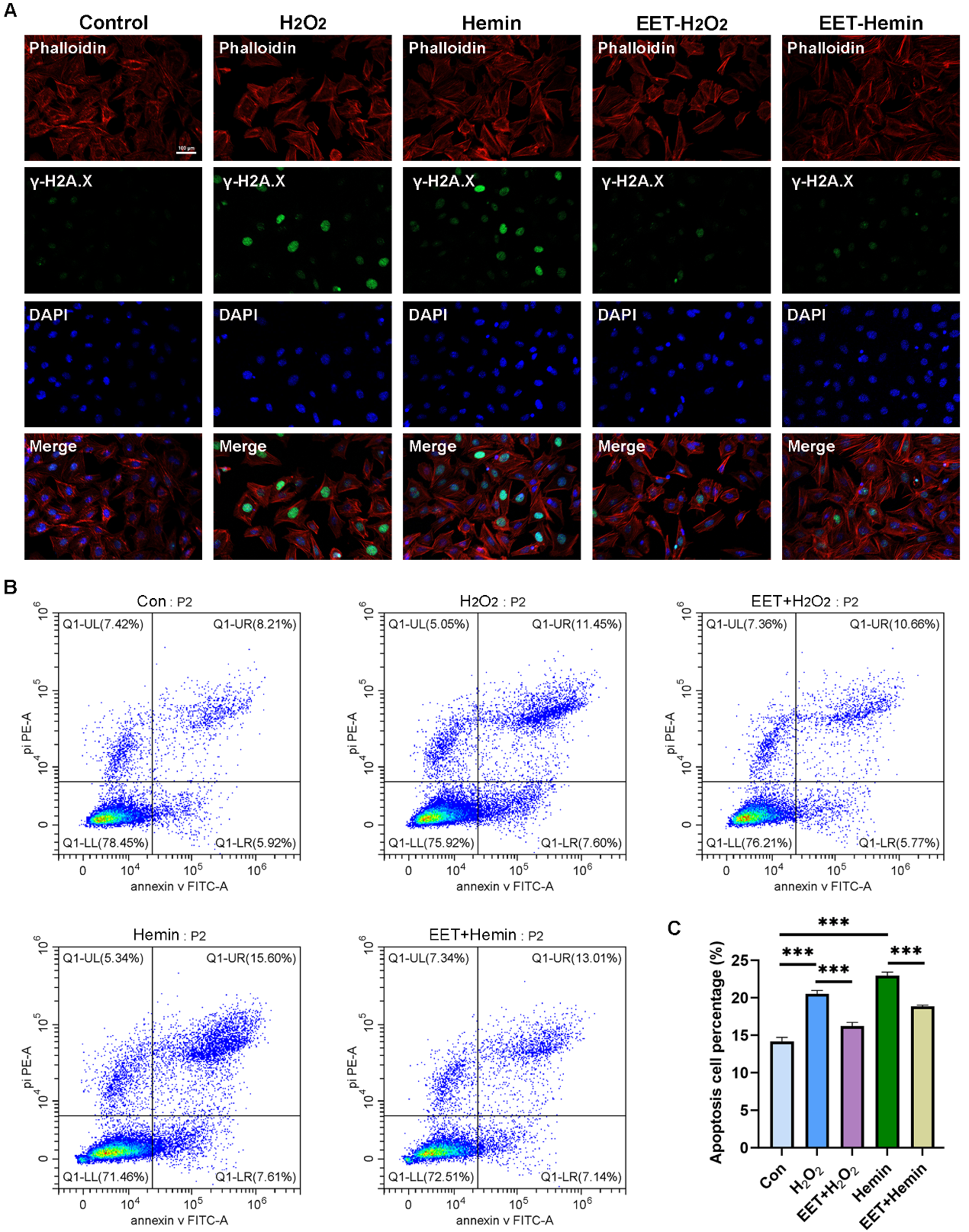


**Supplementary Fig. 4.**

(**A**) Immunofluorescence staining for γ-H2A.X protein in mouse granulosa cells (mGCs) after 14, 15-EET pre-treatment for 24 hours followed by H_2_O_2_ or hemin treatment for 24 hours. Representative photographs were taken using confocal microscopy. Scale bar 100 µm, Original magnification, 100×. (**B–C**) Flow cytometry analysis showing the percentage of apoptotic mGCs after 14, 15-EET pre-treatment for 24 hours followed by H_2_O_2_ or hemin treatment for 48 hours. Paired *t*-test, ****P* < 0.001.


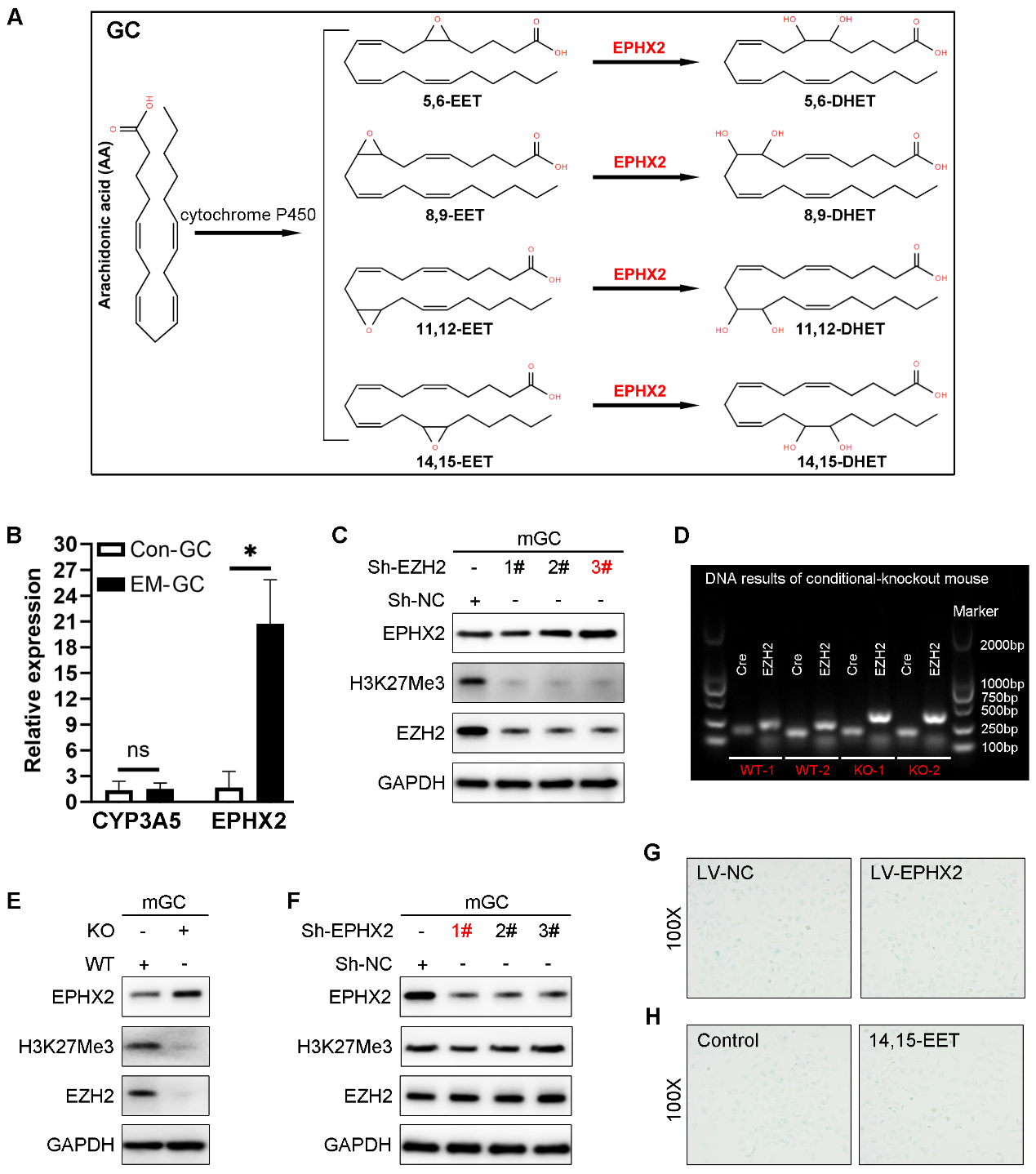


**Supplementary Fig. 5**.

(**A**) For arachidonic acid (AA) metabolism, P450 oxidases convert AA into EETs, which are then metabolized into DHETs by EPHX2. (**B**) qRT-PCR results of CYP3A5 and EPHX2 mRNA expression levels in human primary granulosa cells (GCs) (18 Con-GCs vs. 30 EM-GCs, Mann-Whitney test, **P* < 0.05). (**C**) Western blot analysis was used to detect the knockout efficiency of three different Sh-EZH2. Sh-EZH2-3# was selected for the subsequent experiments. (**D**) Genotype identification of GC-specific knockout mice via DNA electrophoresis. Mice with GC-specific *Ezh2* knockout (KO mice) were generated by crossing *Cyp19a1-Cre* mice with *Ezh2^flox/flox^* mice. (**E**) Western blot analysis of EZH2, H3K27Me3, and EPHX2 protein levels in ovary GCs from wild-type (WT) and KO mice. (**F**) Western blot analysis was used to detect the knockout efficiency of three different Sh-EPHX2. Sh-EPHX2-1# was selected for the subsequent experiments. (**G**) SA-β-gal staining assay of mGCs after LV-NC or LV-EPHX2 treatment for 24 hours in normal culture. Magnification, 100×. (**H**) SA-β-gal staining assay of mGCs under normal culture or after 14, 15-EET co-culture for 24 hours. Magnification, 100×.


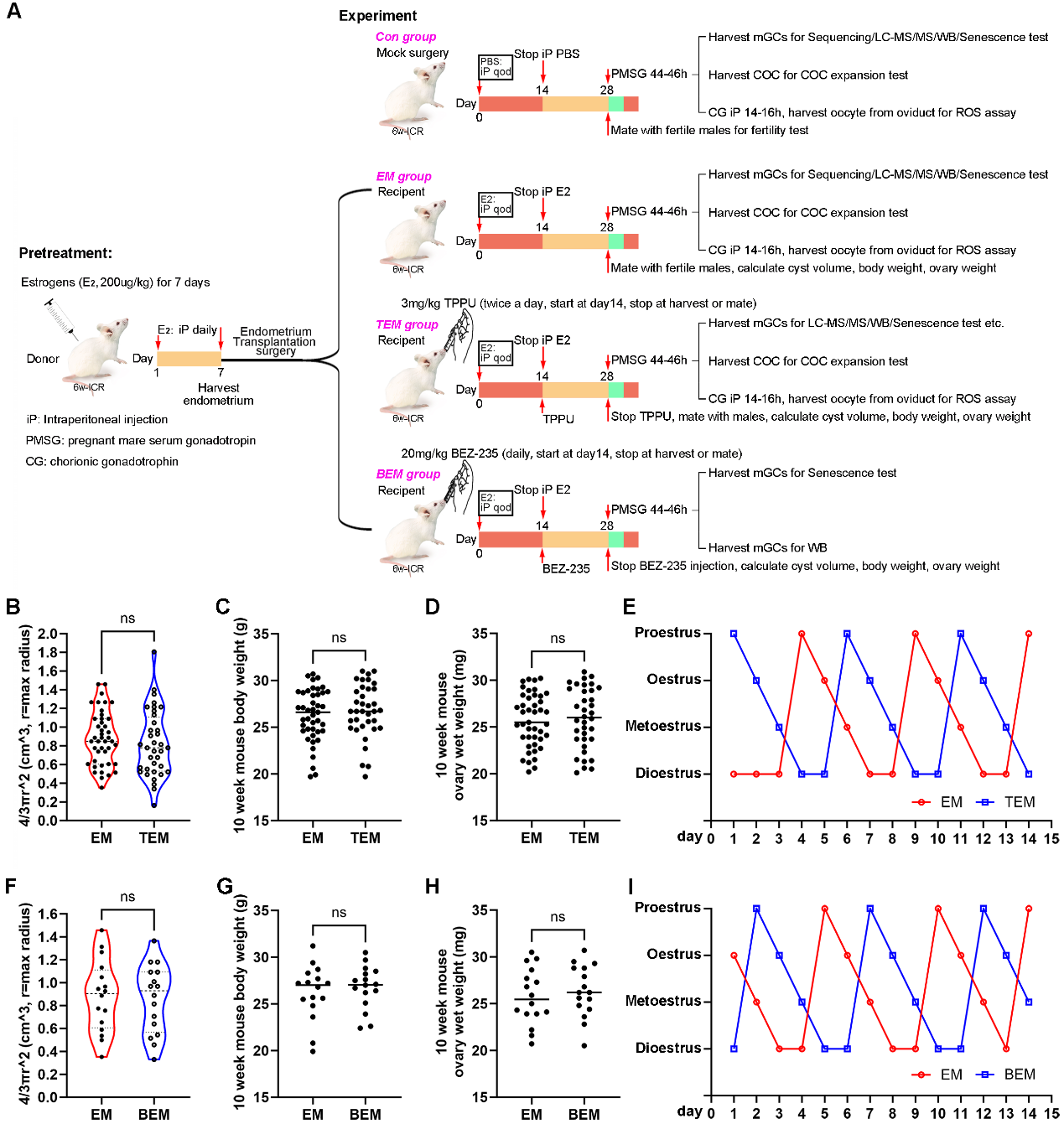


**Supplementary Fig. 6.**

(**A**) Detailed flow charts for the endometriosis (EM) mouse model establishment, TPPU or BEZ-235 administration, and other animal experiments. (**B**) The scatter plot of lesion volumes from EM and TEM mice (n = 43 for EM group, n = 36 for TEM group; the seven EM mice and seven TEM mice used for the fertility tests were not included). Unpaired *t*-test, no statistical significance. (**C**) Body weights of 10-week-old EM and TEM mice (n = 43 for EM group, n = 36 for TEM group). Unpaired *t*-test, no statistical significance. (**D**) Ovary wet weights of 10-week-old EM and TEM mice (n = 43 for EM group, n = 36 for TEM group, each dot represents the average wet weight of two ovaries from one mouse). Unpaired *t*-test, no statistical significance. (**E**) Estrous cycle patterns of EM and TEM mice within 14 days. The horizontal axis shows the time (day) of observation, while the vertical axis indicates the four menstrual cycle stages. (**F**) The scatter plot of lesion volumes from EM and BEM mice (n = 16 for EM group, n = 16 for BEM group). Unpaired *t*-test, no statistical significance. (**G**) Body weights of 10-week-old EM and BEM mice (n = 16 for EM group, n = 16 for BEM group). Unpaired *t*-test, no statistical significance. (**H**) Ovary wet weights of 10-week-old EM and BEM mice (n = 16 for EM group, n = 16 for BEM group). Unpaired *t*-test, no statistical significance. (**I**) Estrous cycle patterns of EM and BEM mice within 14 days. The horizontal axis shows the time (day) of observation, while the vertical axis indicates the four menstrual cycle stages.


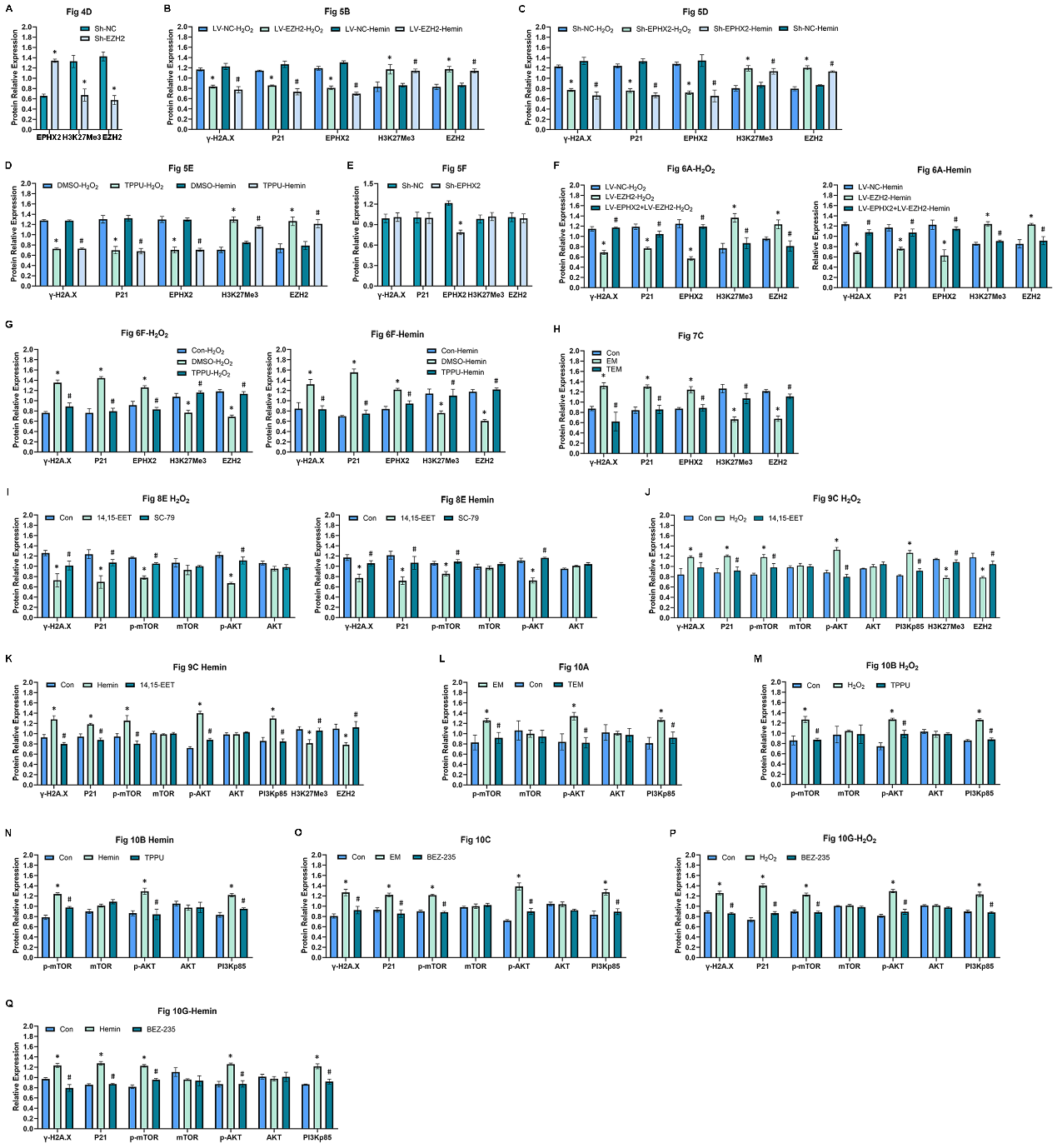


**Supplementary Fig. 7.**

(**A**) Statistical analysis of the western blot results in **Figure 4D**, **P* < 0.05, Paired *t*-test, Sh-EZH2 *vs.* Sh-NC. (**B**) Statistical analysis of the western blot results in **Figure 5B**, **P* < 0.05, Paired *t*-test, LV-EZH2 *vs.* LV-NC after H_2_O_2_ treatment; #*P* < 0.05, Paired *t*-test, LV-EZH2 *vs.* LV-NC after Hemin treatment. (**C**) Statistical analysis of the western blot results in **Figure 5D**, **P* < 0.05, Paired *t*-test, Sh-EPHX2 *vs.* Sh-NC after H_2_O_2_ treatment; #*P* < 0.05, Paired *t*-test, Sh-EPHX2 *vs.* Sh-NC after Hemin treatment. (**D**) Statistical analysis of the western blot results in **Figure 5E**, **P* < 0.05, Paired *t*-test, TPPU *vs.* DMSO after H_2_O_2_ treatment; #*P* < 0.05, Paired *t*-test, TPPU *vs.* DMSO after Hemin treatment. (**E**) Statistical analysis of the western blot results in **Figure 5F**, **P* < 0.05, Paired *t*-test, Sh-EPHX2 *vs.* Sh-NC. (**F**) Statistical analysis of the western blot results in **Figure 6A**, **P* < 0.05, Paired *t*-test, LV-EZH2 *vs.* LV-NC after H_2_O_2_ or Hemin treatment; #*P* < 0.05, Paired *t*-test, LV-EPHX2+LV-EZH2 *vs.* LV-EZH2 after H_2_O_2_ or Hemin treatment. (**G**) Statistical analysis of the western blot results in **Figure 6F**, **P* < 0.05, Paired *t*-test, DMSO *vs.* Control after H_2_O_2_ or Hemin treatment or no treatment; #*P* < 0.05, Paired *t*-test, TPPU *vs.* DMSO after H_2_O_2_ or Hemin treatment. (**H**) Statistical analysis of the western blot results in **Figure 7C**, **P* < 0.05, Paired *t*-test, EM *vs.* Control; #*P* < 0.05, Paired *t*-test, TEM *vs.* EM mice. (**I**) Statistical analysis of the western blot results in **Figure 8E**, **P* < 0.05, Paired *t*-test, 14, 15-EET *vs.* Control after H_2_O_2_ or Hemin treatment; #*P* < 0.05, Paired *t*-test, SC-79 + 14, 15-EET *vs.* 14, 15-EET after H_2_O_2_ or Hemin treatment. (**J**) Statistical analysis of the western blot results in **Figure 9C (left)**, **P* < 0.05, Paired *t*-test, H_2_O_2_ *vs.* Control; #*P* < 0.05, Paired *t*-test, 14, 15-EET + H_2_O_2_ *vs.* H_2_O_2_. (**K**) Statistical analysis of the western blot results in **Figure 9C (right)**, **P* < 0.05, Paired *t*-test, Hemin *vs.* Control; #*P* < 0.05, Paired *t*-test, 14, 15-EET + Hemin *vs.* Hemin. (**L**) Statistical analysis of the western blot results in **Figure 10A**, **P* < 0.05, Paired *t*-test, EM *vs.* Control mice; #*P* < 0.05, Paired *t*-test, TEM *vs.* EM mice. (**M**) Statistical analysis of the western blot results in **Figure 10B (left)**, **P* < 0.05, Paired *t*-test, H_2_O_2_ *vs.* Control; #*P* < 0.05, Paired *t*-test, TPPU + H_2_O_2_ *vs.* H_2_O_2_. (**N**) Statistical analysis of the western blot results in **Figure 10B (right)**, **P* < 0.05, Paired *t*-test, Hemin *vs.* Control; #*P* < 0.05, Paired *t*-test, TPPU + Hemin *vs.* Hemin. (**O**) Statistical analysis of the western blot results in **Figure 10C**, **P* < 0.05, Paired *t*-test, EM *vs.* Control mice; #*P* < 0.05, Paired *t*-test, BEZ-235 *vs.* EM mice. (**P**) Statistical analysis of the western blot results in **Figure 10G (left)**, **P* < 0.05, Paired *t*-test, H_2_O_2_ *vs.* Control; #*P* < 0.05, Paired *t*-test, BEZ-235 + H_2_O_2_ *vs.* H_2_O_2_. (**Q**) Statistical analysis of the western blot results in **Figure 10G (right)**, **P* < 0.05, Paired *t*-test, Hemin *vs.* Control; #*P* < 0.05, Paired *t*-test, BEZ-235 + Hemin *vs.* Hemin.

***Reference:***

1. Zegers-Hochschild F, Adamson GD, Dyer S, Racowsky C, de Mouzon J, Sokol R, Rienzi L, Sunde A, Schmidt L, Cooke ID *et al*: **The International Glossary on Infertility and Fertility Care, 2017**. *Human reproduction* 2017, **32**(9):1786-1801.

2. International working group of Aagl EE, Wes, Tomassetti C, Johnson NP, Petrozza J, Abrao MS, Einarsson JI, Horne AW, Lee TTM, Missmer S *et al*: **An International Terminology for Endometriosis, 2021**. *Journal of minimally invasive gynecology* 2021, **28**(11):1849-1859.

3. Lin X, Tong XM, Zhang YL, Gu WJ, Huang QM, Zhang Y, Zhuo F, Zhao FX, Jin XY, Li C *et al*: **Decreased Expression of EZH2 in Granulosa Cells Contributes to Endometriosis-Associated Infertility by Targeting IL-1R2**. *Endocrinology* 2022, **164**(2).

4. Bourdon M, Santulli P, Maignien C, Gayet V, Pocate-Cheriet K, Marcellin L, Chapron C: **The deferred embryo transfer strategy improves cumulative pregnancy rates in endometriosis-related infertility: A retrospective matched cohort study**. *PloS one* 2018, **13**(4):e0194800.

5. Kong H, Hu L, Nie L, Yu X, Dai W, Li J, Chen C, Bu Z, Shi H, Wu Q *et al*: **A multi-center, randomized controlled clinical trial of the application of a shortened protocol of long-acting Triptorelin down-regulated prior to IVF/ICSI among patients with endometriosis: A protocol**. *Reproductive health* 2018, **15**(1):213.

6. Jiang S, Kuang Y: **The effects of low-dose human chorionic gonadotropin combined with human menopausal gonadotropin protocol on women with hypogonadotropic hypogonadism undergoing ovarian stimulation for in vitro fertilization**. *Clinical endocrinology* 2018, **88**(1):77-87.

7. Cantineau AE, Cohlen BJ, Heineman MJ: **Ovarian stimulation protocols (anti-oestrogens, gonadotrophins with and without GnRH agonists/antagonists) for intrauterine insemination (IUI) in women with subfertility**. *The Cochrane database of systematic reviews* 2007(2):CD005356.

8. Peeraer K, Debrock S, De Loecker P, Tomassetti C, Laenen A, Welkenhuysen M, Meeuwis L, Pelckmans S, Mol BW, Spiessens C *et al*: **Low-dose human menopausal gonadotrophin versus clomiphene citrate in subfertile couples treated with intrauterine insemination: a randomized controlled trial**. *Human reproduction* 2015, **30**(5):1079-1088.

9. Scott L, Alvero R, Leondires M, Miller B: **The morphology of human pronuclear embryos is positively related to blastocyst development and implantation**. *Human reproduction* 2000, **15**(11):2394-2403.

10. Zhang YL, Xia Y, Yu C, Richards JS, Liu J, Fan HY: **CBP-CITED4 is required for luteinizing hormone-triggered target gene expression during ovulation**. *Mol Hum Reprod* 2014, **20**(9):850-860.

11. Lin X, Dai Y, Gu W, Zhang Y, Zhuo F, Zhao F, Jin X, Li C, Huang D, Tong X *et al*: **The involvement of RNA N6-methyladenosine and histone methylation modification in decidualization and endometriosis-associated infertility**. *Clin Transl Med* 2024, **14**(2):e1564.

12. Rao X, Huang X, Zhou Z, Lin X: **An improvement of the 2^(-delta delta CT) method for quantitative real-time polymerase chain reaction data analysis**. *Biostat Bioinforma Biomath* 2013, **3**(3):71-85.

13. Pelch KE, Sharpe-Timms KL, Nagel SC: **Mouse model of surgically-induced endometriosis by auto-transplantation of uterine tissue**. *Journal of visualized experiments : JoVE* 2012(59):e3396.

14. Dai Y, Lin X, Xu W, Lin X, Huang Q, Shi L, Pan Y, Zhang Y, Zhu Y, Li C *et al*: **MiR-210-3p protects endometriotic cells from oxidative stress-induced cell cycle arrest by targeting BARD1**. *Cell Death Dis* 2019, **10**(2):144.

15. Lin X, Dai Y, Tong X, Xu W, Huang Q, Jin X, Li C, Zhou F, Zhou H, Lin X *et al*: **Excessive oxidative stress in cumulus granulosa cells induced cell senescence contributes to endometriosis-associated infertility**. *Redox Biol* 2020, **30**:101431.

16. Zhou F, Zhao F, Huang Q, Lin X, Zhang S, Dai Y: **NLRP3 activated macrophages promote endometrial stromal cells migration in endometriosis**. *Journal of reproductive immunology* 2022, **152**:103649.

17. Galvankar M, Singh N, Modi D: **Estrogen is essential but not sufficient to induce endometriosis**. *Journal of biosciences* 2017, **42**(2):251-263.

18. Uegaki T, Taniguchi F, Nakamura K, Osaki M, Okada F, Yamamoto O, Harada T: **Inhibitor of apoptosis proteins (IAPs) may be effective therapeutic targets for treating endometriosis**. *Human reproduction* 2015, **30**(1):149-158.

19. Li J, Dai Y, Zhu H, Jiang Y, Zhang S: **Endometriotic mesenchymal stem cells significantly promote fibrogenesis in ovarian endometrioma through the Wnt/beta-catenin pathway by paracrine production of TGF-beta1 and Wnt1**. *Human reproduction* 2016, **31**(6):1224-1235.

20. Fan HY, O'Connor A, Shitanaka M, Shimada M, Liu Z, Richards JS: **Beta-catenin (CTNNB1) promotes preovulatory follicular development but represses LH-mediated ovulation and luteinization**. *Molecular endocrinology* 2010, **24**(8):1529-1542.

21. Fan HY, Liu Z, Johnson PF, Richards JS: **CCAAT/enhancer-binding proteins (C/EBP)-alpha and -beta are essential for ovulation, luteinization, and the expression of key target genes**. *Molecular endocrinology* 2011, **25**(2):253-268.

22. So EY, Ouchi T: **BRAT1 deficiency causes increased glucose metabolism and mitochondrial malfunction**. *BMC Cancer* 2014, **14**:548.

23. Jo H, Mondal S, Tan D, Nagata E, Takizawa S, Sharma AK, Hou Q, Shanmugasundaram K, Prasad A, Tung JK *et al*: **Small molecule-induced cytosolic activation of protein kinase Akt rescues ischemia-elicited neuronal death**. *Proc Natl Acad Sci U S A* 2012, **109**(26):10581-10586.

24. Ostermann AI, Herbers J, Willenberg I, Chen R, Hwang SH, Greite R, Morisseau C, Gueler F, Hammock BD, Schebb NH: **Oral treatment of rodents with soluble epoxide hydrolase inhibitor 1-(1-propanoylpiperidin-4-yl)-3-[4-(trifluoromethoxy)phenyl]urea (TPPU): Resulting drug levels and modulation of oxylipin pattern**. *Prostaglandins Other Lipid Mediat* 2015, **121**(Pt A):131-137.

25. Gao L, Chen W, Li L, Li J, Kongling W, Zhang Y, Yang X, Zhao Y, Bai J, Wang F: **Targeting soluble epoxide hydrolase promotes osteogenic-angiogenic coupling via activating SLIT3/HIF-1alpha signalling pathway**. *Cell Prolif* 2023, **56**(7):e13403.

26. Kong L, Li J, Bai Y, Xu S, Zhang L, Chen W, Gao L, Wang F: **Inhibition of soluble epoxide hydrolase enhances the dentin-pulp complex regeneration mediated by crosstalk between vascular endothelial cells and dental pulp stem cells**. *J Transl Med* 2024, **22**(1):61.

27. Codony S, Pont C, Grinan-Ferre C, Di Pede-Mattatelli A, Calvo-Tusell C, Feixas F, Osuna S, Jarne-Ferrer J, Naldi M, Bartolini M *et al*: **Discovery and In Vivo Proof of Concept of a Highly Potent Dual Inhibitor of Soluble Epoxide Hydrolase and Acetylcholinesterase for the Treatment of Alzheimer's Disease**. *J Med Chem* 2022, **65**(6):4909-4925.

28. Napimoga MH, Rocha EP, Trindade-da-Silva CA, Demasi APD, Martinez EF, Macedo CG, Abdalla HB, Bettaieb A, Haj FG, Clemente-Napimoga JT *et al*: **Soluble epoxide hydrolase inhibitor promotes immunomodulation to inhibit bone resorption**. *J Periodontal Res* 2018, **53**(5):743-749.

29. **Correction for Pu et al., Maternal glyphosate exposure causes autism-like behaviors in offspring through increased expression of soluble epoxide hydrolase**. *Proc Natl Acad Sci U S A* 2021, **118**(5).

30. Ulu A, Appt S, Morisseau C, Hwang SH, Jones PD, Rose TE, Dong H, Lango J, Yang J, Tsai HJ *et al*: **Pharmacokinetics and in vivo potency of soluble epoxide hydrolase inhibitors in cynomolgus monkeys**. *Br J Pharmacol* 2012, **165**(5):1401-1412.

31. Wan D, Yang J, McReynolds CB, Barnych B, Wagner KM, Morisseau C, Hwang SH, Sun J, Blocher R, Hammock BD: **In vitro and in vivo Metabolism of a Potent Inhibitor of Soluble Epoxide Hydrolase, 1-(1-Propionylpiperidin-4-yl)-3-(4-(trifluoromethoxy)phenyl)urea**. *Front Pharmacol* 2019, **10**:464.

32. Roper J, Richardson MP, Wang WV, Richard LG, Chen W, Coffee EM, Sinnamon MJ, Lee L, Chen PC, Bronson RT *et al*: **The dual PI3K/mTOR inhibitor NVP-BEZ235 induces tumor regression in a genetically engineered mouse model of PIK3CA wild-type colorectal cancer**. *PLoS One* 2011, **6**(9):e25132.

33. Tian L, Qiao Y, Lee P, Wang L, Chang A, Ravi S, Rogers TA, Lu L, Singhana B, Zhao J *et al*: **Antitumor efficacy of liposome-encapsulated NVP-BEZ 235 in combination with irreversible electroporation**. *Drug Deliv* 2018, **25**(1):668-678.

34. McMillin DW, Ooi M, Delmore J, Negri J, Hayden P, Mitsiades N, Jakubikova J, Maira SM, Garcia-Echeverria C, Schlossman R *et al*: **Antimyeloma activity of the orally bioavailable dual phosphatidylinositol 3-kinase/mammalian target of rapamycin inhibitor NVP-BEZ235**. *Cancer Res* 2009, **69**(14):5835-5842.

35. Derwich A, Sykutera M, Brominska B, Rubis B, Ruchala M, Sawicka-Gutaj N: **The Role of Activation of PI3K/AKT/mTOR and RAF/MEK/ERK Pathways in Aggressive Pituitary Adenomas-New Potential Therapeutic Approach-A Systematic Review**. *Int J Mol Sci* 2023, **24**(13).

36. Wu YY, Wu HC, Wu JE, Huang KY, Yang SC, Chen SX, Tsao CJ, Hsu KF, Chen YL, Hong TM: **The dual PI3K/mTOR inhibitor BEZ235 restricts the growth of lung cancer tumors regardless of EGFR status, as a potent accompanist in combined therapeutic regimens**. *J Exp Clin Cancer Res* 2019, **38**(1):282.

37. Lee M, Wiedemann T, Gross C, Leinhauser I, Roncaroli F, Braren R, Pellegata NS: **Targeting PI3K/mTOR Signaling Displays Potent Antitumor Efficacy against Nonfunctioning Pituitary Adenomas**. *Clin Cancer Res* 2015, **21**(14):3204-3215.

38. Gallagher EJ, Fierz Y, Vijayakumar A, Haddad N, Yakar S, LeRoith D: **Inhibiting PI3K reduces mammary tumor growth and induces hyperglycemia in a mouse model of insulin resistance and hyperinsulinemia**. *Oncogene* 2012, **31**(27):3213-3222.

39. Liu Z, Fan HY, Wang Y, Richards JS: **Targeted disruption of Mapk14 (p38MAPKalpha) in granulosa cells and cumulus cells causes cell-specific changes in gene expression profiles that rescue COC expansion and maintain fertility**. *Mol Endocrinol* 2010, **24**(9):1794-1804.

40. Dai Y, Lin X, Liu N, Shi L, Zhuo F, Huang Q, Gu W, Zhao F, Zhang Y, Zhang Y *et al*: **Integrative analysis of transcriptomic and metabolomic profiles reveals abnormal phosphatidylinositol metabolism in follicles from endometriosis-associated infertility patients**. *J Pathol* 2023, **260**(3):248-260.

**Table 1.** Clinical characteristics and outcomes of all included infertility patients.

| **Characteristics** | **Control** | **Endometriosis** | ***P* value** |
| --- | --- | --- | --- |
| **Patients number** | 101 | 137 |  |
| **Age (years)** | 33.11±4.73 | 32.68±4.67 | 0.646^a^ |
| **BMI (kg/m^2^)** | 22.49±3.24 | 20.99±2.71 | ***0.013^a^*** |
| **Infertility time (years)** | 2.88±2.86 | 2.78±2.03 | 0.573^b^ |
| **Menstrual cycle (days)** | 30.18±3.70 | 29.32±3.77 | 0.255^a^ |
| **Menstrual period (days)** | 5.98±1.20 | 5.84±1.79 | 0.733^b^ |
| **Ovarian reserve** |  |  |  |
| AMH after surgery（ng/ml） | 3.54±1.95 | 3.03±1.97 | 0.200^a^ |
| Basic Antral Follicle Count | 8.31±4.30 | 7.88±4.86 | 0.638^a^ |
| **High-density lipoprotein (mmol/L)** | 1.47±0.40 | 1.46±0.27 | 0.883^a^ |
| **Low-density lipoprotein (mmol/L)** | 2.95±0.68 | 2.80±0.54 | 0.417^a^ |
| **Total cholesterol (mmol/L)** | 4.86±1.37 | 4.71±0.66 | 0.987^b^ |
| **Triglycerides (mmol/L)** | 1.74±1.13 | 0.97±0.41 | ***0.002^b^*** |
| **EM stage** |  |  |  |
| I | / | 42 |  |
| II | / | 34 |  |
| III | / | 39 |  |
| IV | / | 22 |  |
| **rAFS scores** | / | 21.75±20.64 |  |
| **Serum CA-125 Antigen (U/mL)** | 15.55±7.35 | 22.10±15.11 | ***0.005^b^*** |
| **ART methods (Number)** |  |  |  |
| IVF | 68 | 91 |  |
| ICSI | 33 | 46 |  |
| **Fasting glucose** | 5.05±0.08 | 5.16±0.08 | 0.323^a^ |
| **Oocyte retrieval (Number)** | 9.16±0.75 | 6.68±0.63 | ***0.012^a^*** |
| **Mature oocytes (Number)** | 8.56±0.72 | 6.04±0.62 | ***0.009^a^*** |
| **2 PN embryos (Number)** | 6.51±0.75 | 4.56±0.51 | ***0.028^a^*** |
| **Total embryos (include blastula)** | 6.22±0.72 | 4.45±0.49 | ***0.038^a^*** |
| **Good-quality** **embryos (Number)** | 4.56±0.63 | 2.84±0.34 | 0.077^b^ |

Values in italic bold indicated significant difference.

a: Estimated by Unpaired t test with Welch's correction

b: Estimated by Nonparametric test with Mann Whitney *U* test

AMH: Anti-Mullerian Hormone

EM: Endometriosis

rAFS scores: Revised American Fertility Society Score

Data are reported as means ± standard deviation

**Table 2. Indicated 141 compounds based on Lipid Oxidation Database V4.0.**

| **Index** | **Compounds** | **Abbreviation** | **CAS No** | **Class** | **KEGG ID** | **HMDB number** |
| --- | --- | --- | --- | --- | --- | --- |
| 1 | (±)14,15-epoxy-5Z,8Z,11Z-eicosatrienoic acid | 14,15-EET | 81276-03-1 | ARA | C14771 | HMDB0004264 |
| 2 | (±)11,12-epoxy-5Z,8Z,14Z-eicosatrienoic acid | 11,12-EET | 81276-02-0 | ARA | C14770 | HMDB0004673 |
| 3 | (±)8,9-epoxy-5Z,11Z,14Z-eicosatrienoic acid | 8,9-EET | 81246-85-7 | ARA | C14769 | HMDB0002232 |
| 4 | (±)5,6-epoxy-8Z,11Z,14Z-eicosatrienoic acid | 5,6-EET | 81246-84-6 | ARA | C14768 | HMDB0002190 |
| 5 | 9-oxo-10E,12Z-octadecadienoic acid | 9-oxoODE | 54232-59-6 | LA | C14766 | HMDB0004669 |
| 6 | 13-oxo-9Z,11E-octadecadienoic acid | 13-oxoODE | 54739-30-9 | LA | C14765 | HMDB0004668 |
| 7 | (±)-18-hydroxy-5Z,8Z,11Z,14Z,16E-eicosapentaenoic acid | (±)18-HEPE | 141110-17-0 | EPA | C18177 | HMDB0062222 |
| 8 | (±)-15-hydroxy-5Z,8Z,11Z,13E,17Z-eicosapentaenoic acid | (±)15-HEPE | 88852-33-9 | EPA | - | HMDB0010209 |
| 9 | (±)-12-hydroxy-5Z,8Z,10E,14Z,17Z-eicosapentaenoic acid | (±)12-HEPE | 81187-21-5 | EPA | - | HMDB0010202 |
| 10 | (±)-5-hydroxy-6E,8Z,11Z,14Z,17Z-eicosapentaenoic acid | (±)5-HEPE | 83952-40-3 | EPA | - | HMDB0005081 |
| 11 | 20-hydroxy-5Z,8Z,11Z,14Z-eicosatetraenoic acid | 20-HETE | 79551-86-3 | ARA | C14748 | HMDB0005998 |
| 12 | (±)5-hydroxy-6E,8Z,11Z,14Z-eicosatetraenoic acid | (±)5-HETE | 70608-72-9 | ARA | C04805 | HMDB0011134 |
| 13 | 8-hydroxy-5Z,9E,11Z,14Z-eicosatetraenoic acid | 8-HETE | 98462-03-4 | ARA | C14776 | HMDB0004679 |
| 14 | 8S-hydroxy-4Z,6E,10Z-hexadecatrienoic acid | tetranor-12(S)-HETE | 121842-79-3 | ARA | - | HMDB0060055 |
| 15 | 19S-hydroxy-5Z,8Z,11Z,14Z-eicosatetraenoic acid | 19(S)-HETE | 79551-85-2 | ARA | C14749 | HMDB0011136 |
| 16 | (±)18-hydroxy-5Z,8Z,11Z,14Z-eicosatetraenoic acid | (±)18-HETE | 133268-58-3 | ARA | - | HMDB0062302 |
| 17 | (±)17-hydroxy-5Z,8Z,11Z,14Z-eicosatetraenoic acid | (±)17-HETE | 128914-47-6 | ARA | - | HMDB0012598 |
| 18 | (±)16-hydroxy-5Z,8Z,11Z,14Z-eicosatetraenoic acid | (±)16-HETE | 128914-46-5 | ARA | C14778 | HMDB0004680 |
| 19 | (±)15-hydroxy-5Z,8Z,11Z,13E-eicosatetraenoic acid | (±)15-HETE | 71030-36-9 | ARA | C04742 | HMDB0003876 |
| 20 | (±)12-hydroxy-5Z,8Z,10E,14Z-eicosatetraenoic acid | (±)12-HETE | 71030-37-0 | ARA | C14777 | HMDB0006111 |
| 21 | 11S-hydroxy-5Z,8Z,12E,14Z-eicosatetraenoic acid | 11(S)-HETE | 54886-50-9 | ARA | - | - |
| 22 | (±)-9-hydroxy-5Z,7E,11Z,14Z-eicosatetraenoic acid | (±)9-HETE | 79495-85-5 | ARA | - | HMDB0010222 |
| 23 | Eicosapentaenoic Acid | EPA | 10417-94-4 | EPA | C06428 | HMDB0001999 |
| 24 | Arachidonic Acid | ARA | 506-32-1 | ARA | C00219 | HMDB0001043 |
| 25 | Docosahexaenoic Acid | DHA | 6217-54-5 | DHA | C06429 | HMDB0002183 |
| 26 | Dihomo-γ-Linolenic Acid | DGLA | 1783-84-2 | DGLA | C03242 | HMDB0002925 |
| 27 | Linoleic Acid | LA | 60-33-3 | LA | C01595 | HMDB0000673 |
| 28 | α-Linolenic Acid | ALA | 463-40-1 | ALA | C06427 | HMDB0001388 |
| 29 | γ-Linolenic Acid | GLA | 506-26-3 | GLA | C06426 | HMDB0003073 |
| 30 | 20-Carboxyarachidonic Acid | 20-COOH-AA | 79551-84-1 | ARA | - | - |
| 31 | (±)4-hydroxy-5E,7Z,10Z,13Z,16Z,19Z-docosahexaenoic acid | (±)4-HDHA/HDoHE | 90906-40-4 | DHA | - | HMDB0060049 |
| 32 | (+/-)20-Hydroxy-4Z,7Z,10Z,13Z,16Z,18E-Docosahexaenoic Acid | (±)20-HDHA/HDoHE | 90906-41-5 | DHA | - | HMDB0060048 |
| 33 | (±)7-hydroxy-4Z,8E,10Z,13Z,16Z,19Z-docosahexaenoic acid | (±)7-HDHA/HDoHE | 90780-55-5 | DHA | - | HMDB0060050 |
| 34 | 14S-hydroxy-4Z,7Z,10Z,12E,16Z,19Z-docosahexaenoic acid | (±)14-HDHA/HDoHE | 87042-40-8 | DHA | - | HMDB0060044 |
| 35 | (±)17-hydroxy-4Z,7Z,10Z,13Z,15E,19Z-docosahexaenoic acid | (±)17-HDHA/HDoHE | 90780-52-2 | DHA | - | HMDB0010213 |
| 36 | (+/-)-13-hydroxy-4Z,7Z,10Z,14E,16Z,19Z-docosahexaenoic acid | (±)13-HDHA/HDoHE | 90780-53-3 | DHA | - | HMDB0060043 |
| 37 | (+/-)-10-Hydroxy-4Z,7Z,11E,13Z,16Z,19Z-Docosahexaenoic Acid | (±)10-HDHA/HDoHE | 90780-50-0 | DHA | - | HMDB0060037 |
| 38 | (+/-)-8-Hydroxy-4Z,6E,10Z,13Z,16Z,19Z-Docosahexaenoic Acid | (±)8-HDHA/HDoHE | 90780-54-4 | DHA | - | HMDB0060051 |
| 39 | (+/-)-11-Hydroxy-4Z,7Z,9E,13Z,16Z,19Z-Docosahexaenoic Acid | (±)11-HDHA/HDoHE | 87018-59-5 | DHA | - | HMDB0060040 |
| 40 | (+/-)-16-Hydroxy-4Z,7Z,10Z,13Z,17E,19Z-Docosahexaenoic Acid | (±)16-HDHA/HDoHE | 90780-51-1 | DHA | - | HMDB0060047 |
| 41 | 4S,11R,17S-trihydroxy-5Z,7E,9E,13Z,15E,19Z-docosahexaenoic acid | RvD3 | 916888-47-6 | DHA | - | - |
| 42 | 7S,16R,17S-trihydroxy-4Z,8E,10Z,12E,14E,19Z-docosahexaenoic acid | RvD2 | 810668-37-2 | DHA | C18179 | HMDB0002294 |
| 43 | 7S,8R,17S-trihydroxy-4Z,9E,11E,13Z,15E,19Z-docosahexaenoic acid | RvD1 | 872993-05-0 | DHA | C18178 | HMDB0003733 |
| 44 | 7S,17S-dihydroxy-4Z,8E,10Z,13Z,15E,19Z-docosahexaenoic acid | RvD5 | 578008-43-2 | DHA | - | HMDB0004038 |
| 45 | 9-oxo-11α,15S-dihydroxy-prost-13E-en-1-oic acid | PGE1 | 745-65-3 | DGLA | C04741 | HMDB0001442 |
| 46 | 9α,15S-dihydroxy-11-oxo-prost-13E-en-1-oic acid | PGD1 | 17968-82-0 | DGLA | C06438 | HMDB0005102 |
| 47 | 9-oxo-11α,15S-dihydroxy-prosta-5Z,13E-dien-1-oic acid | PGE2 | 363-24-6 | ARA | C00584 | HMDB0001220 |
| 48 | 9α,15S-dihydroxy-11-oxo-prosta-5Z,13E-dien-1-oic acid | PGD2 | 41598-07-6 | ARA | C00696 | HMDB0001403 |
| 49 | 9α,11α,15S-trihydroxy-prosta-5Z,13E,17Z-trien-1-oic acid | PGF3α | 745-64-2 | EPA | C06476 | HMDB0002122 |
| 50 | 9-oxo-15S-hydroxy-5Z,10Z,13E-prostatrienoic acid | PGA2 | 13345-50-1 | ARA | C05953 | HMDB0002752 |
| 51 | 15S-hydroxy-9-oxo-5Z,8(12),13E-prostatrienoic acid | PGB2 | 13367-85-6 | ARA | C05954 | HMDB0004236 |
| 52 | 9S,15S-dihydroxy-11-oxo-5Z,13E,17Z-prostatrienoic acid | PGD3 | 71902-47-1 | EPA | C13802 | HMDB0003034 |
| 53 | 9S,11R-dihydroxy-15-oxo-13E-prostaenoic acid | 15-keto-PGF1α | 21562-58-3 | DGLA | - | HMDB0060045 |
| 54 | 9S,11R-dihydroxy-15-oxo-5Z,13E-prostadienoic acid | 15-keto-PGF2α | 35850-13-6 | ARA | C05960 | HMDB0004240 |
| 55 | 9,15-dioxo-11R-hydroxy-5Z,13E-prostadienoic acid | 15-keto-PGE2 | 26441-05-4 | ARA | C04707 | HMDB0003175 |
| 56 | 11,15-dioxo-9S-hydroxy-5Z-prostenoic acid | 13,14-dihydro-15-keto PGD2 | 59894-07-4 | ARA | - | HMDB0060042 |
| 57 | 9,15-dioxo-11R-hydroxy-5Z-prostenoic acid | 13,14-dihydro-15-keto PGE2 | 363-23-5 | ARA | C04671 | HMDB0002776 |
| 58 | 8-iso 9α,11α,15S-trihydroxy-prosta-5Z,13E-dien-1-oic acid | 8-iso-PGF2α | 27415-26-5 | ARA | C13809 | HMDB0005083 |
| 59 | 2,3-Dinor-8-Epi-Prostaglandin F2alpha | 2,3-dinor-8-iso-PGF2α | 221664-05-7 | ARA | C14794 | - |
| 60 | 6 keto-PGF1α | 6-keto-PGF1α | 58962-34-8 | ARA | C05961 | HMDB0002886 |
| 61 | 12-Hydroxy-5,8,10-heptadecatrienoic acid | 12-HHT | 54397-84-1 | ARA | C20388 | HMDB0012535 |
| 62 | tetranor-PGFM | tetranor-PGFM | 23109-94-6 | ARA | - | - |
| 63 | 9α,11α,15S-trihydroxy-prosta-5Z,13E-dien-1-oic acid | PGF2α | 551-11-1 | ARA | C00639 | HMDB0001139 |
| 64 | 9α,11α,15S-trihydroxy-prost-13E-en-1-oic acid | PGF1α | 745-62-0 | DGLA | C06475 | HMDB0002685 |
| 65 | 9,11-dioxo-15S-hydroxy-prost-13E-en-1-oic acid | PGK1 | 69413-73-6 | DGLA | - | - |
| 66 | 11-oxo-15S-hydroxy-prosta-5Z,9,13E-trien-1-oic acid | PGJ2 | 60203-57-8 | ARA | C05957 | HMDB0002710 |
| 67 | 9-oxo-15S-hydroxy-prost-13E-en-1-oic acid | 11-deoxy PGE1 | 37786-00-8 | DGLA | - | HMDB0251584 |
| 68 | 9-oxo-11S,15S-dihydroxy-5Z,13E-prostadienoic acid | 11β-PGE2 | 38310-90-6 | ARA | - | HMDB0060041 |
| 69 | 9S,11S,15S-trihydroxy-5Z,13E-prostadienoic acid | 11β-PGF2α | 38432-87-0 | ARA | C05959 | HMDB0010199 |
| 70 | 5S,12R,18R-trihydroxy-6Z,8E,10E,14Z,16E-eicosapentaenoic acid | RvE1 | 552830-51-0 | EPA | C18171 | HMDB0010410 |
| 71 | 5S-hydroxy-6R-(S-cysteinyl)-7E,9E,11Z,14Z-eicosatetraenoic acid | LTE4 | 75715-89-8 | ARA | C05952 | HMDB0002200 |
| 72 | 5S,12R-dihydroxy-6Z,8E,10E,14Z-eicosatetraenoic acid | LTB4 | 71160-24-2 | ARA | C02165 | HMDB0001085 |
| 73 | 9S,15S-Dihydroxy-11-Oxo-Thromboxa-5Z,13E-Dien-1-Oic Acid | 11-keto-TXB2 | 67910-12-7 | ARA | C05964 | HMDB0004242 |
| 74 | 9S,11,15S-trihydroxy-thrombox-13E-enoic acid | TXB1 | 64626-32-0 | DGLA | - | - |
| 75 | 5S,12R,20-trihydroxy-6Z,8E,10E,14Z-eicosatetraenoic acid | 20-OH-LTB4 | 79516-82-8 | ARA | C04853 | HMDB0001509 |
| 76 | 5S,12R-dihydroxy-6Z,8E,10E,14Z-eicosatetraene-1,20-dioic acid | 20-COOH-LTB4 | 80434-82-8 | ARA | C05950 | HMDB0006059 |
| 77 | 5S,12R-dihydroxy-6E,8E,10E,14Z-eicosatetraenoic acid | 6-trans-LTB4 | 71652-82-9 | ARA | - | HMDB0005087 |
| 78 | 5S-hydroxy-6R-(S-cysteinyl)-7E,9E,11E14Z-eicosatetraenoic acid | 11-trans-LTE4 | 75715-88-7 | ARA | - | HMDB0062286 |
| 79 | 15S-hydroxy,14R-(S-cysteinyl)-5Z,8Z,10E,12E-eicosatetraenoic acid | 14,15-LTE4 | 1000852-57-2 | ARA | - | - |
| 80 | 9α,11,15S-trihydroxythromba-5Z,13E-dien-1-oic acid | TXB2 | 54397-85-2 | ARA | C05963 | HMDB0003252 |
| 81 | 9α,11,15S-trihydroxythromba-5Z,13E,17Z-trien-1-oic acid | TxB3 | 71953-80-5 | EPA | - | HMDB0005099 |
| 82 | 5S-hydroxy-6R-(S-cysteinylglycinyl)-7E,9E,11Z,14Z-eicosatetraenoic acid | LTD4 | 73836-78-9 | ARA | C05951 | HMDB0003080 |
| 83 | 5S,6R,15S-trihydroxy-7E,9E,11Z,13E,17Z-eicosapentaenoic acid | LXA5 | 110657-98-2 | EPA | - | - |
| 84 | 5S,6R,15S-trihydroxy-7E,9E,11Z,13E-eicosatetraenoic acid | LXA4 | 89663-86-5 | ARA | C06314 | HMDB0004385 |
| 85 | 5S,14R,15S-trihydroxy-6E,8Z,10E,12E-eicosatetraenoic acid | LXB4 | 98049-69-5 | ARA | C06315 | HMDB0005082 |
| 86 | 7R,14S-dihydroxy-4Z,8E,10E,12Z,16Z,19Z-docosahexaenoic acid | 1-Mar | 1268720-28-0 | DHA | - | - |
| 87 | 10(S),17(S)-dihydroxy-4Z,7Z,11E,13Z,15E,19Z-docosahexaenoic acid | PDX | 871826-47-0 | DHA | - | - |
| 88 | (±)9,10-dihydroxy-12Z-octadecenoic acid | 9,10-DiHOME | 263399-34-4 | LA | C14828 | HMDB0004704 |
| 89 | (±)17,18-epoxy-5Z,8Z,11Z,14Z-eicosatetraenoic acid | 17(18)-EpETE | 131339-23-6 | EPA | C13843 | HMDB0010212 |
| 90 | (±)16,17-epoxy-4Z,7Z,10Z,13Z,19Z-docosapentaenoic acid | 16(17)-EpDPE | 155073-46-4 | DHA | - | HMDB0013621 |
| 91 | 5S-hydroxy-6E,8Z,11Z-eicosatrienoic acid | 5-HETrE | 195061-94-0 | MA | - | - |
| 92 | (±)5,6-dihydroxy-8Z,11Z,14Z,17Z-eicosatetraenoic acid | (±)5,6-DIHETE | 845673-97-4 | EPA | - | - |
| 93 | 5S,15S-dihydroxy-6E,8Z,10Z,13E-eicosatetraenoic acid | 5(S),15(S)-DiHETE | 82200-87-1 | ARA | - | HMDB0010216 |
| 94 | (+/-)-8,9-dihydroxy-5Z,11Z,14Z,17Z-eicosatetraenoic acid | (±)8(9)-DiHETE | 867350-87-6 | EPA | - | - |
| 95 | (+/-)-11,12-dihydroxy-5Z,8Z,14Z,17Z-eicosatetraenoic acid | (±)11(12)-DiHETE | 867350-92-3 | EPA | - | - |
| 96 | (+/-)-14,15-dihydroxy-5Z,8Z,11Z,17Z-eicosatetraenoic acid | (±)14(15)-DiHETE | - | EPA | - | HMDB0010204 |
| 97 | (+/-)-17,18-dihydroxy-5Z,8Z,11Z,14Z-eicosatetraenoic acid | (±)17(18)-DiHETE | - | EPA | - | HMDB0010211 |
| 98 | (+/-)14,15-dihydroxy-5Z,8Z,11Z-eicosatrienoic acid | (±)14(15)-DiHET | 77667-09-5 | ARA | C14775 | HMDB0002265 |
| 99 | 5-oxo-6E,8Z,11Z,14Z-eicosatetraenoic acid | 5-oxoETE | 106154-18-1 | ARA | C14732 | HMDB0010217 |
| 100 | 8S-hydroxy-9E,11Z,14Z-eicosatrienoic acid | 8(S)-HETrE | 889573-69-7 | DGLA | - | HMDB0060052 |
| 101 | 15S-hydroxy-8Z,11Z,13E-eicosatrienoic acid | 15(S)-HETrE | 13-16-1 | DGLA | - | HMDB0005045 |
| 102 | (+/-)-19(20)-Epoxy-4Z,7Z,10Z,13Z,16Z-Docosapentaenoic Acid | (±)19(20)-EpDPE(A) | - | DHA | - | HMDB0013620 |
| 103 | (+/-)-7(8)-dihydroxy-4Z,10Z,13Z,16Z,19Z-Docosapentaenoic Acid | (±)7(8)-DiHDPE(A) | - | DHA | - | - |
| 104 | (+/-)-13(14)-dihydroxy-4Z,7Z,10Z,16Z,19Z-Docosapentaenoic Acid | (±)13(14)-DiHDPE(A) | 1345275-24-2 | DHA | - | - |
| 105 | (+/-)-19(20)-dihydroxy-4Z,7Z,10Z,13Z,16Z-Docosapentaenoic Acid | (±)19(20)-DiHDPE(A) | - | DHA | - | HMDB0010214 |
| 106 | 8,9-dihydroxy-5Z,11Z,14Z-eicosatrienoic acid | (±)8(9)-DiHET | 192461-96-4 | ARA | C14773 | HMDB0002311 |
| 107 | 11,12-dihydroxy-5Z,8Z,14Z-eicosatrienoic acid | (±)11(12)-DiHET | 192461-95-3 | ARA | C14774 | HMDB0002314 |
| 108 | 9（S）,10(S),13(S)-Trihydroxy-11-Octadecenoic Acid | 9(S),10(S),13(S)-TriHOME | 29907-57-1 | LA | C14835 | HMDB0004710 |
| 109 | 9S,12S,13S-Trihydroxy-10E-Octadecenoic Acid | 9(S),12(S),13(S)-TriHOME | 97134-11-7 | LA | C14833 | HMDB0004708 |
| 110 | 15-oxo-5Z,8Z,11Z,13E-eicosatetraenoic acid | 15-oxoETE | 81416-72-0 | ARA | C04577 | HMDB0010210 |
| 111 | (+/-)-14(15)-Epoxy-5Z,8Z,11Z,17Z-Eicosatetraenoic Acid | (±)14(15)-EpETE | 131339-24-7 | EPA | - | HMDB0010205 |
| 112 | (+/-)11-Hydroxy-12E,14Z-Eicosadienoic Acid | 11-HEDE | 5598-37-8 | - | - | - |
| 113 | (+/-)-15-Hydroxy-11Z,13E-Eicosadienoic Acid | 15-HEDE | 77159-57-0 | - | - | - |
| 114 | (±)5,6-dihydroxy-8Z,11Z,14Z-eicosatrienoic acid | 5,6-DiHETrE | 213382-49-1 | ARA | C14772 | HMDB0002343 |
| 115 | (±)9,10-epoxy-12Z-octadecenoic acid | 9,10-EpOME | 16833-56-0 | LA | C14825 | HMDB0004701 |
| 116 | (±)12(13)epoxy-9Z-octadecenoic acid | 12,13-EpOME | - | LA | C14826 | HMDB0004702 |
| 117 | (8β)-5,9α,11α-trihydroxy-prosta-6E,14Z-dien-1-oic acid | 5-isoPGF2VI | - | ARA | - | - |
| 118 | 9S-hydroxy-10E,12Z,15Z-octadecatrienoic acid | 9-HOTrE | 89886-42-0 | ALA | C16326 | HMDB0010224 |
| 119 | 13S-hydroxy-9Z,11E,15Z-octadecatrienoic acid | 13-HOTrE | 87984-82-5 | ALA | C16316 | HMDB0010203 |
| 120 | 9-oxo-10E,12Z,15Z-octadecatrienoic acid | 9-OxoOTrE | 125559-74-2 | ALA | - | - |
| 121 | 13S-hydroxy-6Z,9Z,11E-octadecatrienoic acid | 13(S)-HOTrE(γ) | 74784-20-6 | GLA | - | - |
| 122 | 13S-hydroxy-9Z,11E-octadecadienoic acid | 13(S)-HODE | 29623-28-7 | LA | [C14762](http://www.genome.jp/dbget-bin/www_bget?cpd:C14762) | HMDB0004667 |
| 123 | (±)-9-hydroxy-10E,12Z-octadecadienoic acid | (±)9-HODE | 98524-19-7 | LA | - | HMDB0062652 |
| 124 | 9S-hydroperoxy-10E,12Z,15Z-octadecatrienoic acid | 9(S)-HpOTrE | 111004-08-1 | ALA | - | - |
| 125 | 9-oxo-11-(3-pentyl-2-oxiranyl)-10E-undecenoic acid | trans-EKODE-(E)-Ib | 478931-82-7 | LA | - | HMDB0246689 |
| 126 | 13S-hydroperoxy-9Z,11E-octadecadienoic acid | 13(S)-HpODE | 33964-75-9 | LA | [C04717](http://www.genome.jp/dbget-bin/www_bget?cpd:C04717) | [HMDB0003871](http://www.hmdb.ca/metabolites/HMDB0003871) |
| 127 | 12,13-dihydroxy-9Z-octadecenoic acid | (±)12(13)-DiHOME | 263399-35-5 | LA | - | - |
| 128 | 9-oxo-prosta-5Z,10,12Z,14E-tetraen-1-oic acid | 15-deoxy-Δ12,14-PGA2 | 112839-31-3 | ARA | - | - |
| 129 | (±)-11-hydroxy-5Z,8Z,12E,14Z,17Z-eicosapentaenoic acid | (±)11-HEPE | 99217-78-4 | EPA | - | - |
| 130 | 12-oxo-5Z,8Z,10E,14Z-eicosatetraenoic acid | 12-OxoETE | 108437-64-5 | ARA | [C14807](http://www.genome.jp/dbget-bin/www_bget?cpd:C14807) | [HMDB0013633](http://www.hmdb.ca/metabolites/HMDB0013633) |
| 131 | (±)-8-hydroxy-5Z,9E,11Z,14Z,17Z-eicosapentaenoic acid | (±)8-HEPE | 99217-77-3 | EPA | - | - |
| 132 | (±)-9-hydroxy-5Z,7E,11Z,14Z,17Z-eicosapentaenoic acid | (±)9-HEPE | 286390-03-2 | EPA | - | - |
| 133 | 5S,12S-dihydroxy-6Z,8E,10E,14Z-eicosatetraenoic acid | 12-epi LTB4 | 83709-73-3 | ARA | C04853 | HMDB0005089 |
| 134 | 5S,12S-dihydroxy-6E,8Z,10E,14Z-eicosatetraenoic acid | 5(S),12(S)-DiHETE | 79056-01-2 | ARA | - | - |
| 135 | 5S,12S-dihydroxy-6E,8E,10E,14Z-eicosatetraenoic acid | 6-trans-12-epi LTB4 | 71548-19-1 | ARA | - | [HMDB0005088](http://www.hmdb.ca/metabolites/HMDB0005088) |
| 136 | 8S,15S-dihydroxy-5Z,9E,11Z,13E-eicosatetraenoic acid | 8(S),15(S)-DiHETE | 80234-65-7 | ARA | - | HMDB0247570 |
| 137 | (±)7(8)-epoxy-4Z,10Z,13Z,16Z,19Z-docosapentaenoic acid | (±)7,8-EpDPE | 895127-66-9 | DHA | - | - |
| 138 | 9α,11α-dihydroxy-15-oxo-prost-5Z-en-1-oic acid | 13,14-dihydro-15-keto PGF2α | 27376-76-7 | ARA | - | [HMDB0004685](http://www.hmdb.ca/metabolites/HMDB0004685) |
| 139 | 9α,11α,15S-trihydroxy-prost-5Z-en-1-oic acid | 13,14-dihydro PGF2α | 27376-74-5 | ARA | - | HMDB0004239 |
| 140 | 9α,11α,15S,19R-tetrahydroxy-prosta-5Z,13E-dien-1-oic acid | 19(R)-hydroxy PGF2α | 64625-53-2 | ARA | - | - |
| 141 | 9α,11α,15S,20-tetrahydroxy-prosta-5Z,13E-dien-1-oic acid | 20-hydroxy PGF2α | 57930-92-4 | ARA | - | - |

**Table 3**. List of primers used in qRT-PCR, ChIP-PCR and the genome identification of GC-specifc knockout mice.

| Gene Name | Species | Sequence |
| --- | --- | --- |
| *18S* | Human | Forward 5’- CTCTTAGCTGAGTGTCCCGC -3’ |
|  |  | Reverse 5’- CTGATCGTCTTCGAACCTCC -3’ |
| *Gapdh* | Mouse | Forward 5’- GGCAAATTCAACGGCACAGT -3’ |
|  |  | Reverse 5’- GGCCTCACCCCATTTGATGT -3’ |
| *EZH2* | Human | Forward 5’- ACATCCTTTTCATGCAACACC -3’ |
|  |  | Reverse 5’- TTGGTGGGGTCTTTATCCGC -3’ |
| *Ezh2* | Mouse | Forward 5’- ACTGCTTCCTACATCCCTTCC -3’ |
|  |  | Reverse 5’- GTGCTGGGTCTGCTACTGTT -3’ |
| *EPHX2* | Human | Forward 5’- CCCGGCTTATGAAAGGAGAGA -3’ |
|  |  | Reverse 5’- CCATCTCTCTCAGCACGGTC -3’ |
| *Ephx2* | Mouse | Forward 5’- GGTGCCCTGTCCATACGTC -3’ |
|  |  | Reverse 5’- ACGGGCTGGCTTCAGATTAC -3’ |
| *CYP3A5* | Human | Forward 5’- CTCCTCTATCTATATGGGACCCG -3’ |
|  |  | Reverse 5’- AGTCGGTGCTTTTGTTTGTCG -3’ |
| ChIP-PCR-Primer 1 | Mouse | Forward 5’- TTCAGACTGGGGCTTGGTTG -3’ |
|  |  | Reverse 5’- CCTCCACCCAAATTGTCTATGC -3’ |
| ChIP-PCR- Primer 2 | Mouse | Forward 5’- TGGAGGTCACTGATGGGTTC -3’ |
|  |  | Reverse 5’- CCTTCCAGACCAACTGAGGT -3’ |
| GP-*Ezh2* | Mouse  (Genome) | Forward 5’- CATGTGCAGCTTTCTGTTCA -3’ |
|  |  | Reverse 5’- CACAGCCTTTCTGCTCACTG -3’ |
| GP-*Cyp19a1*-WT | Mouse (Genome) | Forward 5’- AAATGAGGACAGGCACCTTG -3’ |
| GP- *Cyp19a1*-Mutation | Mouse (Genome) | Forward 5’- GAAACAGGGGCAATGGTG -3’ |
| GP- *Cyp19a1*-Common | Mouse (Genome) | Reverse 5’- CGGATAAGTAATGCCCCAGA -3’ |

**Table 4**. Antibodies used in western blot and chromatin immunoprecipitation.

| Antigen | Catalog number | Dilution in WB | Dilution in ChIP | Producer | Country |
| --- | --- | --- | --- | --- | --- |
| GAPDH | 60004-1-Ig | 1:10000 | / | Proteintech | USA |
| EZH2 | 5246 | 1:1000 | / | CST | USA |
| H3K27Me3 | 9733 | 1:1000 | 1:50 | CST | USA |
| EPHX2 | ab155280 | 1:1000 | / | Abcam | UK |
| γ-H2A.X | 9718 | 1:1000 for WB  1:200 for IF | / | CST | USA |
| P21 | 2947 | 1:1000 | / | CST | USA |
| P21 | ab188224 | 1:1000 for mice | / | Abcam | UK |
| PI3K p85 | ab191606 | 1:1000 | / | Abcam | UK |
| AKT | ab179463 | 1:1000 | / | Abcam | UK |
| p-AKT | ab192623 | 1:600 | / | Abcam | UK |
| mTOR | ab134903 | 1:1000 | / | Abcam | UK |
| p-mTOR | ab109268 | 1:500 | / | Abcam | UK |
